# Melanoma: Does It Present Differently in Darker Skin Tones?

**DOI:** 10.15766/mep_2374-8265.11311

**Published:** 2023-05-09

**Authors:** Brandon Thompson, Toni Jenkins, John Paul Sánchez, Matthew Frederick, Alba Posligua-Alban, Naiara Sbroggio Barbosa

**Affiliations:** 1 Registered Nurse, Neuroscience Intensive Care Unit, University of New Mexico Hospital; 2 Third-Year Medical Student, Howard University College of Medicine; 3 Vice Chair for DEI, Department of Emergency Medicine, University of New Mexico School of Medicine, and Executive Associate Vice Chancellor, Health Sciences Center Office for Diversity, Equity and Inclusion, University of New Mexico School of Medicine; 4 Assistant Professor, Department of Emergency Medicine, University of New Mexico School of Medicine; 5 Third-Year Resident, Department of Dermatology, University of New Mexico School of Medicine; 6 Assistant Professor of Dermatology, Mayo Clinic College of Medicine and Science; † Co-primary author

**Keywords:** Melanoma, Skin Tone, Case-Based Learning, Cultural Competence, Dermatology, Anti-racism, Diversity, Equity, Inclusion

## Abstract

**Introduction:**

There are vast differences in clinical presentations of melanoma across skin tones. Individuals with darker skin tones tend to have a higher prevalence of advanced-stage melanoma, which correlates with increased mortality. We designed this interactive workshop to increase nursing and medical trainees’ awareness of the epidemiology, prevention, and treatment of melanoma in individuals of darker skin tones.

**Methods:**

The Kern model was used in the design, implementation, and evaluation of the workshop. The 75-minute workshop consisted of a PowerPoint presentation, video-based reflection activities, and case studies. Evaluation consisted of pre- and postworkshop questionnaires. The workshop was implemented two times among 63 nursing students, 11 medical students/residents, and six medical faculty.

**Results:**

Seventy-one participants completed the pre- and postworkshop evaluations. A comparison of pre- and postworkshop responses utilizing the Wilcoxon matched-pair signed rank test showed a statistically significant increase in learners’ confidence to address each learning objective.

**Discussion:**

Through this interactive educational presentation, medical and nursing trainees can gain heightened awareness of melanoma across various skin tones, especially unique presentations in darker skin tones.

## Educational Objectives

By the end of this activity, learners will be able to:

1. Describe the structure and components of the skin.
2. Describe the etiology and clinical manifestation of melanoma and its subtypes.
3. Recognize various melanoma presentations, especially in darker skin tones.
4. List melanoma prevention and treatment options.

## Introduction

The incidence of melanoma continues to rise despite efforts at educating practitioners and the public about its causes and prevention strategies.^1^ Most educational programs are focused on the subset of the population with the highest incidence: individuals of light skin tones. However, melanoma occurs as well in individuals of darker skin tones.^1^

Historically, melanoma research has been framed by racial differences and by skin tone differences. Prior research focusing on melanoma by racial groups noted that approximately 1% of skin cancers occur in Black-identified patients and that mortality for Black-identified individuals is two- to threefold higher than for White-identified populations.^2^ A significant factor in racial disparities is advanced stage at diagnosis.^3,4^ When comparing by skin tone using the Fitzpatrick skin scale in a dichotomous fashion, Fitzpatrick type I-III skin is often labeled as Caucasian and Fitzpatrick type IV-VI as skin of color individuals (SCIs).^5^ Research on melanoma reported by skin tone has shown that practitioners (a) tend to be unaware that 70% of melanomas in SCIs arise on the often-overlooked plantar surface of the foot and (b) lack proficiency in differentiating benign from malignant lesions in SCIs.^6,7^ In addition, SCIs may not be appropriately counseled by practitioners to get routine skin exams. The knowledge gap in this area has been identified by some experts as “a prime example of structural inequalities in medicine.”^8^

In response to the lack of dermatologic images of darker skin tones, a growing body of print literature and atlases has been developed, including Taylor and Elbuluk's *Color Atlas and Synopsis for Skin of Color,*^9^ Jackson-Richards and Pandya's *Dermatology Atlas for Skin of Color,*^10^ and Eleryan and Friedman's *A Diverse and Inclusive Atlas,*^11^ among others. Online resources that feature diverse skin tones include educational materials from the American Academy of Dermatology Skin of Color Curriculum^12^ and the Skin of Color Society Dermatology E-Learning + Equity Platform,^13^ as well as online atlases published by VisualDX.com^14^ and the University of New Mexico.^15^

From 2016 to 2021, *MedEdPORTAL* published four educational resources referencing *melanoma* as a keyword. One of these four uses general dermatology and musculoskeletal medicine to advance medical Spanish communication skills of medical students.^16^ The other three resources focus on addressing general dermatology knowledge among preclinical medical students and novice health care workers.^17–19^ All four publications were implemented at single sites. There are no materials currently in *MedEdPORTAL* addressing the important differences in presentation of melanoma amongst SCIs.

Our 75-minute interactive workshop aims to help learners (i.e., nursing students, medical students, and residents) understand that patients with darker skin tones (a) can develop melanoma, (b) may have a presentation different from those with lighter skin tones, and (c) tend to have more advanced/aggressive melanoma at the time of diagnosis. We utilized the Kern model of curriculum development in the creation of our content.^20^ A needs assessment revealed a paucity of educational material on this topic.^21^ Our workshop can help fulfill medical school and residency requirements, such as Liaison Committee on Medical Education element 3.3 Diversity/Pipeline Programs and Partnership and element 7.6 Cultural Competence and Health Care Disparities^22^; ACGME Interpersonal and Communication Skills Competence^23^; and the AAMC's Cultural Competence Education Domains I and IV^24^ and Diversity, Equity, and Inclusion Competencies Ia and Ib.^25^

## Methods

A nursing student, a medical student, a dermatology resident, a dermatology attending, and two emergency medicine faculty developed, implemented, and/or evaluated this workshop. The students conceptualized the workshop, and the faculty provided guidance on clinical components and health disparities content. Authors Brandon Thompson, Toni Jenkins, and Naiara Sbroggio Barbosa copresented the workshop on two occasions: at the Building the Next Generation of Academic Physicians (BNGAP) Medical Education Conference and at the Nursing Program of Central New Mexico Community College.

The workshop employed three instructional strategies: an interactive Microsoft PowerPoint (PPT) presentation, reflection exercises, and case discussion. The PPT presentation (Appendix A) began with a brief overview of what melanoma is and how it affects individuals with darker skin tones. To highlight the spectrum of skin tone and distinguish skin tone from racial categories, we asked participants to self-identify utilizing a pigmentary phototype (the Fitzpatrick scale). An interview clip (Appendix B) addressing myths related to pigmented skin was incorporated into the presentation. The PPT proceeded with an outline of the epidemiology of skin cancer in SCIs as well as a basic overview of dermatopathology. Attention then turned to the clinical presentation of melanoma in SCIs, with multiple photographic references for both malignant and benign lesions. The workshop also included two case discussions for participants to apply new knowledge while discussing health disparities, risk factors, and prevention of melanoma. A facilitator guide (Appendix C) described how to implement each component of the workshop. Participants were asked to complete pre- and postworkshop self-assessments (Appendix D) based on the presentation's learning objectives and content.

The workshop took 75 minutes to implement. Approximately a third of the time was spent on interactive reflection and case discussions (25 minutes), with the rest dedicated to didactic material and administration of the pre- and postworkshop evaluations.

Materials included audiovisual equipment, virtual technology, and online (or, if in person, printed) evaluation forms. We implemented both sessions virtually due to COVID restrictions.

Both pre- and postworkshop questionnaires contained self-assessments of confidence and knowledge questions to coincide with Kirkpatrick levels 1 and 2 of evaluation. Participants’ confidence in meeting the workshop's learning objectives was assessed on a 5-point scale (1 = *no confidence,* 5 = *complete confidence*). The preworkshop questionnaires contained demographic questions, whereas the postworkshop questionnaires contained qualitative feedback questions. The survey was created by the coauthors and did not undergo validity testing. Descriptive and nonparametric bivariate analyses were conducted using SPSS version 28.0.

Future facilitators should have a general knowledge of basic skin anatomy and dermatopathology. They should consider setting aside 2–3 hours to review the PPT presentation, video, cases, and evaluation forms.

The University of New Mexico School of Medicine Human Research Protections Office granted institutional review board exempt status.

## Results

There were 80 participants total, 17 attending the BNGAP Medical Education Conference workshop and 63 attending the workshop at Central New Mexico Community College. Seventy-one participants completed the pre- and postworkshop questionnaire, making the response rate 89%.

Participants’ roles were diverse, with six faculty, eight medical students, three residents, and 63 nursing students. Seventy percent identified as women. Among those who reported their race or ethnicity, 45% (*n* = 36) identified as Latina/o/x/e, Hispanic, or of Spanish origin+; 9% (*n* = 7) as African American/Black; 38% (*n* = 30) as White; 6% (*n* = 5) as Asian; and 1% (*n* = 1) as American Indian/Alaska Native.

The Wilcoxon matched-pair signed rank test showed a statistically significant increase when comparing learners’ pre- and postworkshop confidence in addressing each learning objective. The results are depicted in the Table.

**Table.**
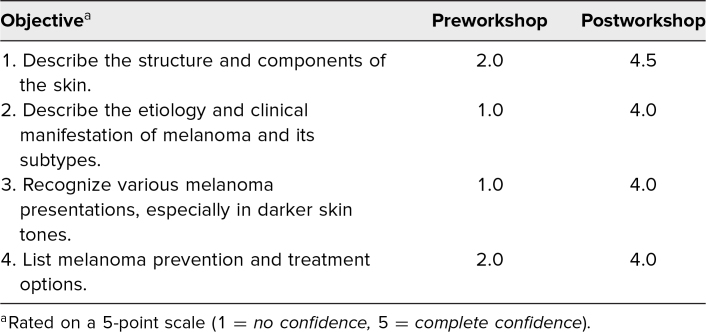
Participants’ Confidence in Meeting Learning Objectives

In terms of qualitative feedback on the postworkshop evaluation form, several respondents noted that they liked the interactive nature of the session and the different teaching modalities. Regarding improvements, participants desired additional skin of color images and a larger font on several slides.

## Discussion

We successfully designed and implemented a workshop to help health professional trainees—in particular, nurses, medical students, and residents—understand that SCI patients can develop melanoma and that these melanomas are usually more advanced/aggressive at the time of diagnosis. The evaluations received were overall positive. Our data revealed that learners had an increase in confidence when identifying melanoma in darker skin tones. We hope that by employing the information gained in this workshop, practitioners can perform more careful dermatologic examinations in SCIs (thereby increasing the likelihood of diagnosing melanoma at an earlier stage) as well as educate their SCI patients on the need for continued skin examinations as part of routine health care.

This resource addresses two critical needs within nursing and medical education. First, there are currently no *MedEdPORTAL* publications created with the objective of increasing awareness of melanoma in SCIs. We find this lack of educational material to represent an important gap in dermatology curricula, especially in the context of a high-morbidity disease process that presents uniquely in SCIs. While the typical demographic affected by melanoma is lighter-pigmented individuals (Fitzpatrick types I-III), the morbidity in SCIs is significantly higher and is diagnosed at later stages.^2^ This strongly suggests that further educational material needs to be developed for health professionals and the public.

A second important contribution our material makes is in addressing structural inequalities in health professions education and research language. In researching the topic of melanoma in SCIs, we found a trend towards generalizing racial groups without respect for the heterogeneity of pigmentation. Large swaths of people were referred to collectively as Black, Hispanic, or Asian despite the imprecision of such designations. In our material, we have attempted to define SCIs more clearly by use of the Fitzpatrick scale. Continued penetration of more precise descriptions of pigmented skin as well as recognition of self-identification can better align the literature with diversity, equity, and inclusion–focused health care. Notably, the original use of the Fitzpatrick scale was to describe the reaction of lighter-pigmented Caucasian skin to UV therapy, and so, further development and implementation of an SCI-centric scale are needed.^26^

One main challenge to the creation and implementation of this workshop is being aware of how race, ethnicity, skin tone, and skin pathology are often conflated in the research literature and potentially during discussions of the epidemiology of melanoma. We encourage facilitators to help workshop participants review and use terms such as racial categories and skin of color appropriately rather than considering them synonymous.

There are several limitations associated with this workshop. The results should not be overgeneralized to indicate that the workshop would yield the same increase in confidence among all medical students, residents, faculty, or nursing students. Our sample was relatively small, with a large representation of nursing students. The workshop is a brief intervention and may not yield sustained confidence in meeting the learning objectives. The workshop has not been designed to assess for changes in knowledge or behavior, components critical to reducing clinical outcomes. Nor has the workshop been trialed using a variety of facilitator knowledge levels; therefore, we suggest that future facilitators have a foundation in basic skin anatomy and dermatopathology. In terms of our evaluation tool, we did not label each response on the 5-point scale, only the anchors. Artino and Gehlbach suggest that all response options, especially for a 5-point scale, should be labeled to accurately measure what is intended to be measured.^27^

Despite these limitations, participants’ feedback demonstrated that the workshop structure and content were effective at raising awareness of melanoma and its various presentations. Learners commended the use of videos, reflection exercises, and active interaction through the case discussion. Modifications for improvement of the material in the future could include more images as well as continued efforts to deconstruct race-oriented language in favor of objective pigmentation scales.

## Appendices


Melanoma Presentation.pptxMelanoma Myth.mp4Facilitator Guide.docxEvaluation Form.docx
*All appendices are peer reviewed as integral parts of the Original Publication.*
