# Supplementary material for: Melanoma: Does It Present Differently in Darker Skin Tones?
Source: MedEdPORTAL. 2023 May 9;19:11311. doi: 10.15766/mep_2374-8265.11311 (PMC10166772; doi:10.15766/mep_2374-8265.11311)
Supplement: Supplementary file 1 — Melanoma Presentation.pptxMelanoma Myth.mp4Facilitator Guide.docxEvaluation Form.docx [file mep_2374-8265.11311-s001.zip › A. Melanoma Presentation.pptx]

## Slide 1
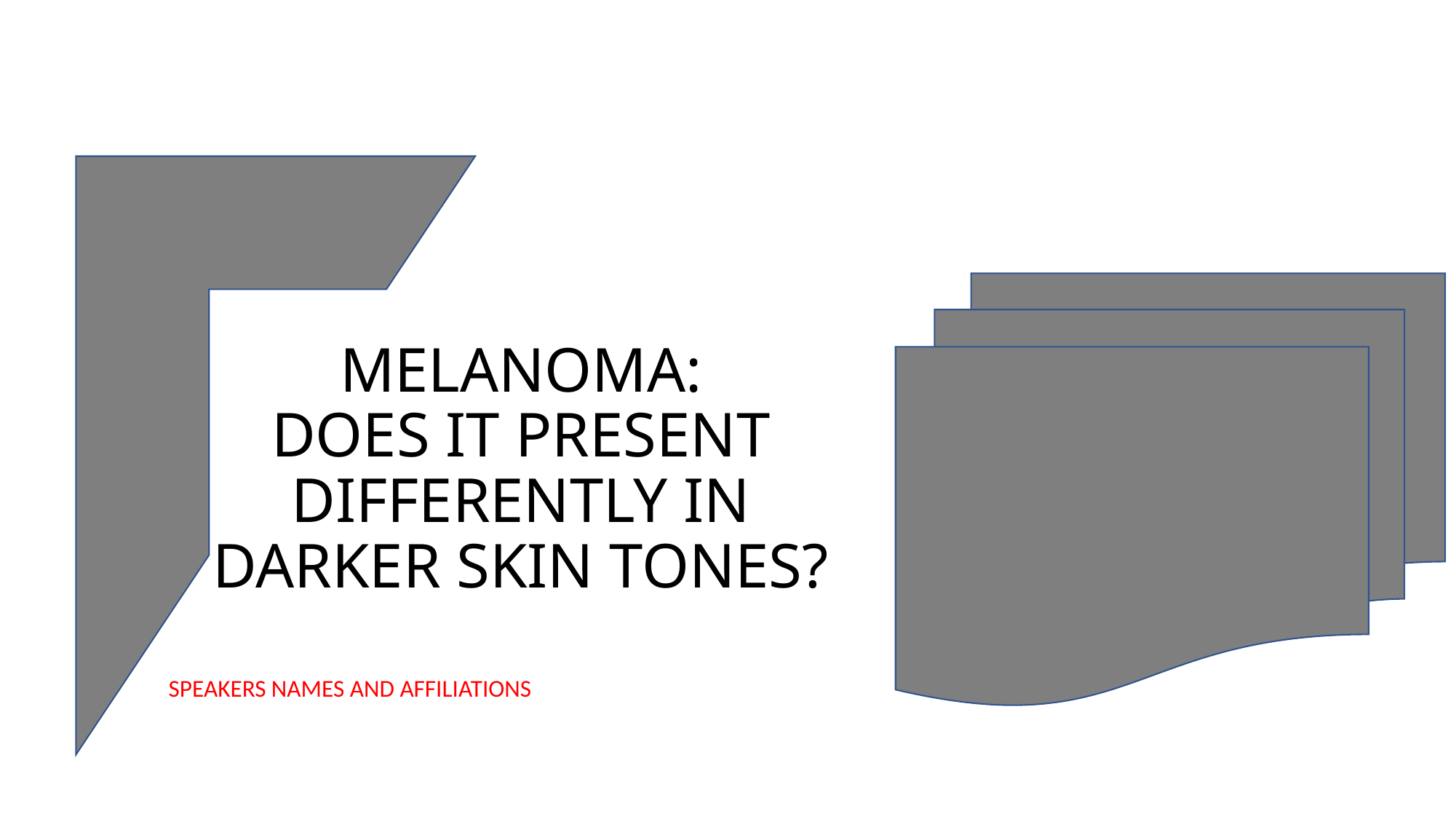

MELANOMA:
DOES IT PRESENT DIFFERENTLY IN DARKER SKIN TONES?
SPEAKERS NAMES AND AFFILIATIONS

## Slide 2
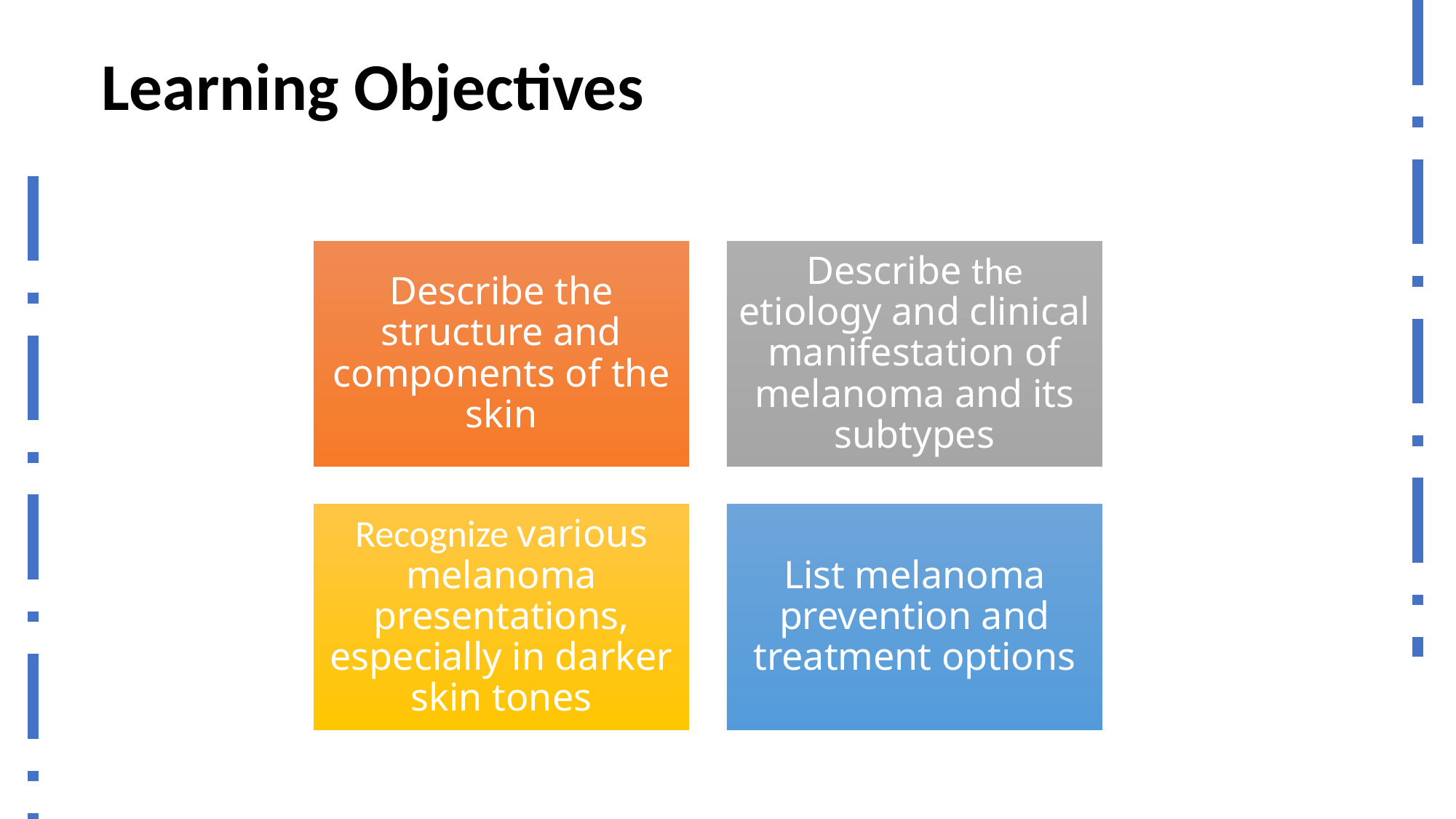

# Learning Objectives
Describe the structure and components of the skin
Describe the etiology and clinical manifestation of melanoma and its subtypes
Recognize various melanoma presentations, especially in darker skin tones
List melanoma prevention and treatment options

## Slide 3
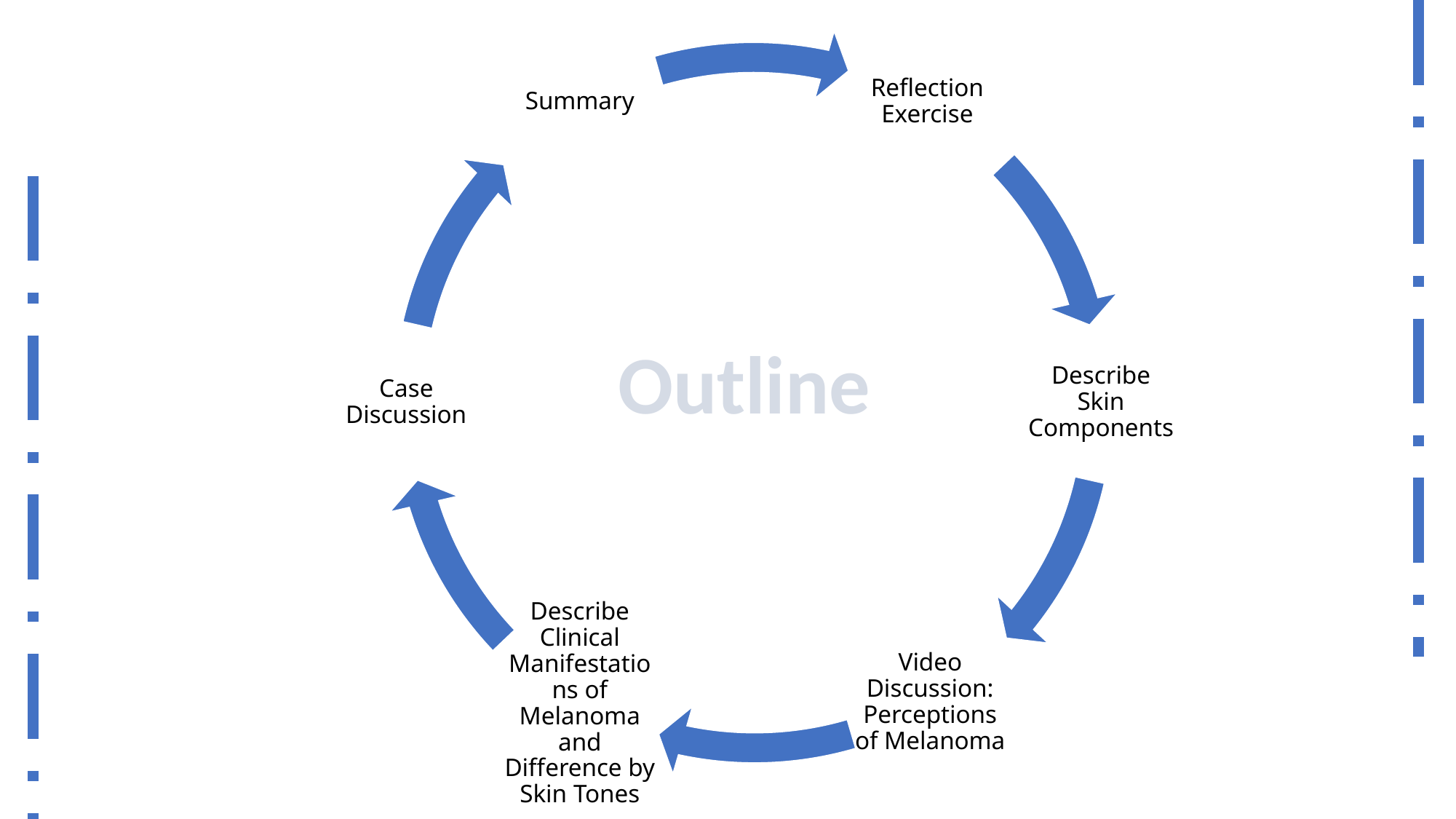

Summary
Reflection Exercise
Case Discussion
Describe Skin Components
Video Discussion: Perceptions of Melanoma
Describe Clinical Manifestations of Melanoma and Difference by Skin Tones
Outline

## Slide 4
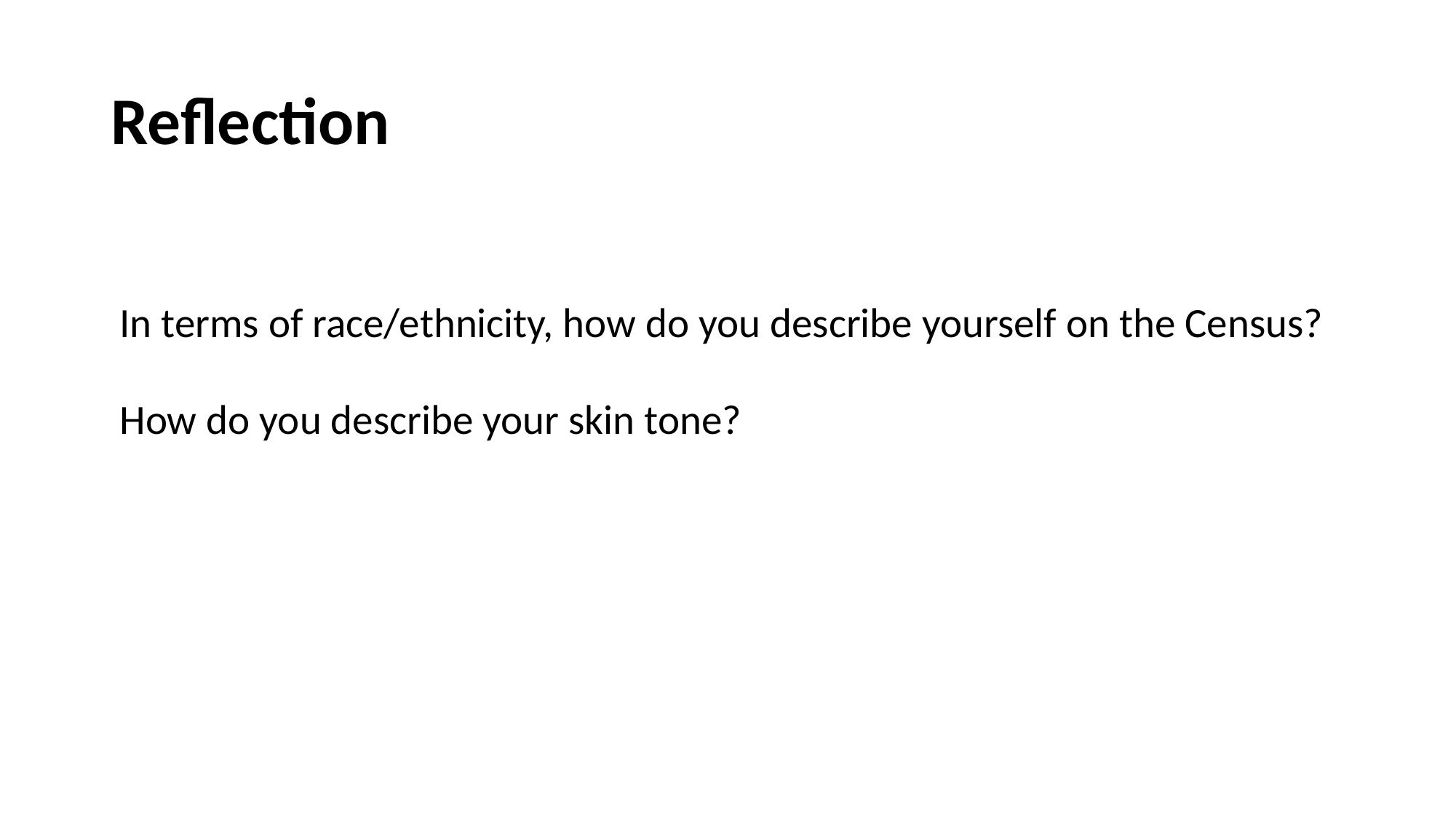

# Reflection
In terms of race/ethnicity, how do you describe yourself on the Census?
How do you describe your skin tone?

## Slide 5
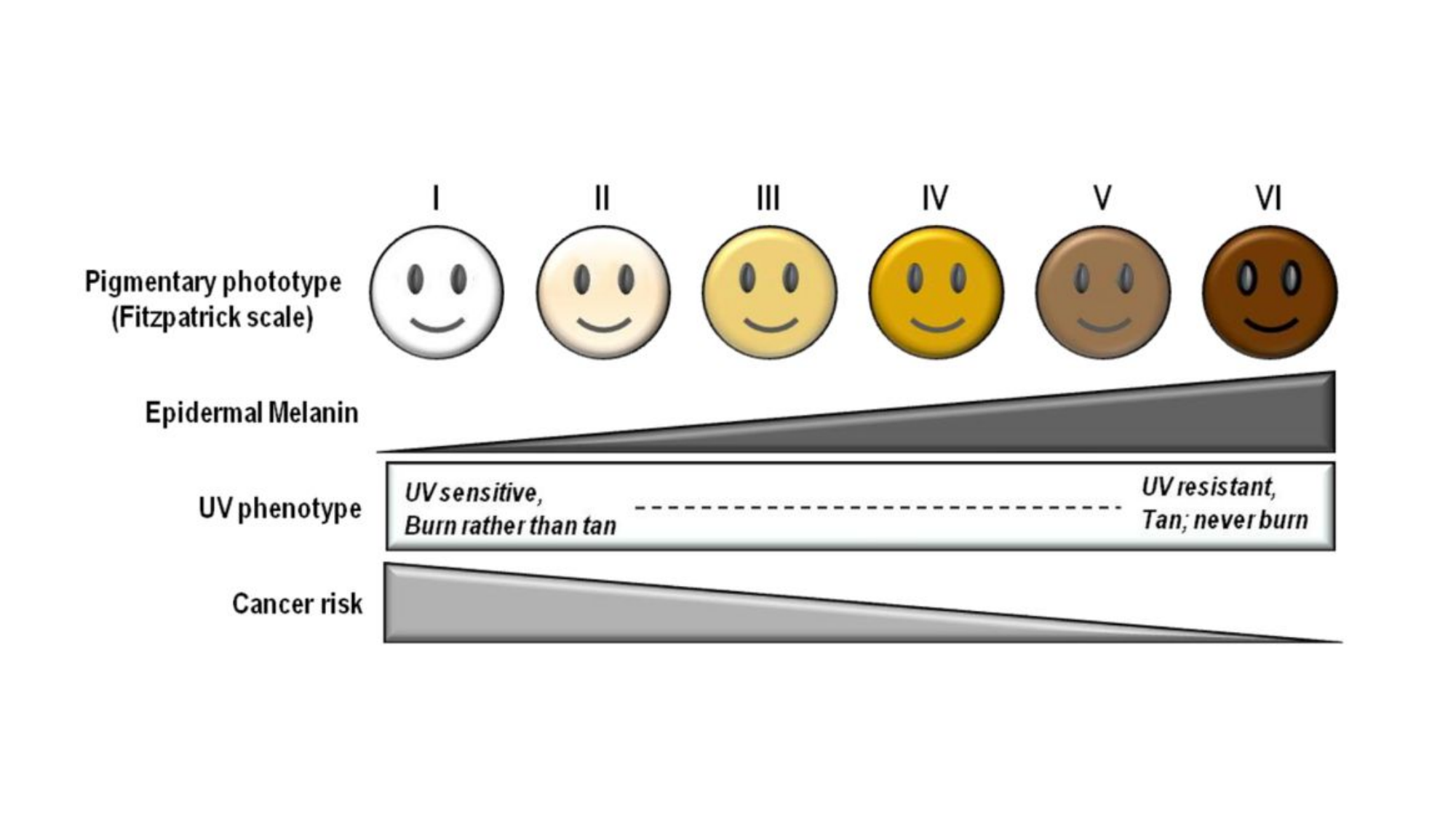

## Slide 6
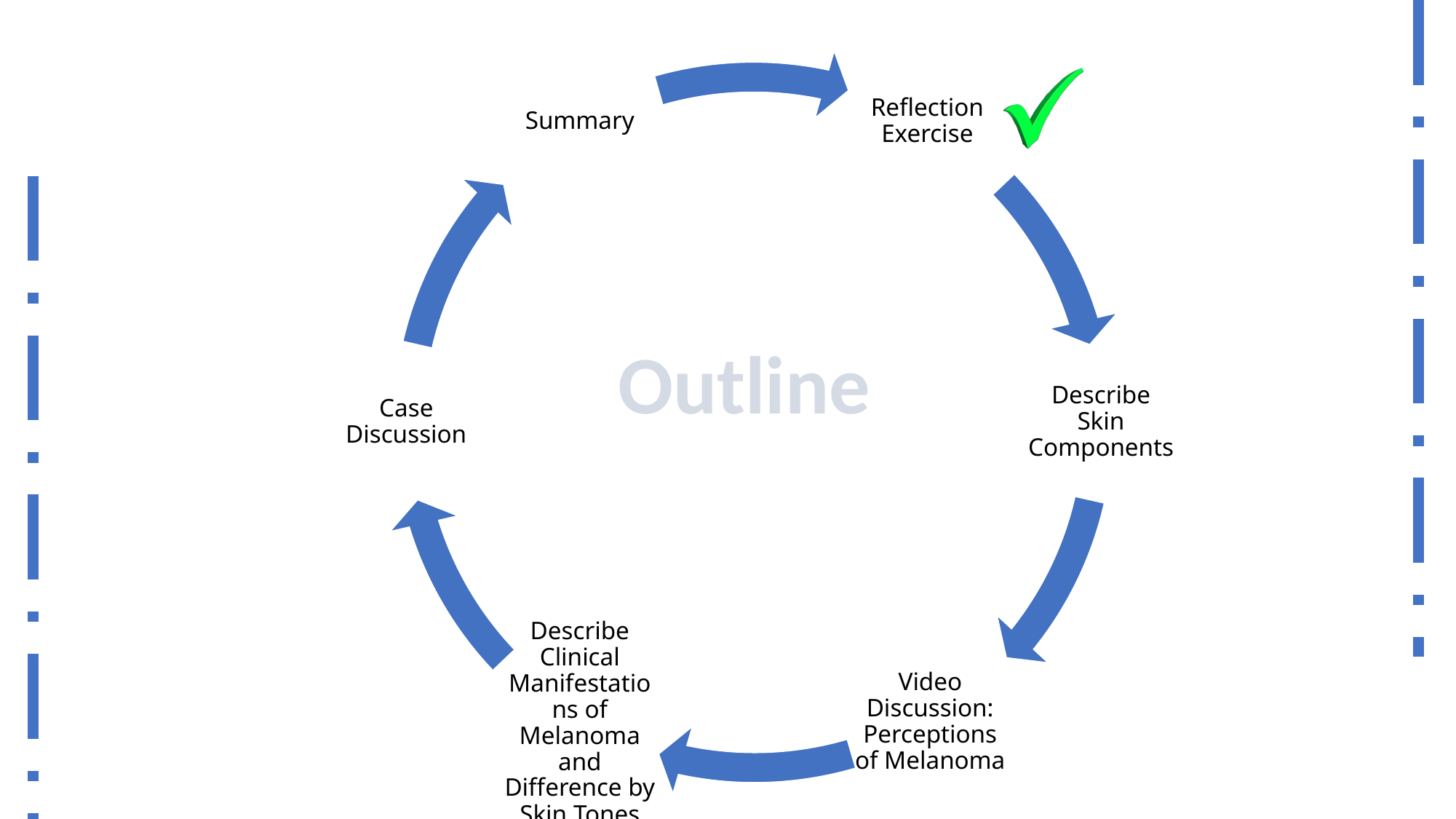

Summary
Reflection Exercise
Case Discussion
Describe Skin Components
Video Discussion: Perceptions of Melanoma
Describe Clinical Manifestations of Melanoma and Difference by Skin Tones
Outline

## Slide 7
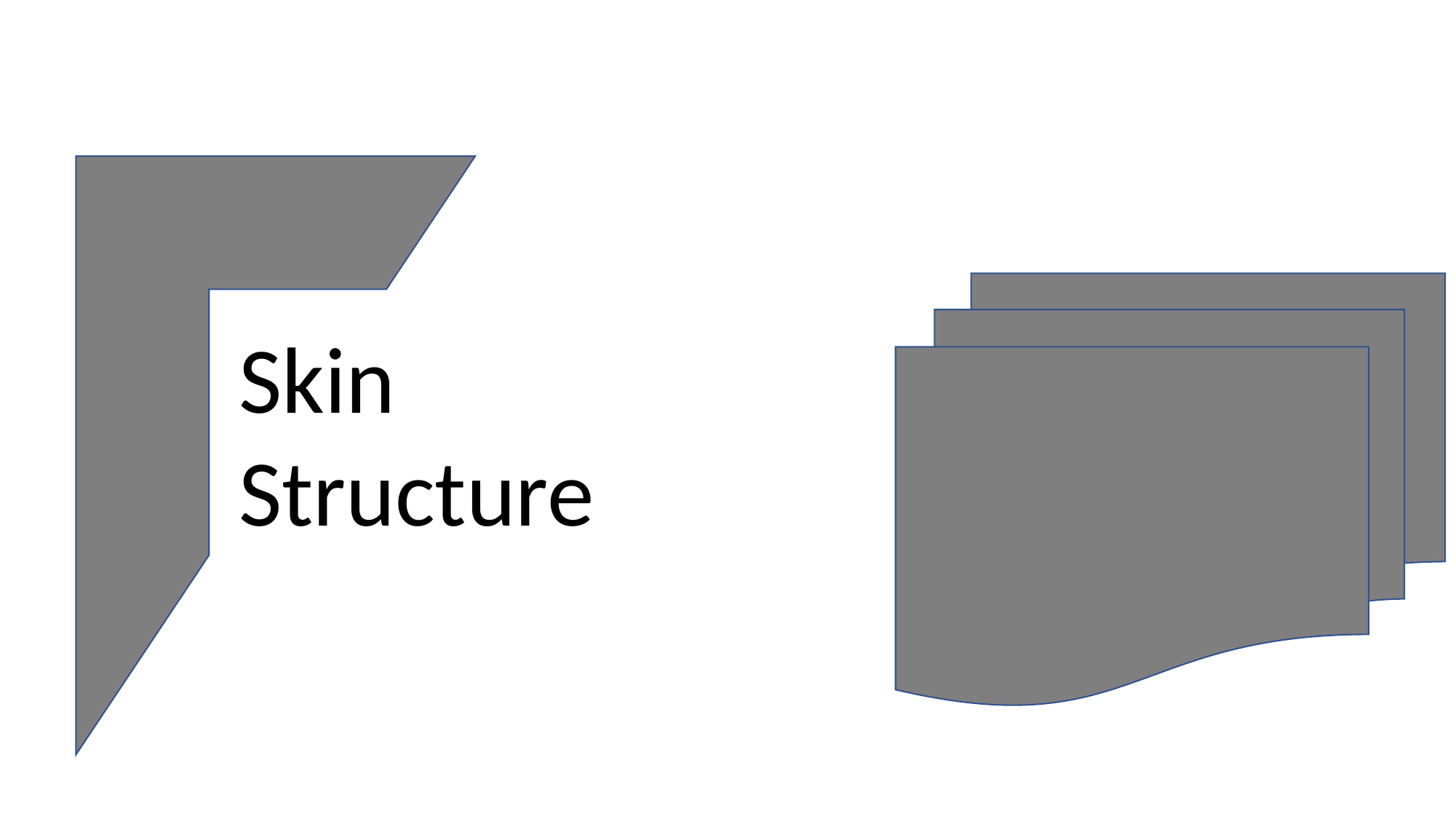

Skin Structure

## Slide 8
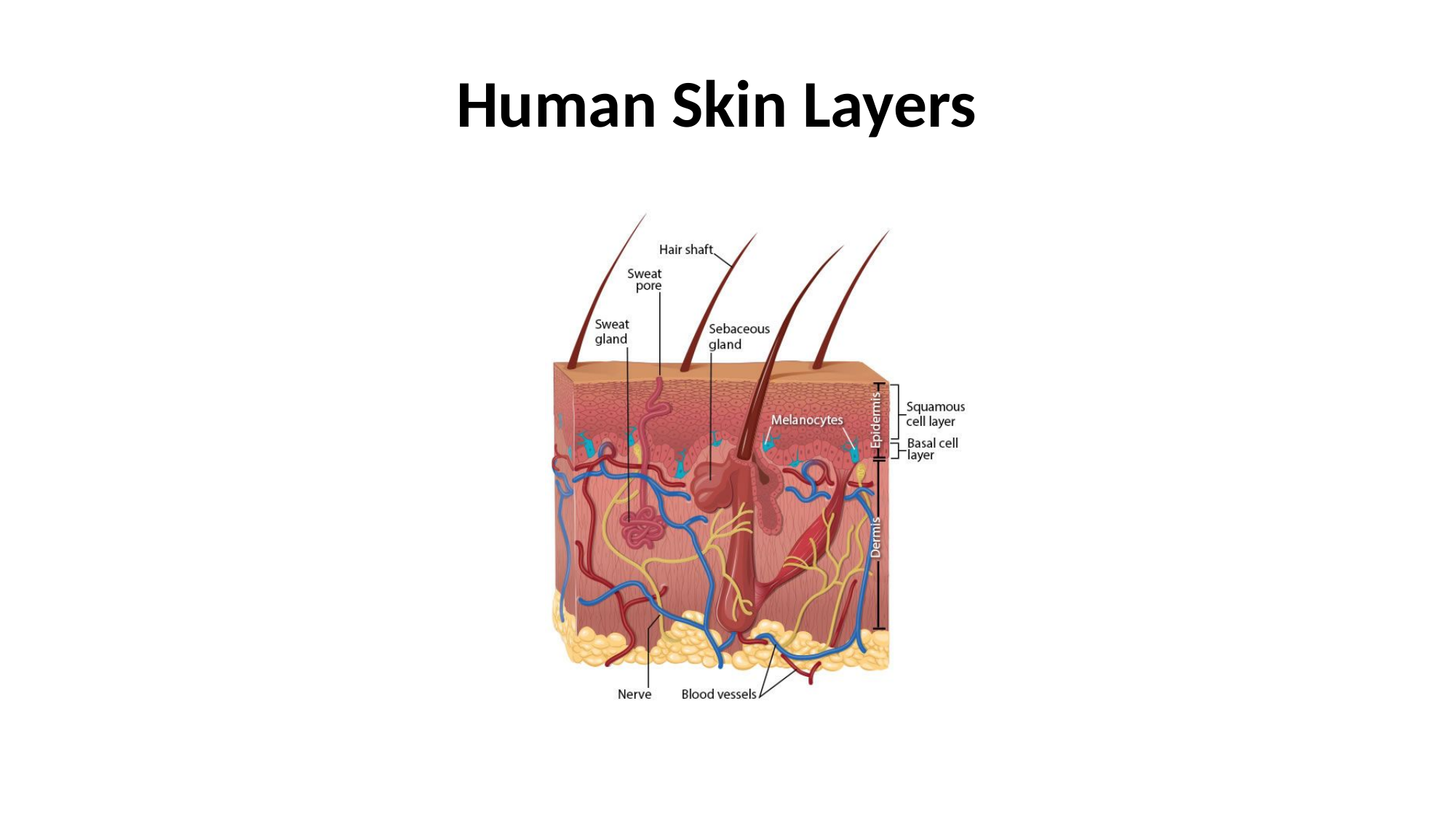

Human Skin Layers

## Slide 9
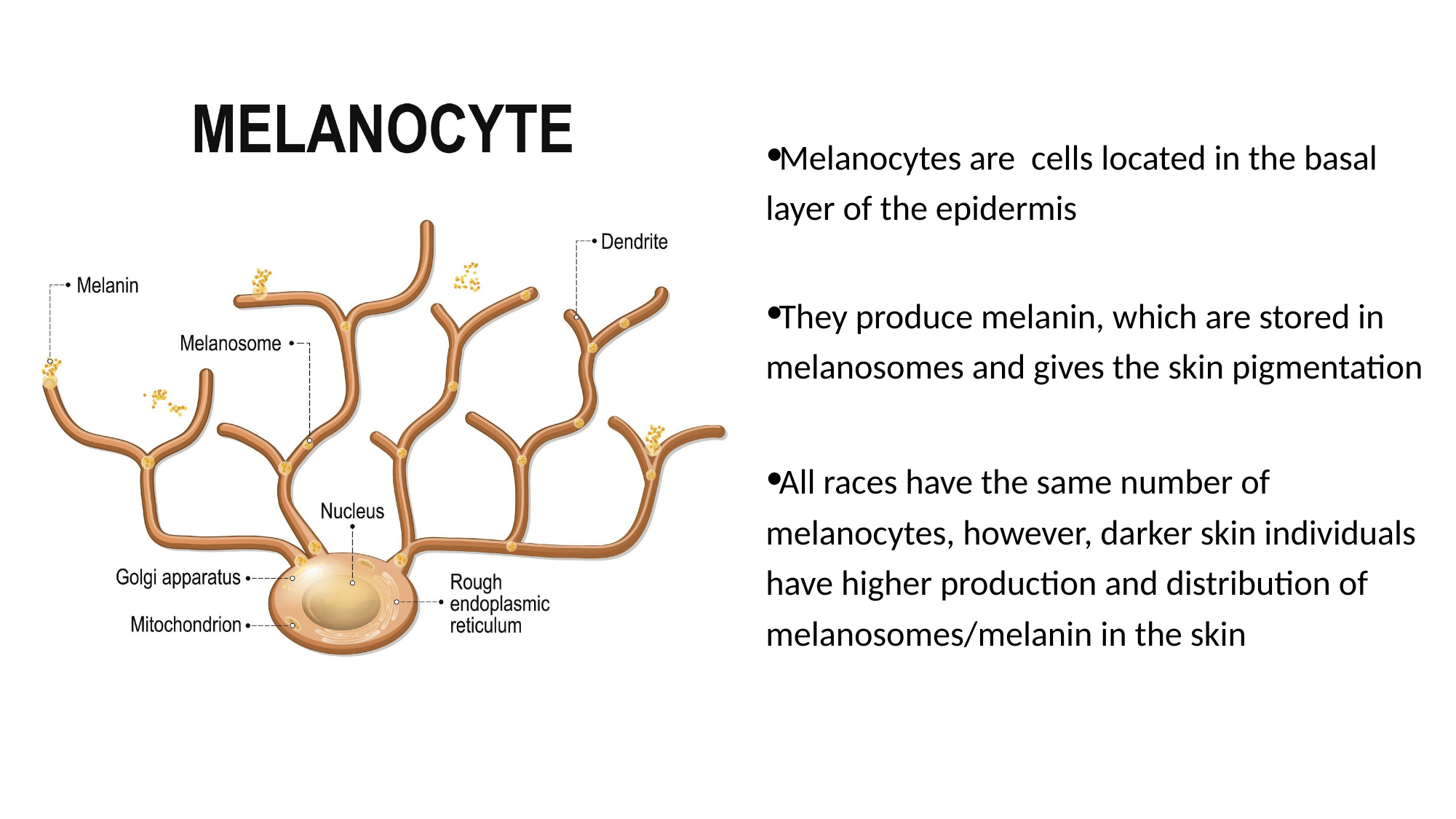

Melanocytes are cells located in the basal layer of the epidermis
They produce melanin, which are stored in melanosomes and gives the skin pigmentation
All races have the same number of melanocytes, however, darker skin individuals have higher production and distribution of melanosomes/melanin in the skin

## Slide 10
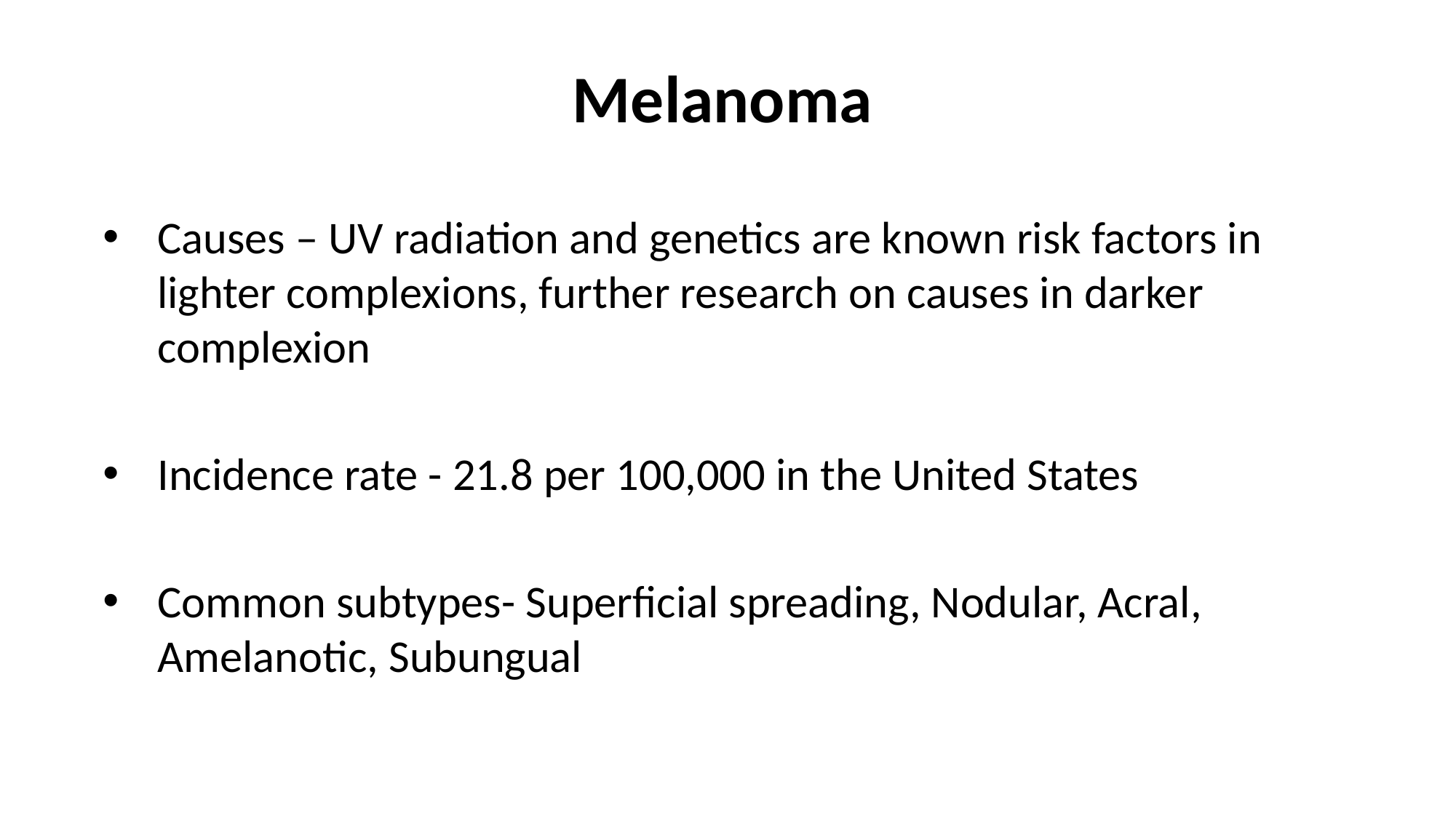

Melanoma
Causes – UV radiation and genetics are known risk factors in lighter complexions, further research on causes in darker complexion
Incidence rate - 21.8 per 100,000 in the United States
Common subtypes- Superficial spreading, Nodular, Acral, Amelanotic, Subungual
MELANOMA OVERVIEW

## Slide 11
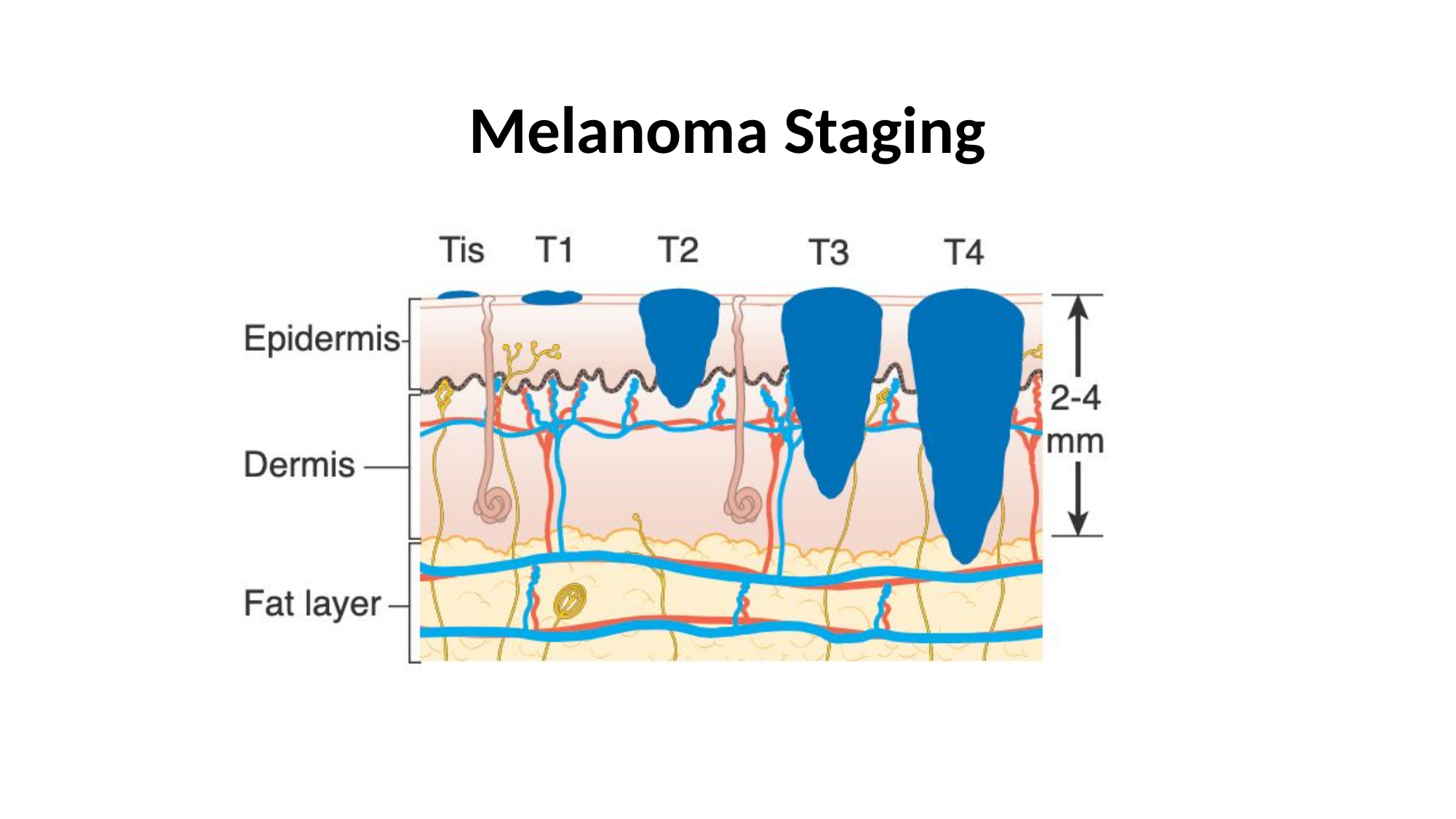

Melanoma Staging

## Slide 12
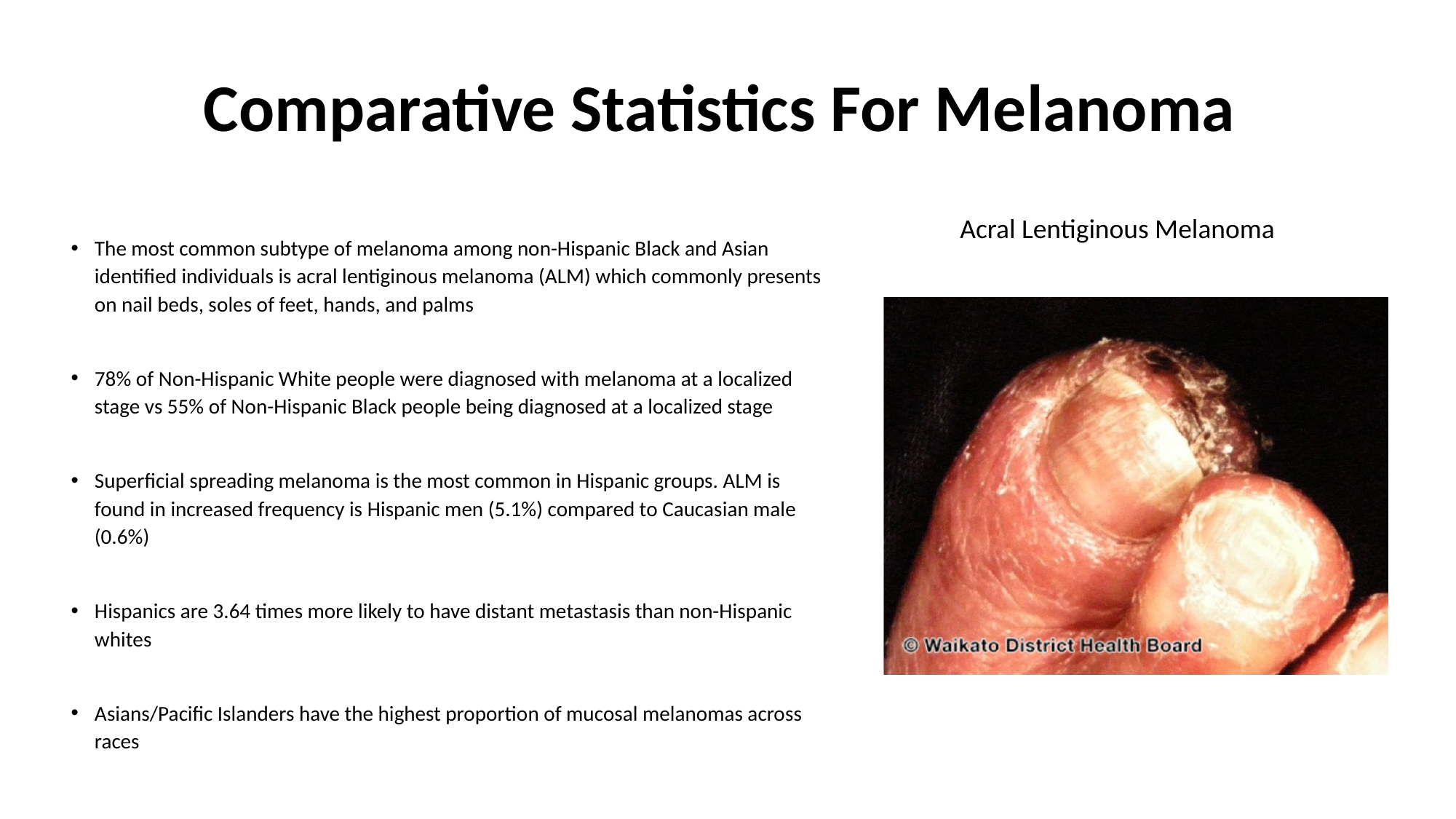

Comparative Statistics For Melanoma
The most common subtype of melanoma among non-Hispanic Black and Asian identified individuals is acral lentiginous melanoma (ALM) which commonly presents on nail beds, soles of feet, hands, and palms
78% of Non-Hispanic White people were diagnosed with melanoma at a localized stage vs 55% of Non-Hispanic Black people being diagnosed at a localized stage
Superficial spreading melanoma is the most common in Hispanic groups. ALM is found in increased frequency is Hispanic men (5.1%) compared to Caucasian male (0.6%)
Hispanics are 3.64 times more likely to have distant metastasis than non-Hispanic whites
Asians/Pacific Islanders have the highest proportion of mucosal melanomas across races
Acral Lentiginous Melanoma

## Slide 13
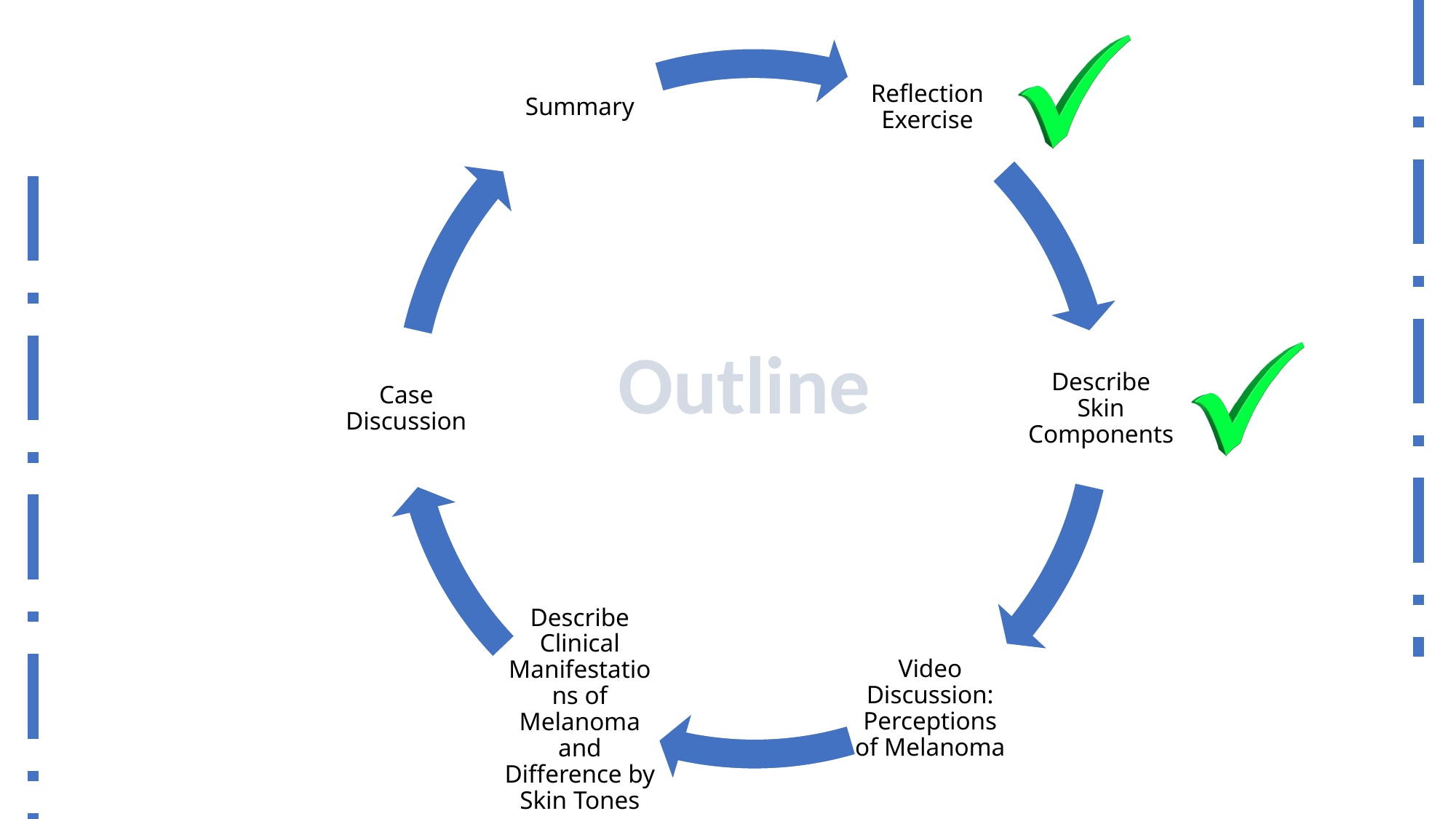

Summary
Reflection Exercise
Case Discussion
Describe Skin Components
Video Discussion: Perceptions of Melanoma
Describe Clinical Manifestations of Melanoma and Difference by Skin Tones
Outline

## Slide 14
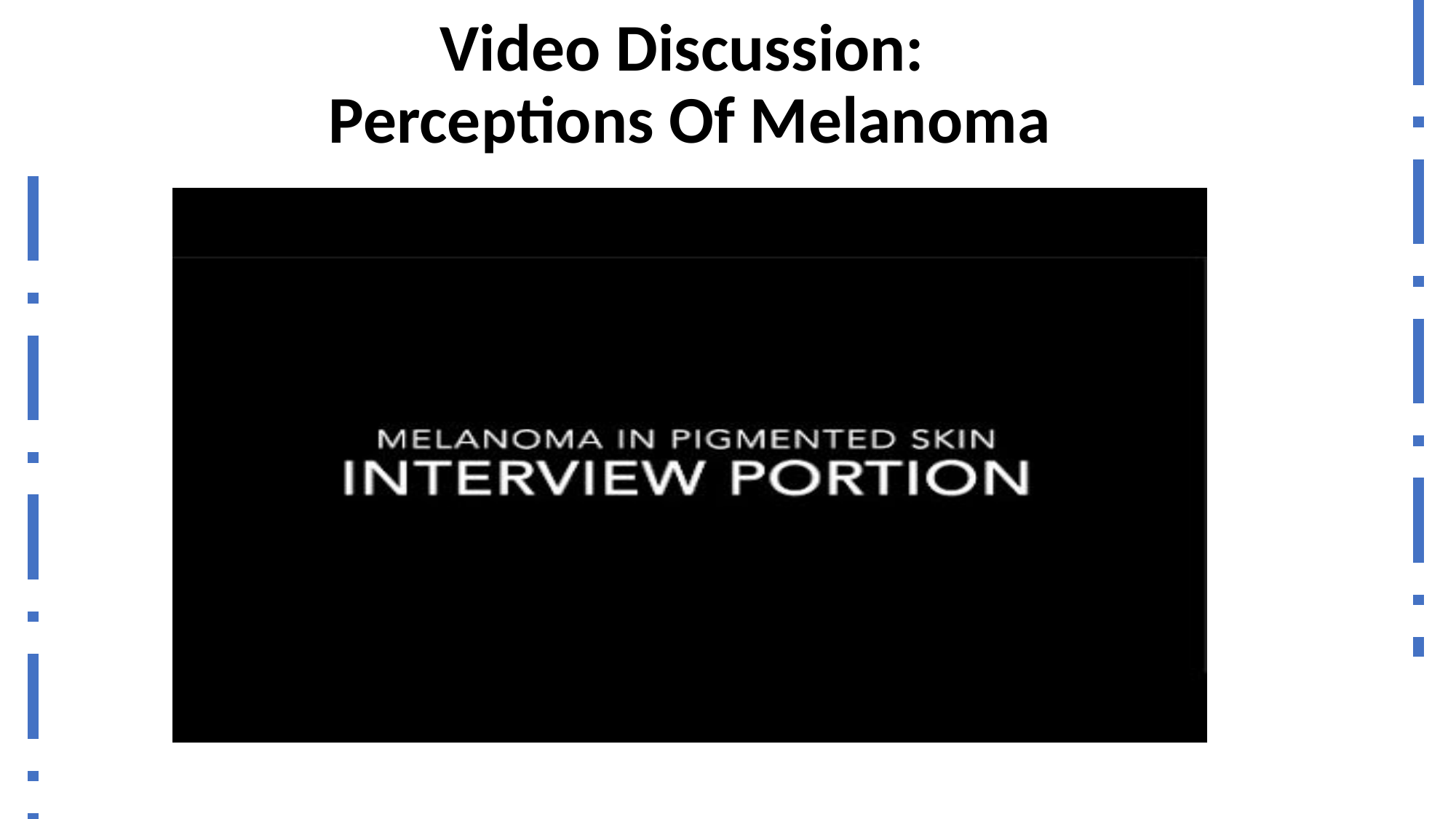

Video Discussion:
Perceptions Of Melanoma

## Slide 15
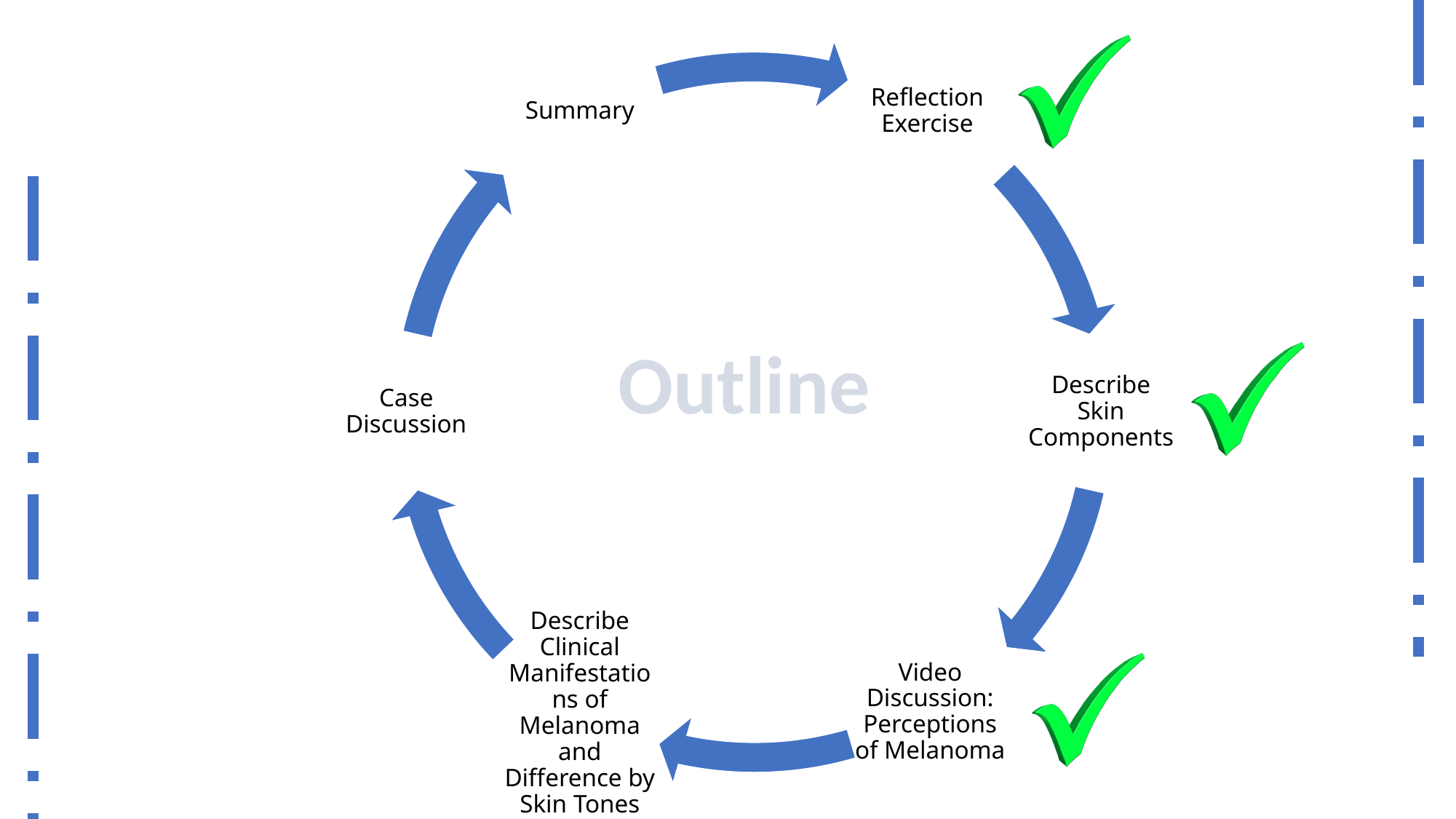

Summary
Reflection Exercise
Case Discussion
Describe Skin Components
Video Discussion: Perceptions of Melanoma
Describe Clinical Manifestations of Melanoma and Difference by Skin Tones
Outline

## Slide 16
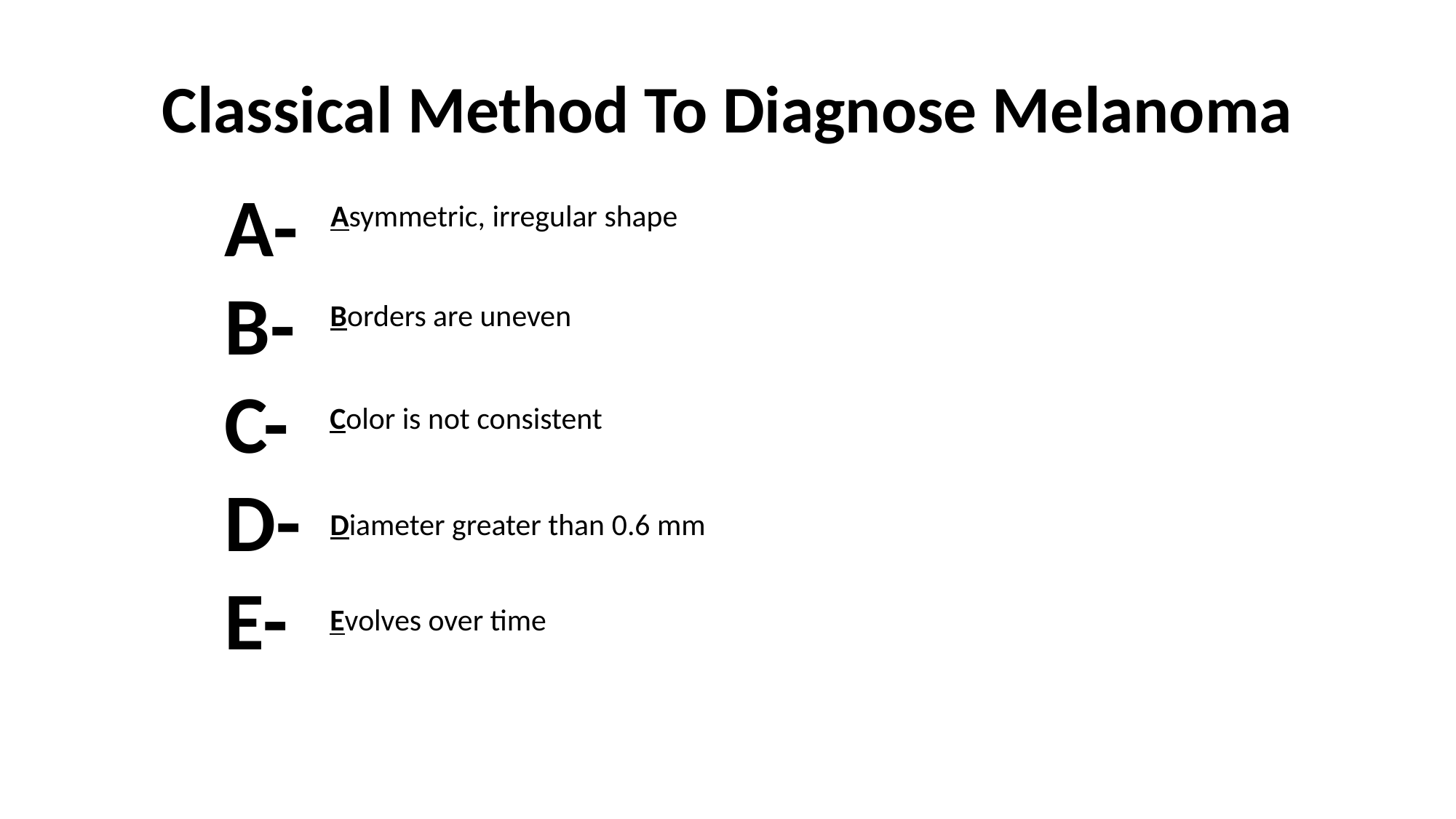

Classical Method To Diagnose Melanoma
A-
B-
C-
D-
E-
Asymmetric, irregular shape
Borders are uneven
Color is not consistent
Diameter greater than 0.6 mm
Evolves over time

## Slide 17
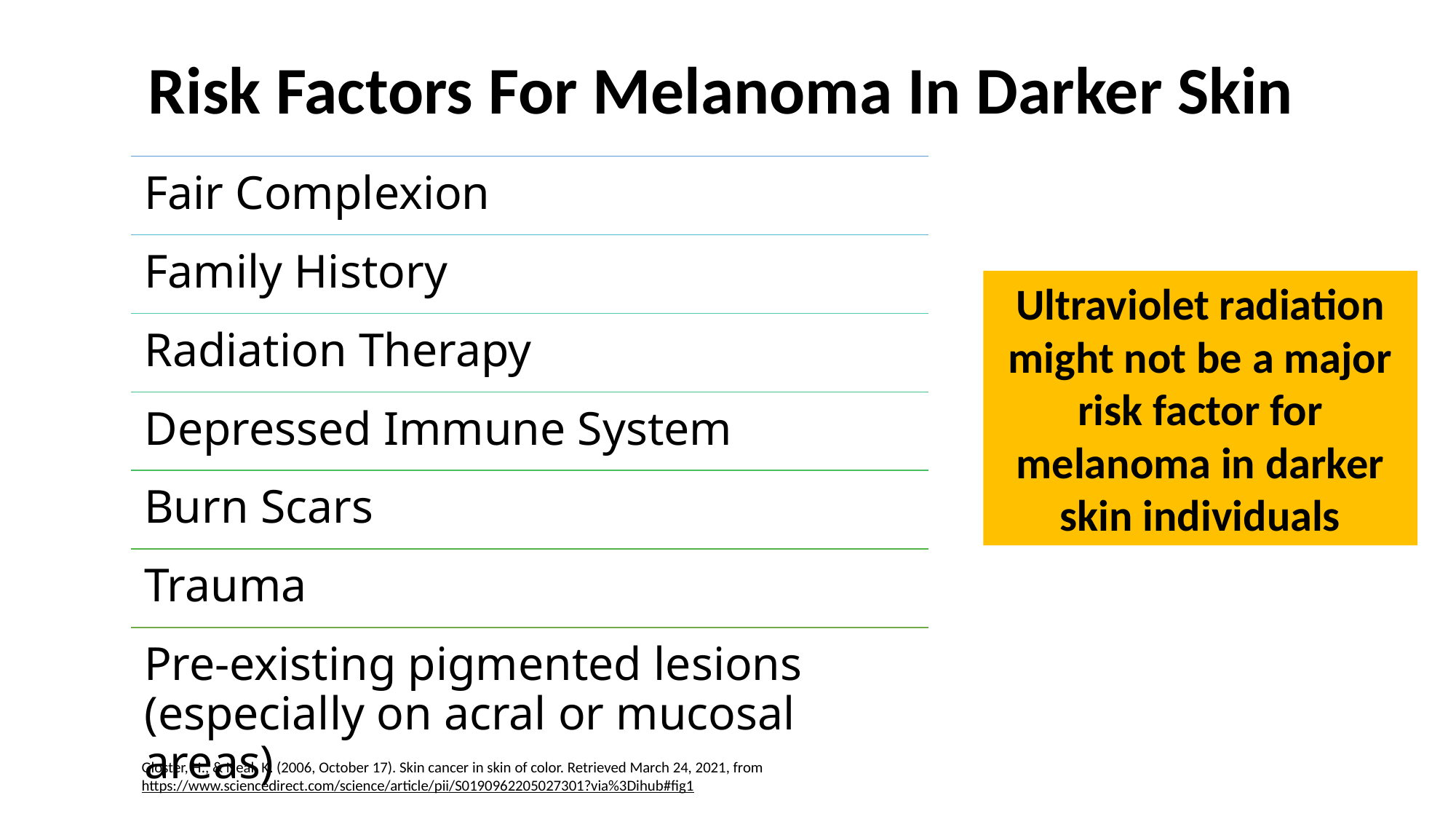

Risk Factors For Melanoma In Darker Skin
Fair Complexion
Family History
Radiation Therapy
Depressed Immune System
Burn Scars
Trauma
Pre-existing pigmented lesions (especially on acral or mucosal areas)
Ultraviolet radiation might not be a major risk factor for melanoma in darker skin individuals
Gloster, H., & Neal, K. (2006, October 17). Skin cancer in skin of color. Retrieved March 24, 2021, from https://www.sciencedirect.com/science/article/pii/S0190962205027301?via%3Dihub#fig1

## Slide 18
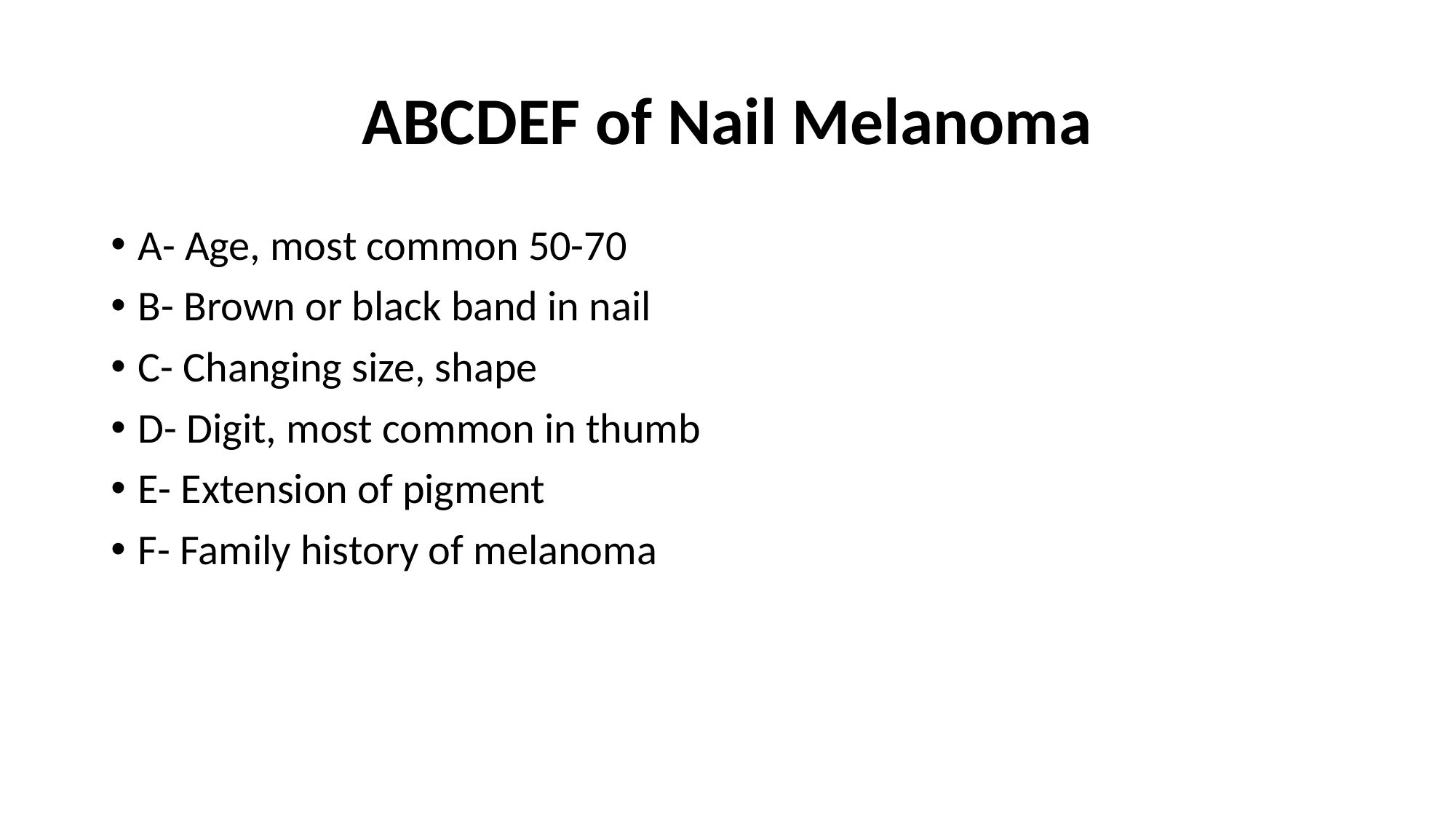

# ABCDEF of Nail Melanoma
A- Age, most common 50-70
B- Brown or black band in nail
C- Changing size, shape
D- Digit, most common in thumb
E- Extension of pigment
F- Family history of melanoma

## Slide 19
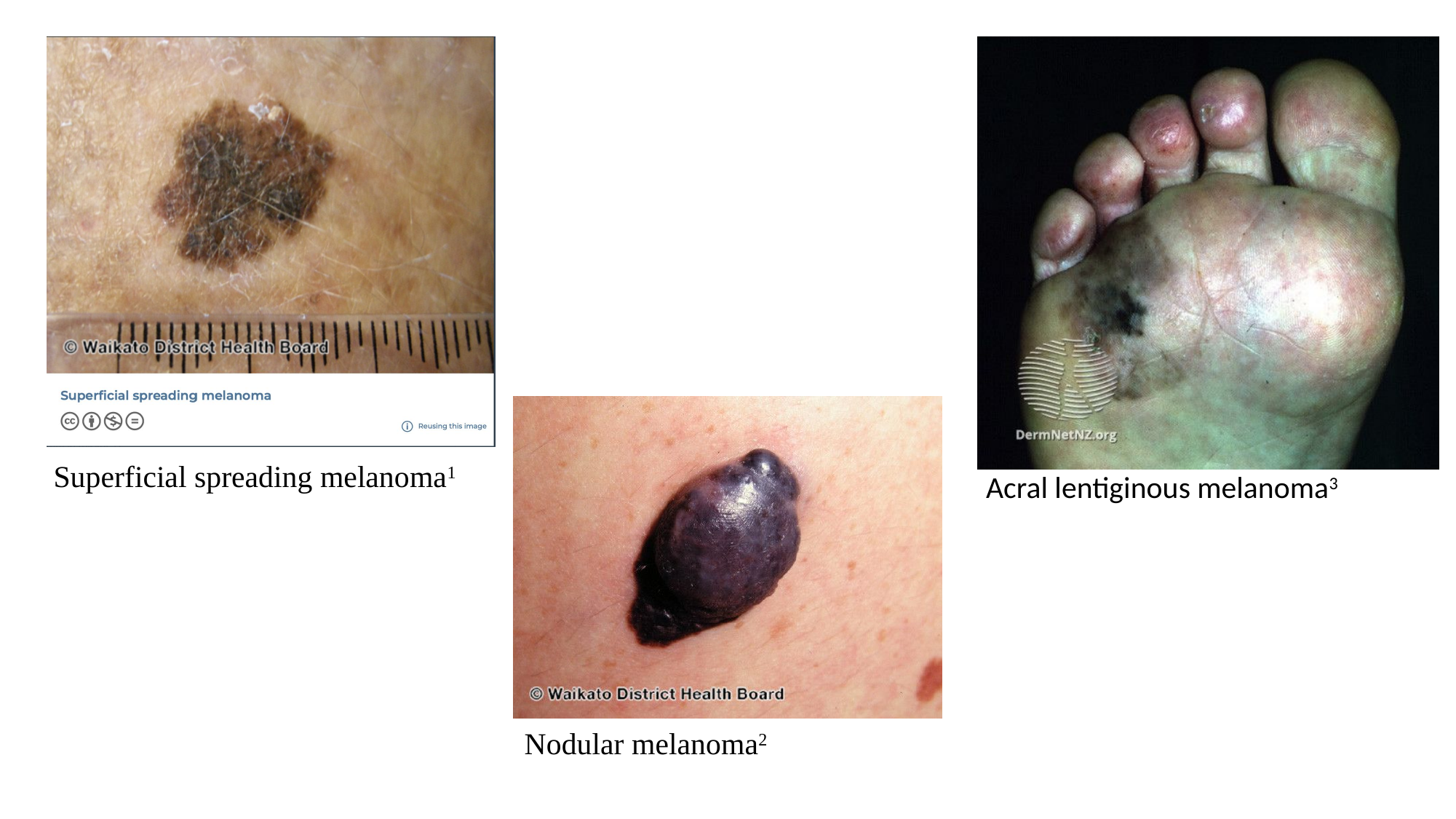

Superficial spreading melanoma1
Acral lentiginous melanoma3
Nodular melanoma2

## Slide 20
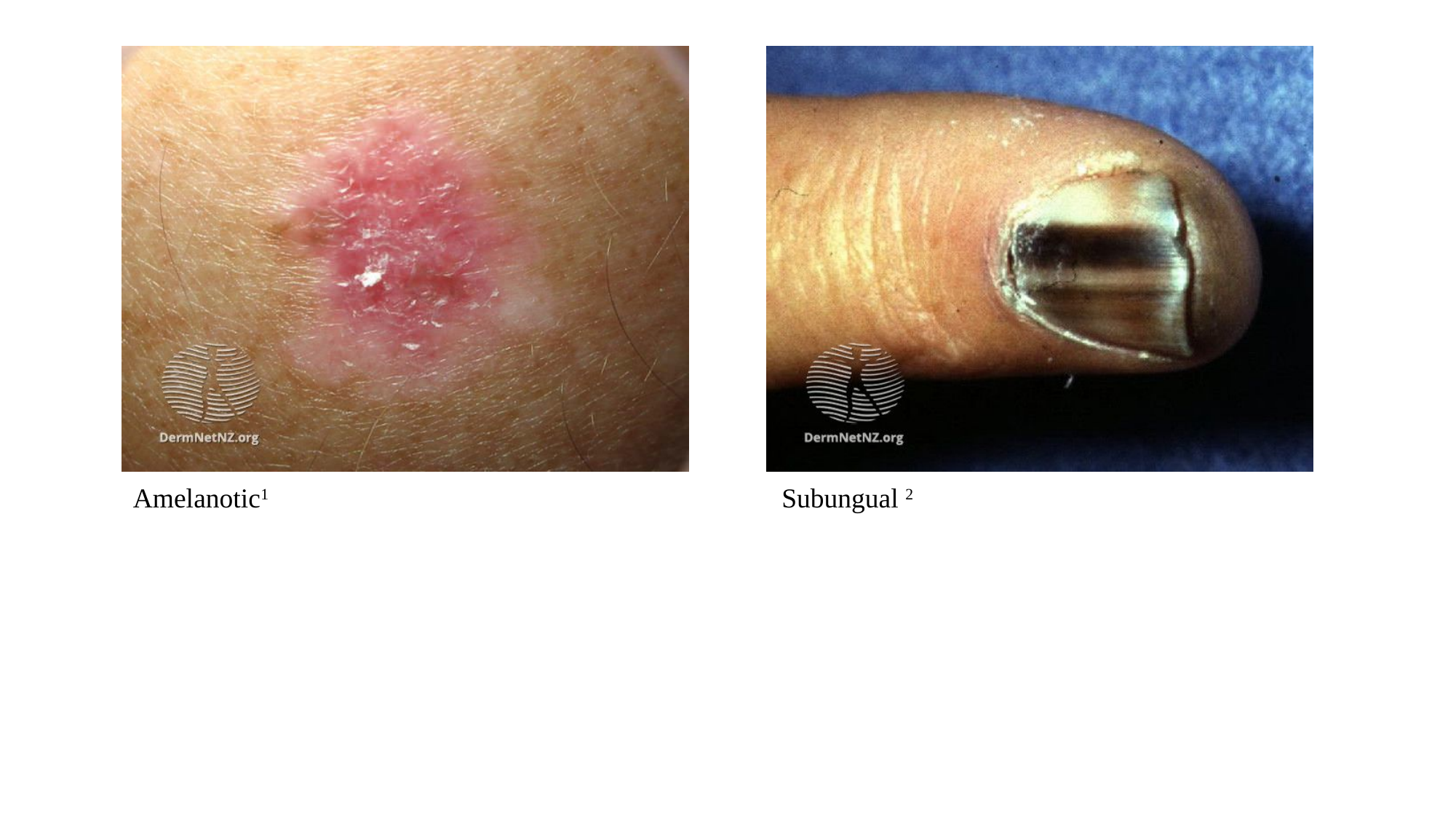

Amelanotic1
Subungual 2

## Slide 21
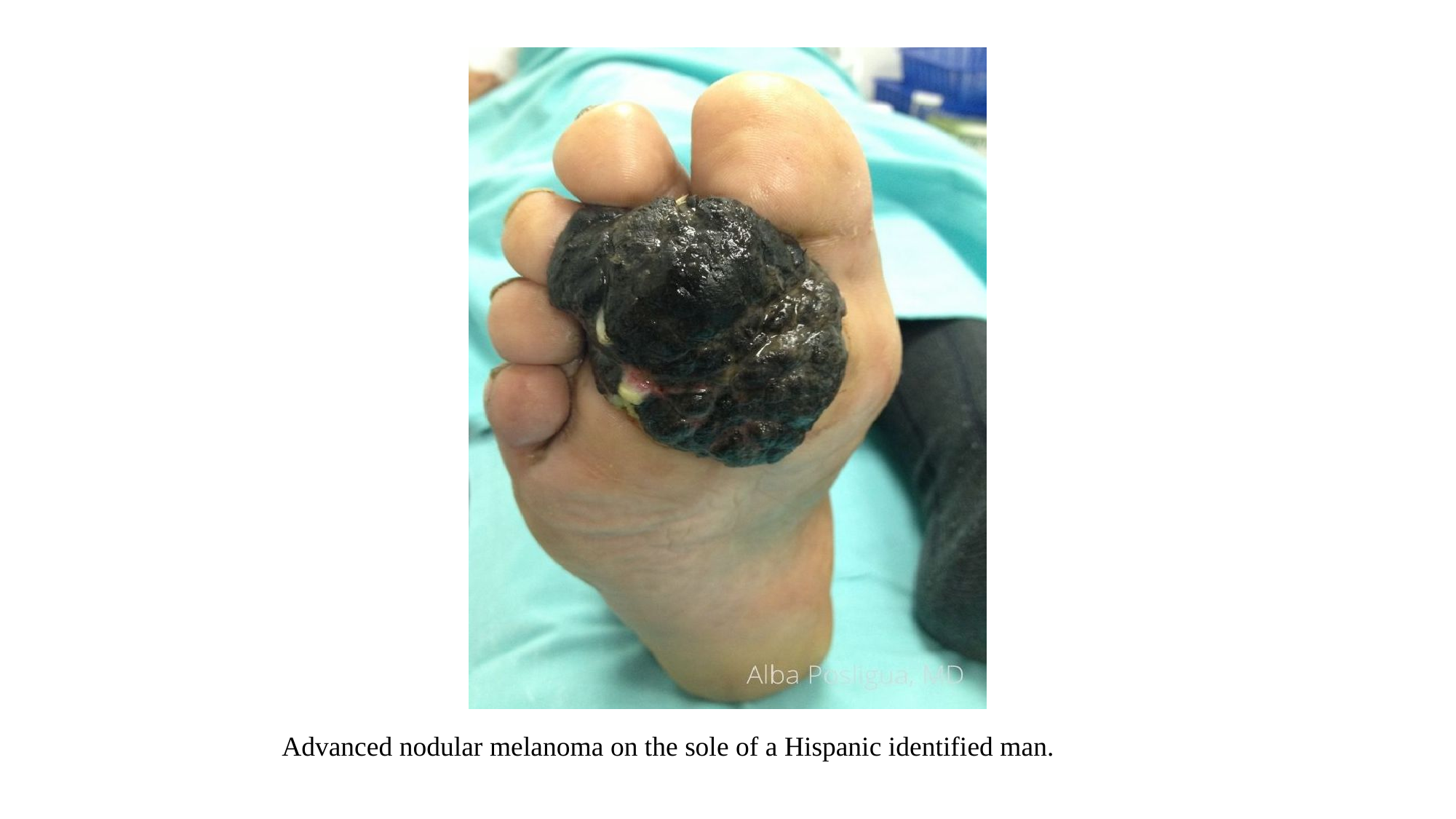

Advanced nodular melanoma on the sole of a Hispanic identified man.

## Slide 22
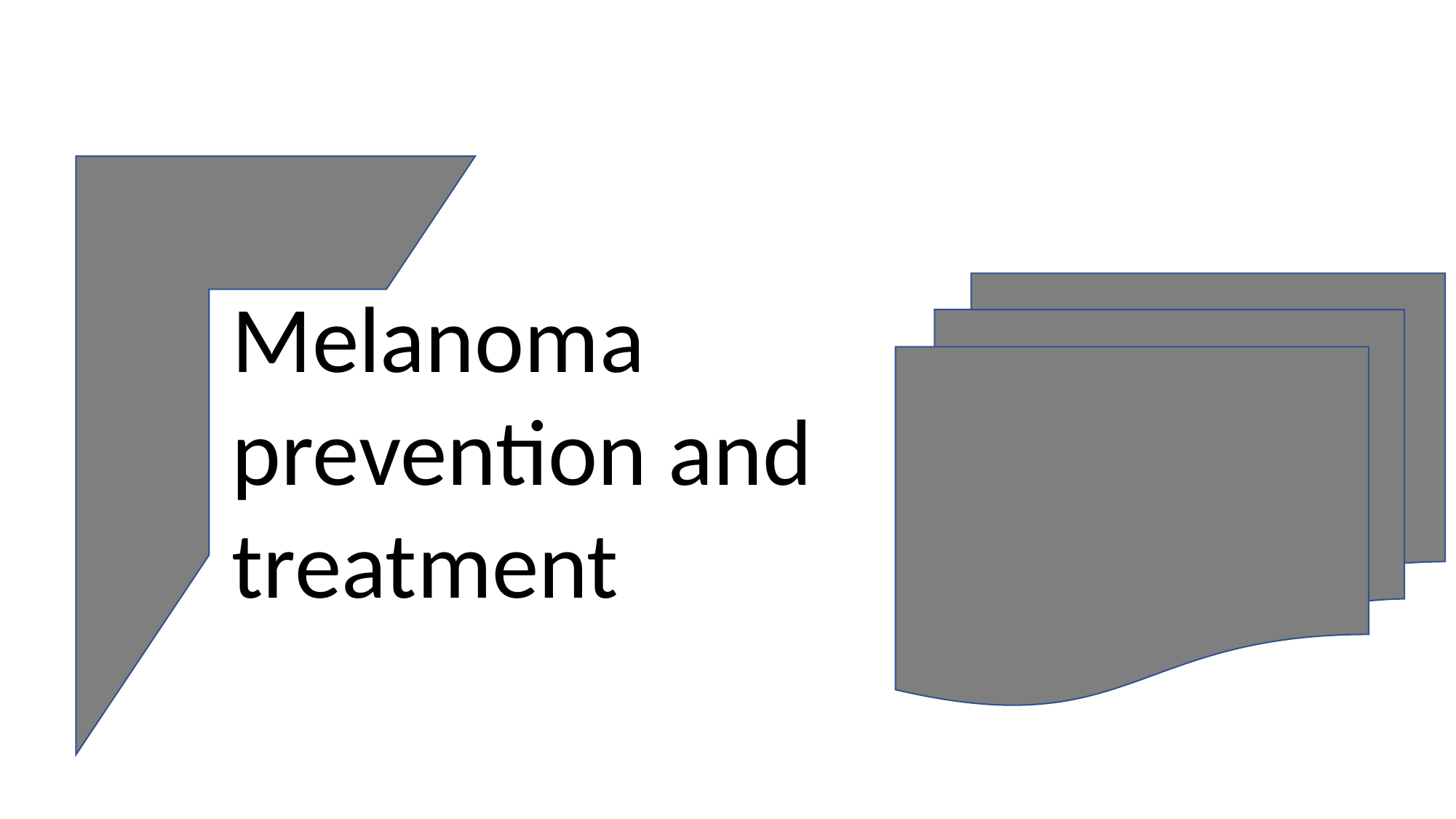

Melanoma prevention and treatment

## Slide 23
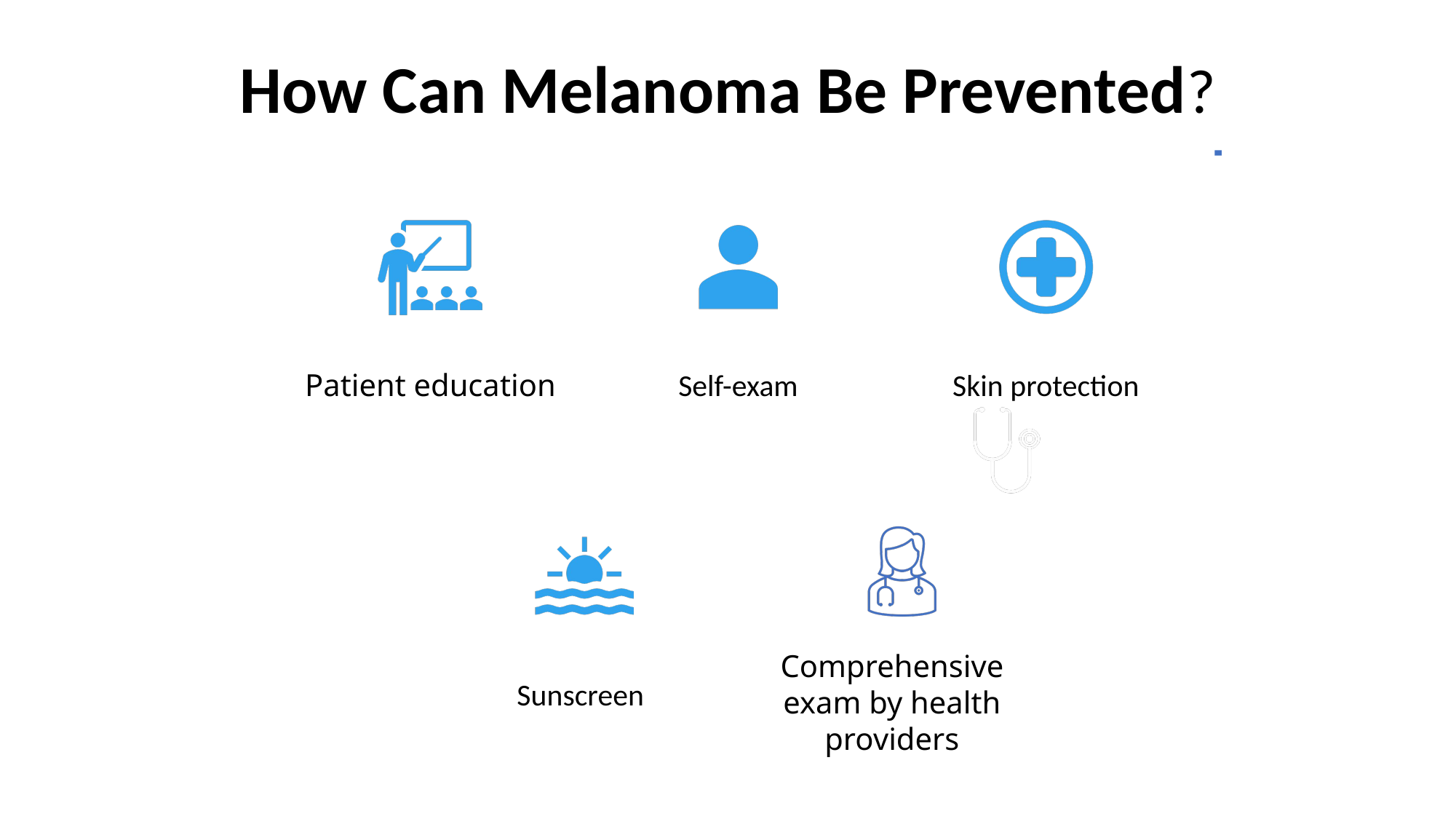

How Can Melanoma Be Prevented?
Patient education
Self-exam
Skin protection
Comprehensive exam by health providers
Sunscreen

## Slide 24
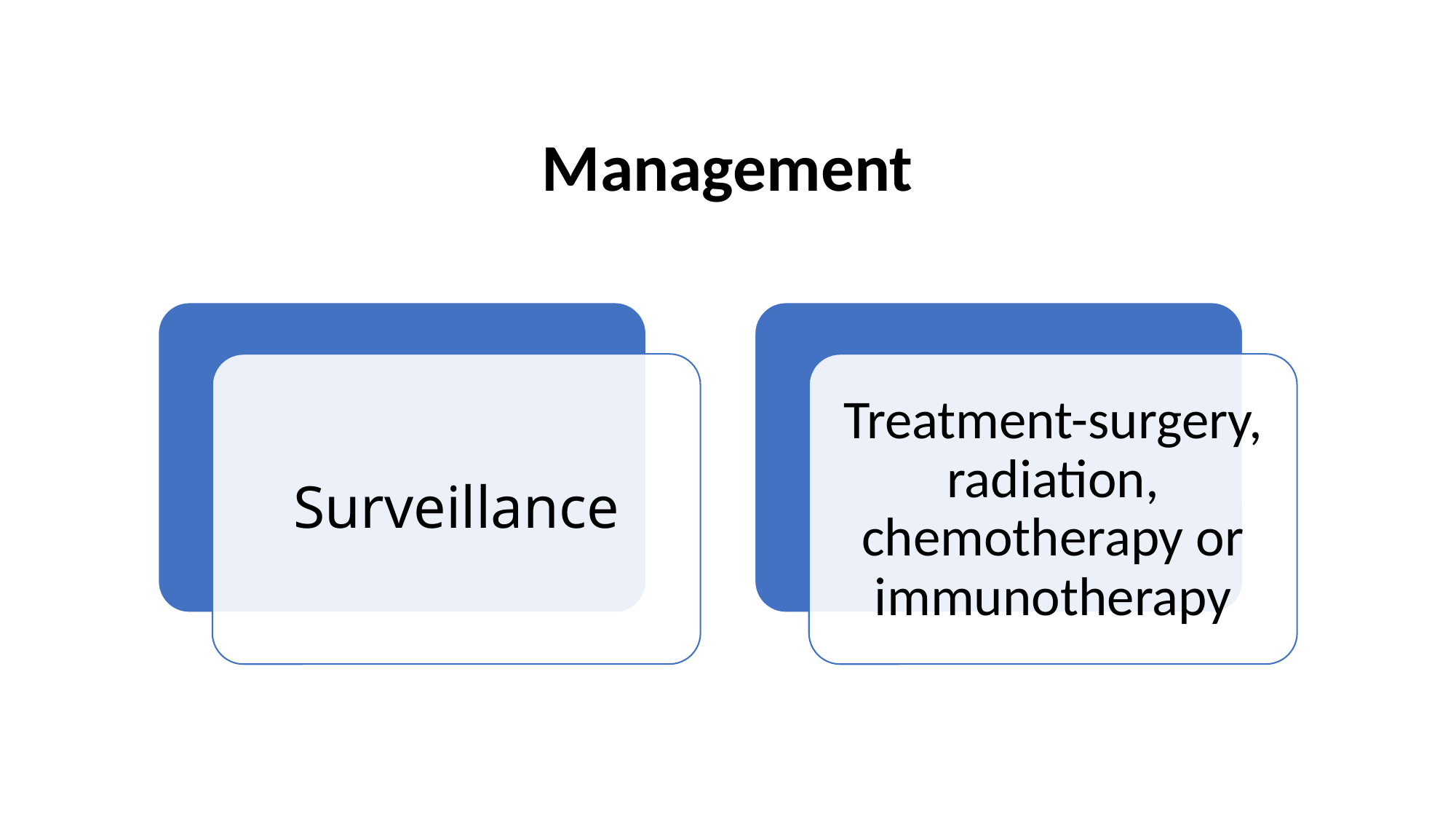

Management
Surveillance
Treatment-surgery, radiation, chemotherapy or immunotherapy

## Slide 25
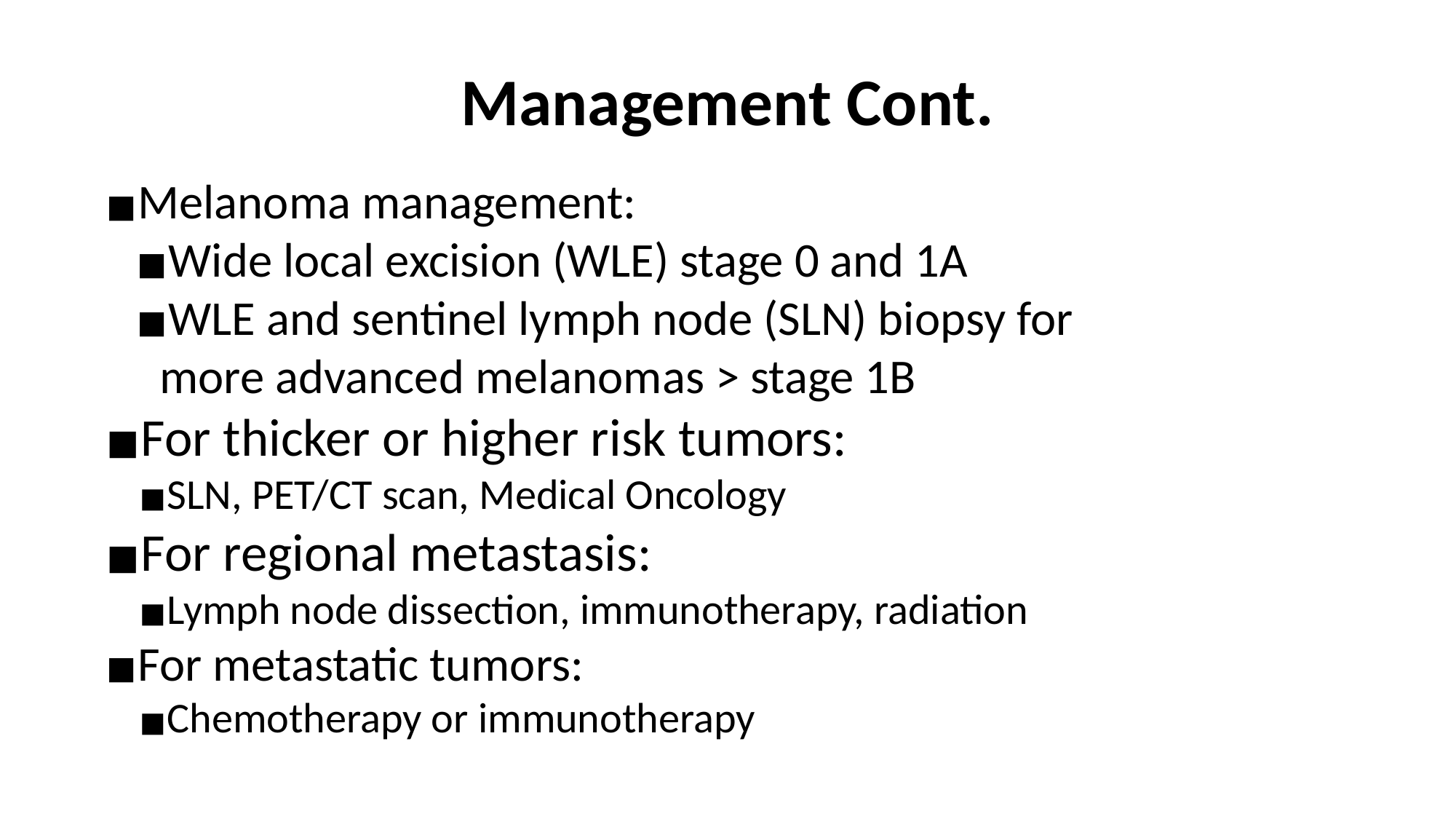

Management Cont.
Melanoma management:
Wide local excision (WLE) stage 0 and 1A
WLE and sentinel lymph node (SLN) biopsy for more advanced melanomas > stage 1B
For thicker or higher risk tumors:
SLN, PET/CT scan, Medical Oncology
For regional metastasis:
Lymph node dissection, immunotherapy, radiation
For metastatic tumors:
Chemotherapy or immunotherapy

## Slide 26
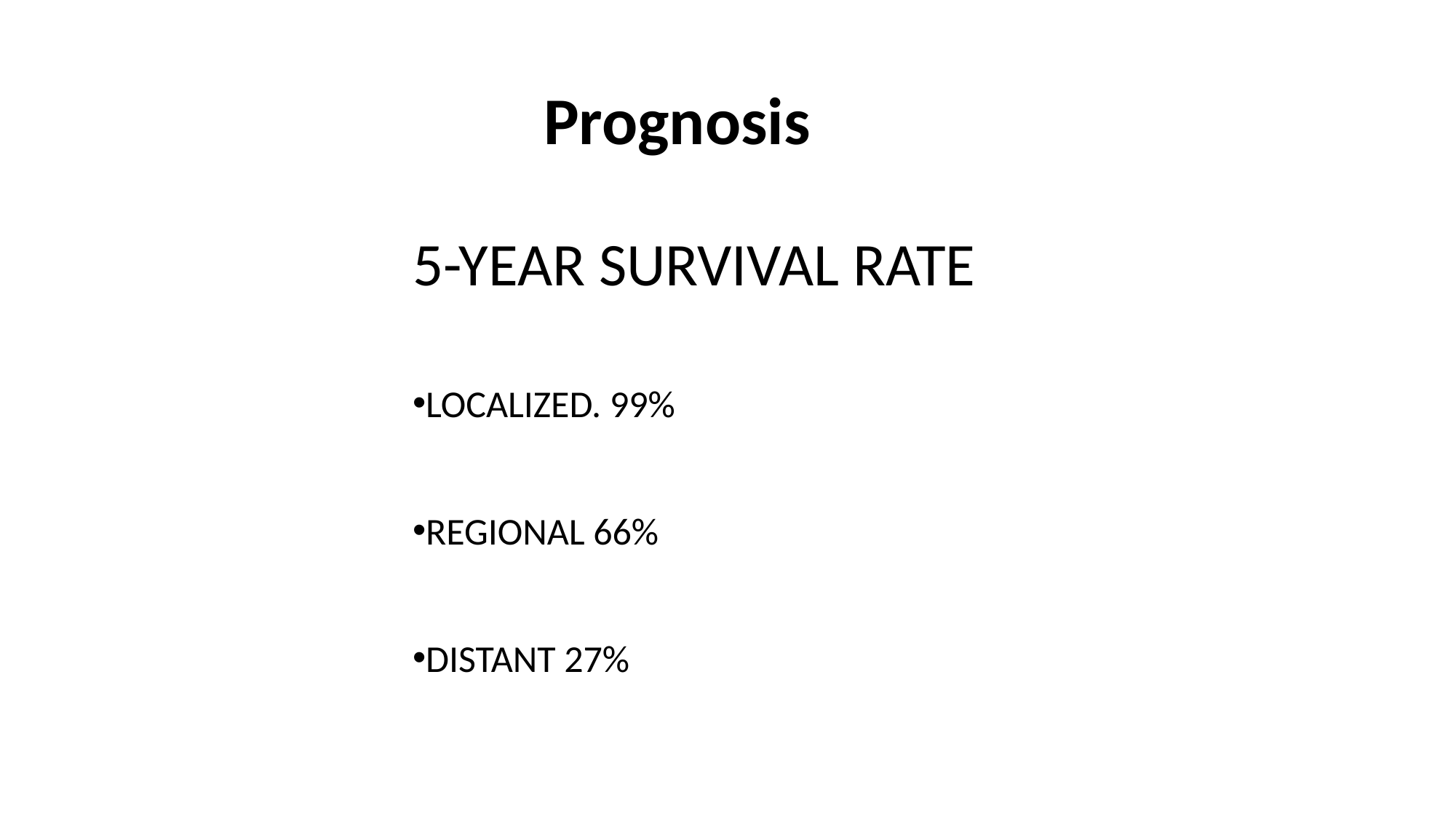

Prognosis
5-YEAR SURVIVAL RATE
LOCALIZED. 99%
REGIONAL 66%
DISTANT 27%

## Slide 27
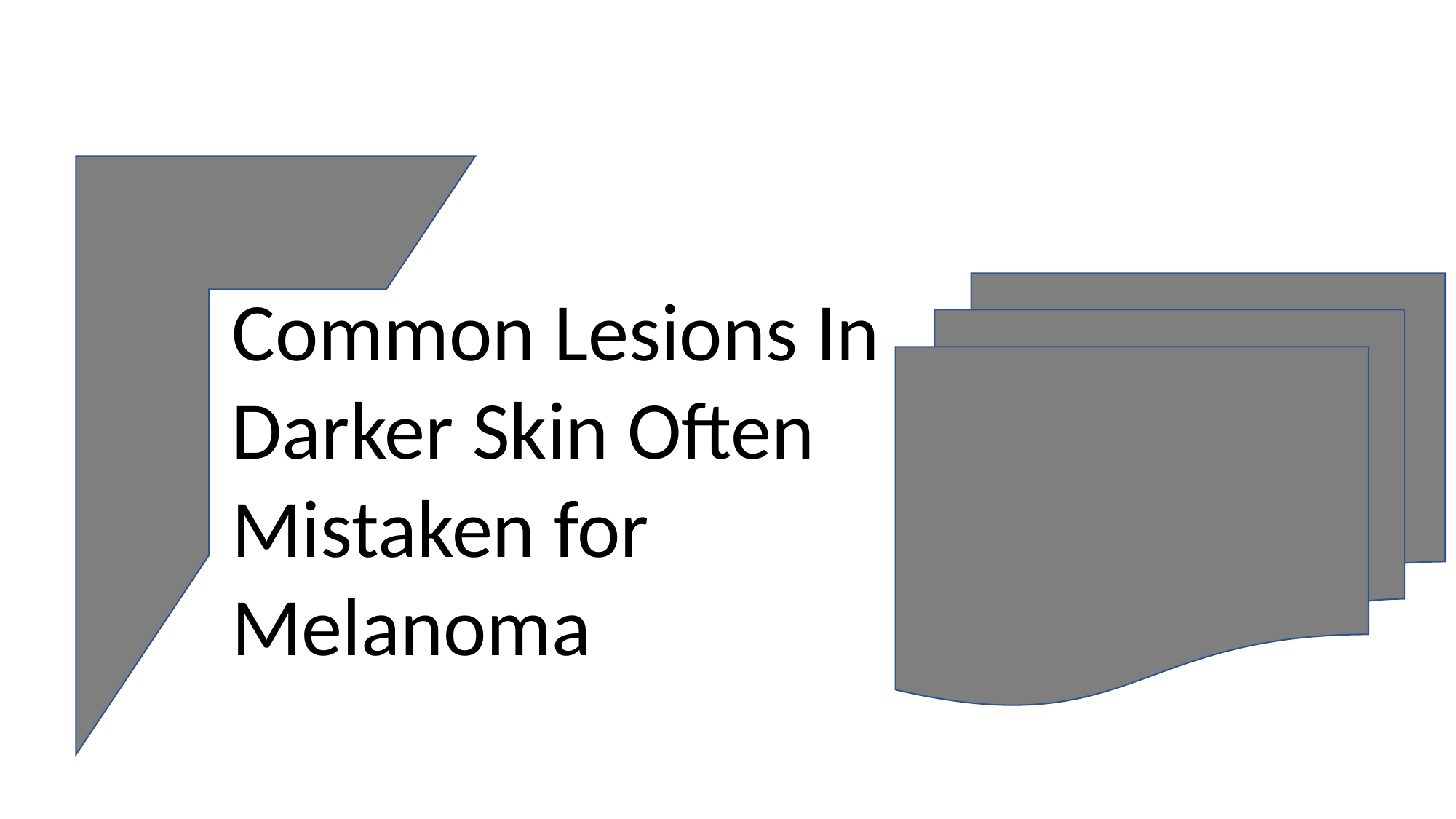

Common Lesions In Darker Skin Often Mistaken for Melanoma

## Slide 28
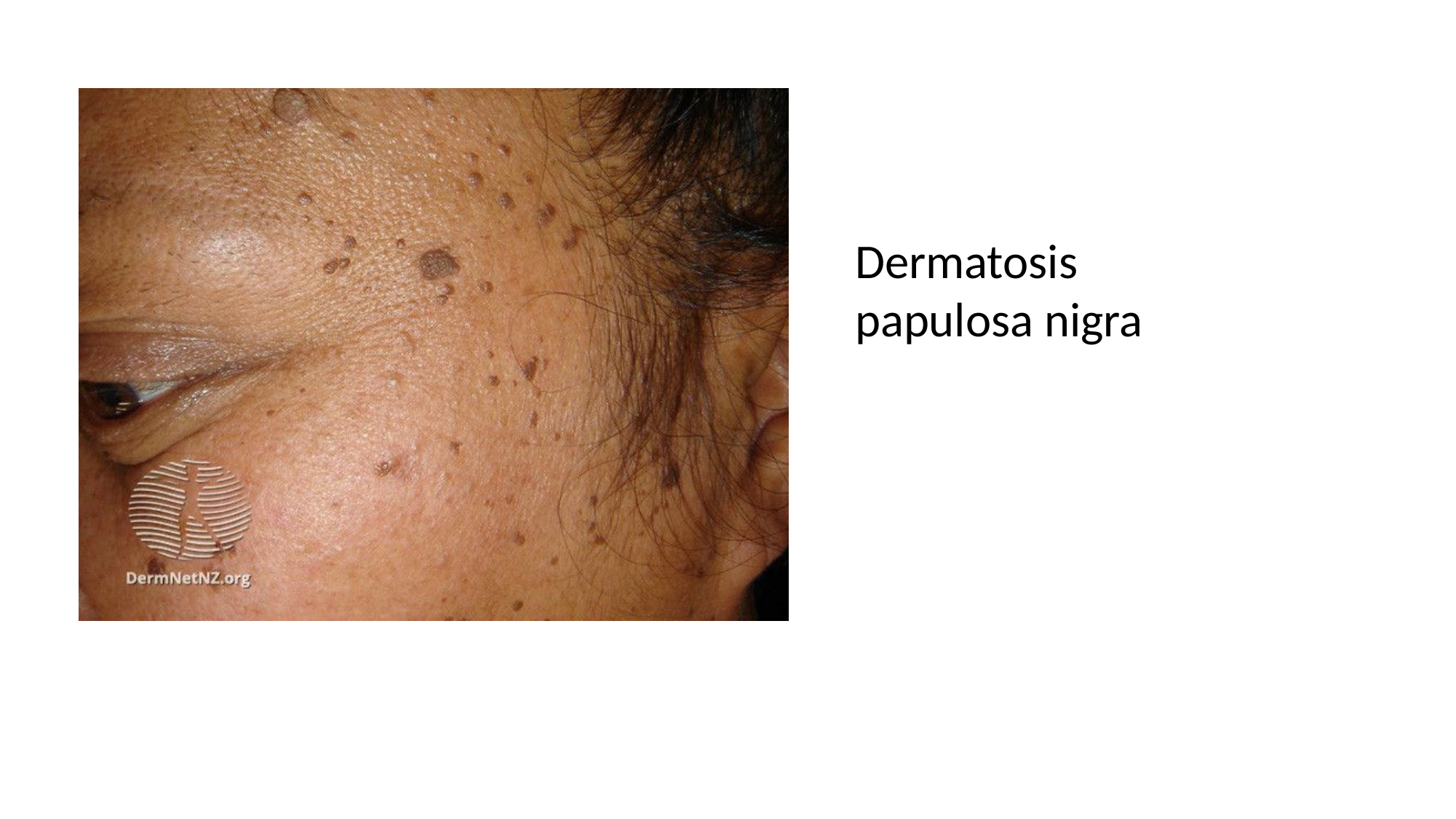

Dermatosis papulosa nigra

## Slide 29
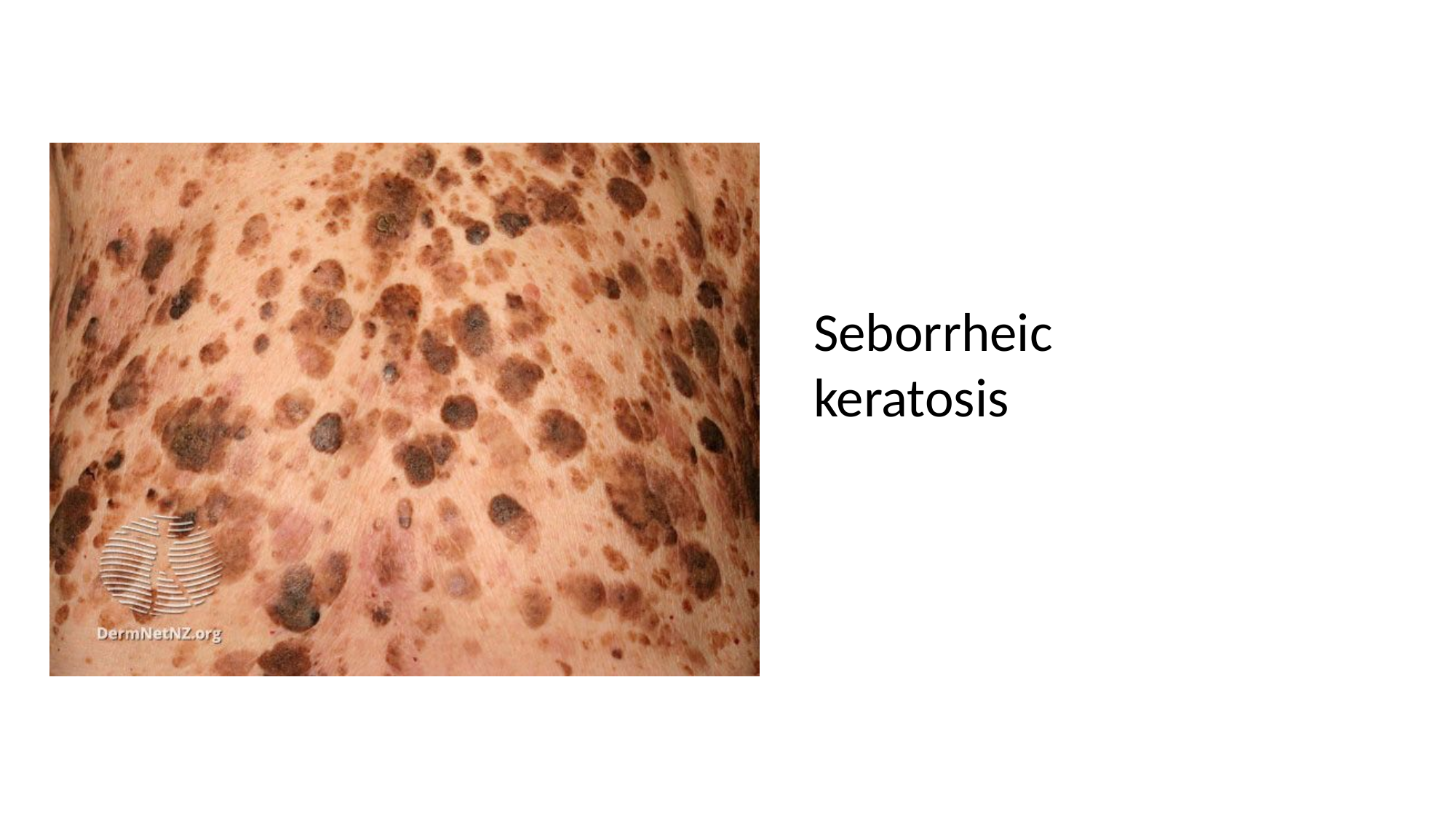

Seborrheic keratosis

## Slide 30
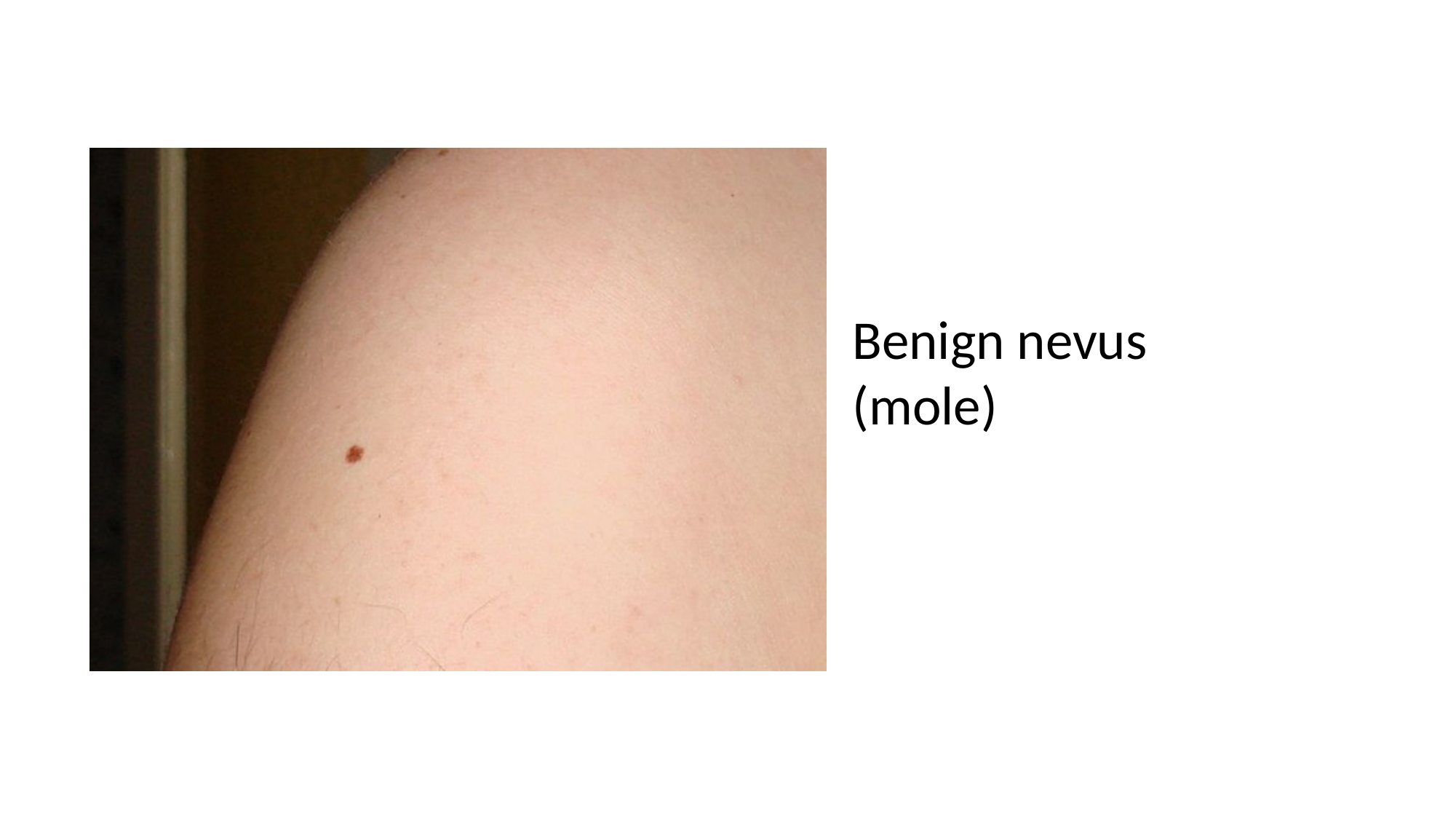

Benign nevus (mole)

## Slide 31
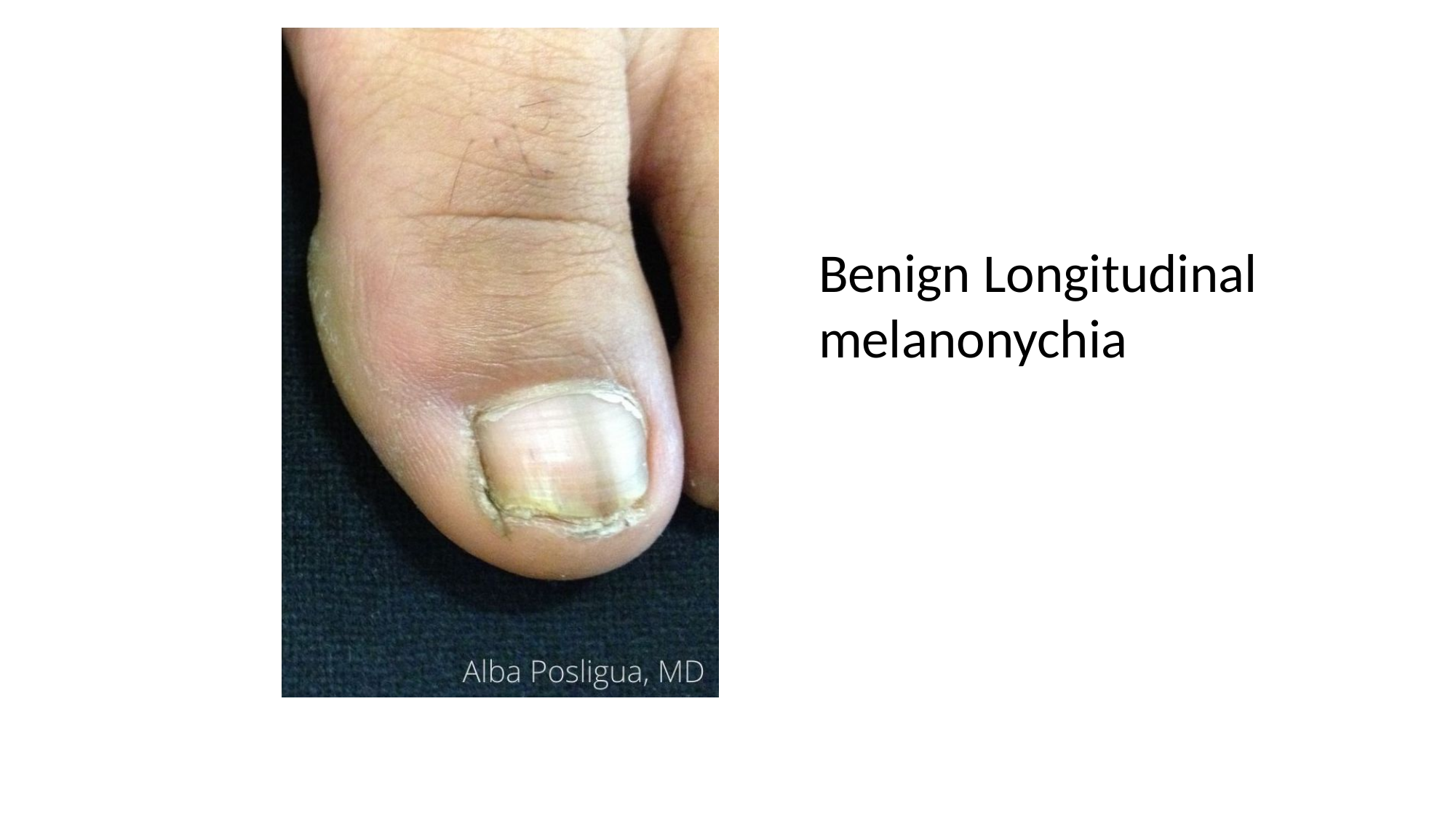

Benign Longitudinal melanonychia

## Slide 32
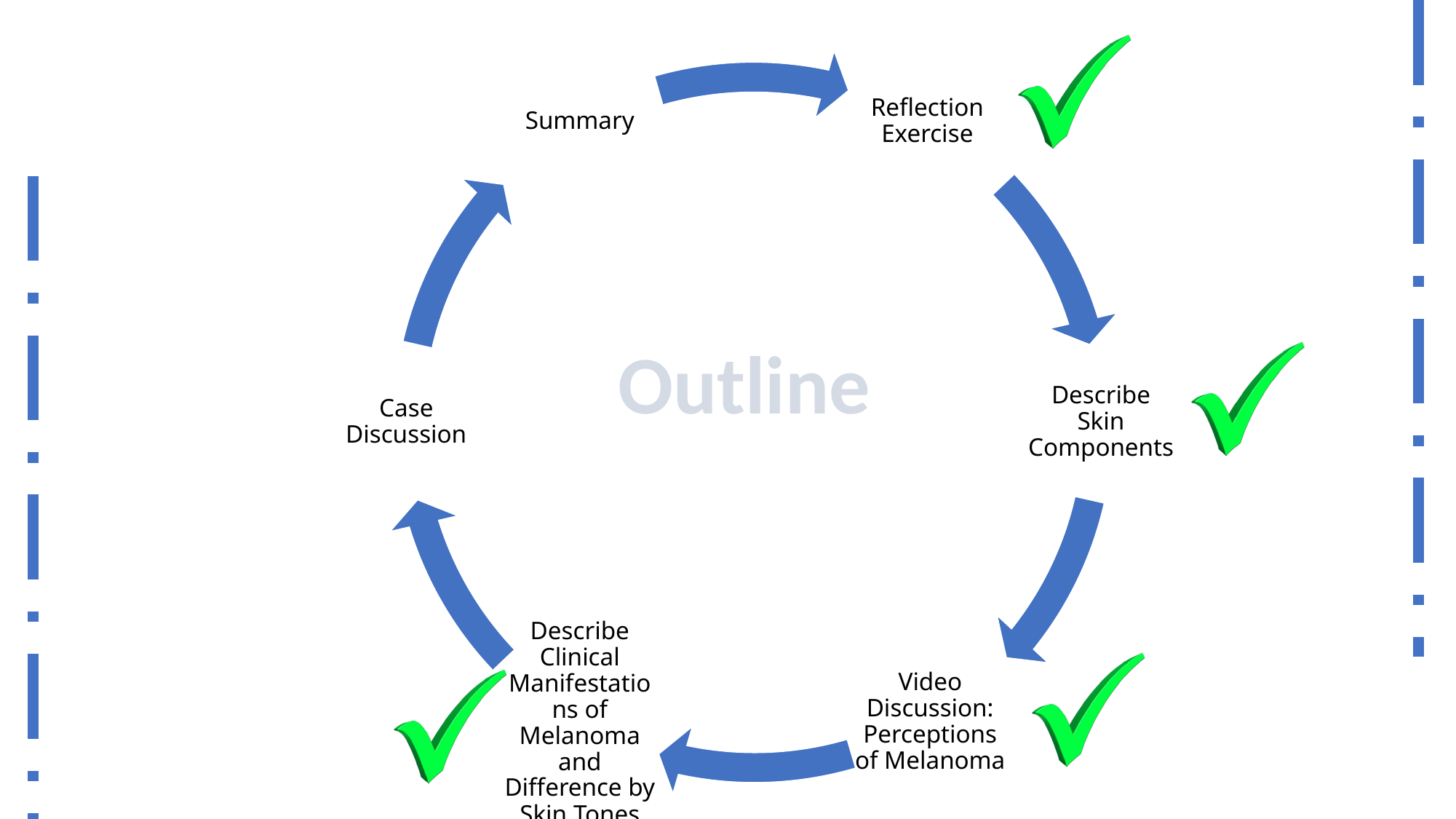

Summary
Reflection Exercise
Case Discussion
Describe Skin Components
Video Discussion: Perceptions of Melanoma
Describe Clinical Manifestations of Melanoma and Difference by Skin Tones
Outline

## Slide 33
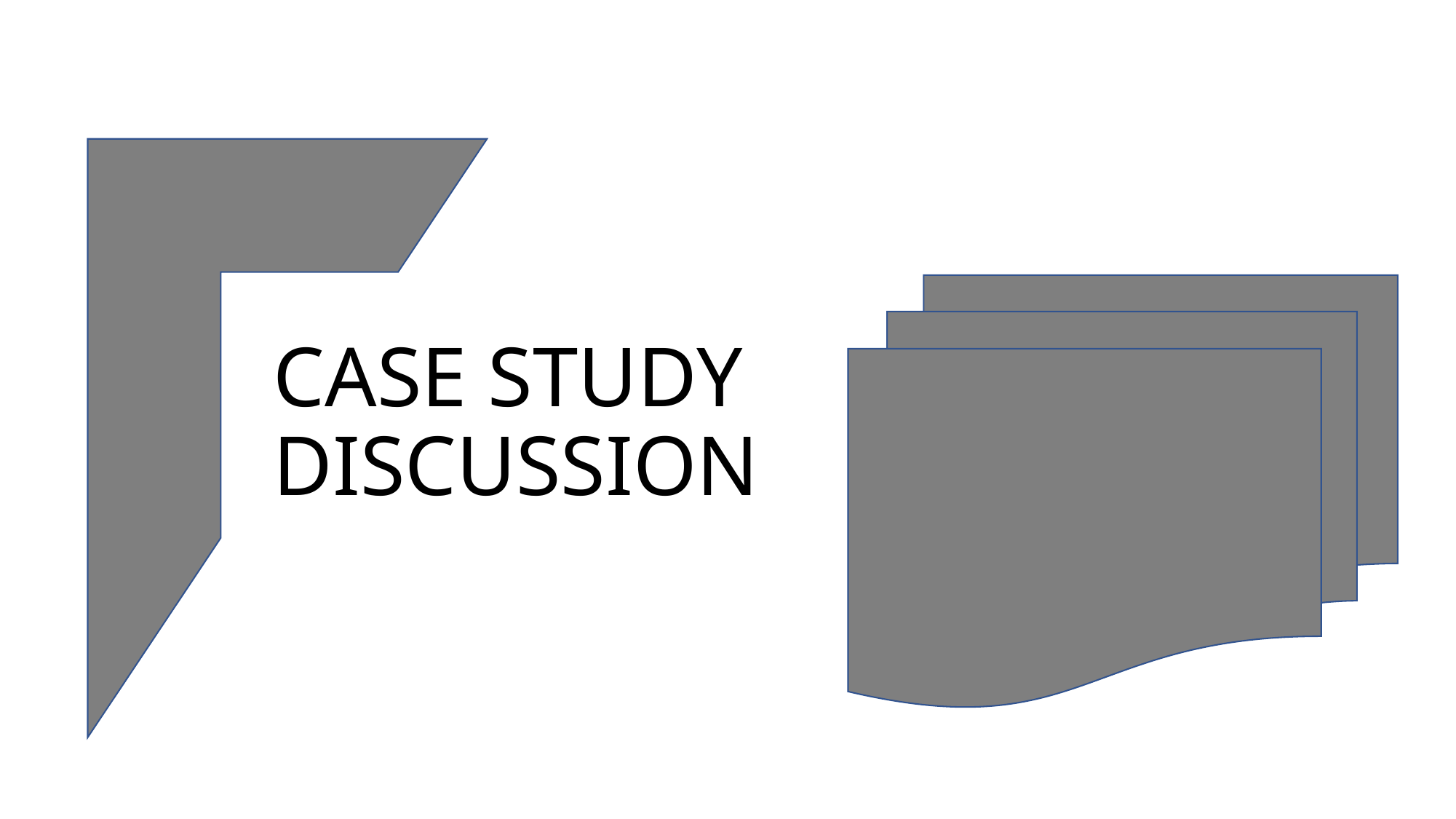

# CASE STUDY DISCUSSION

## Slide 34
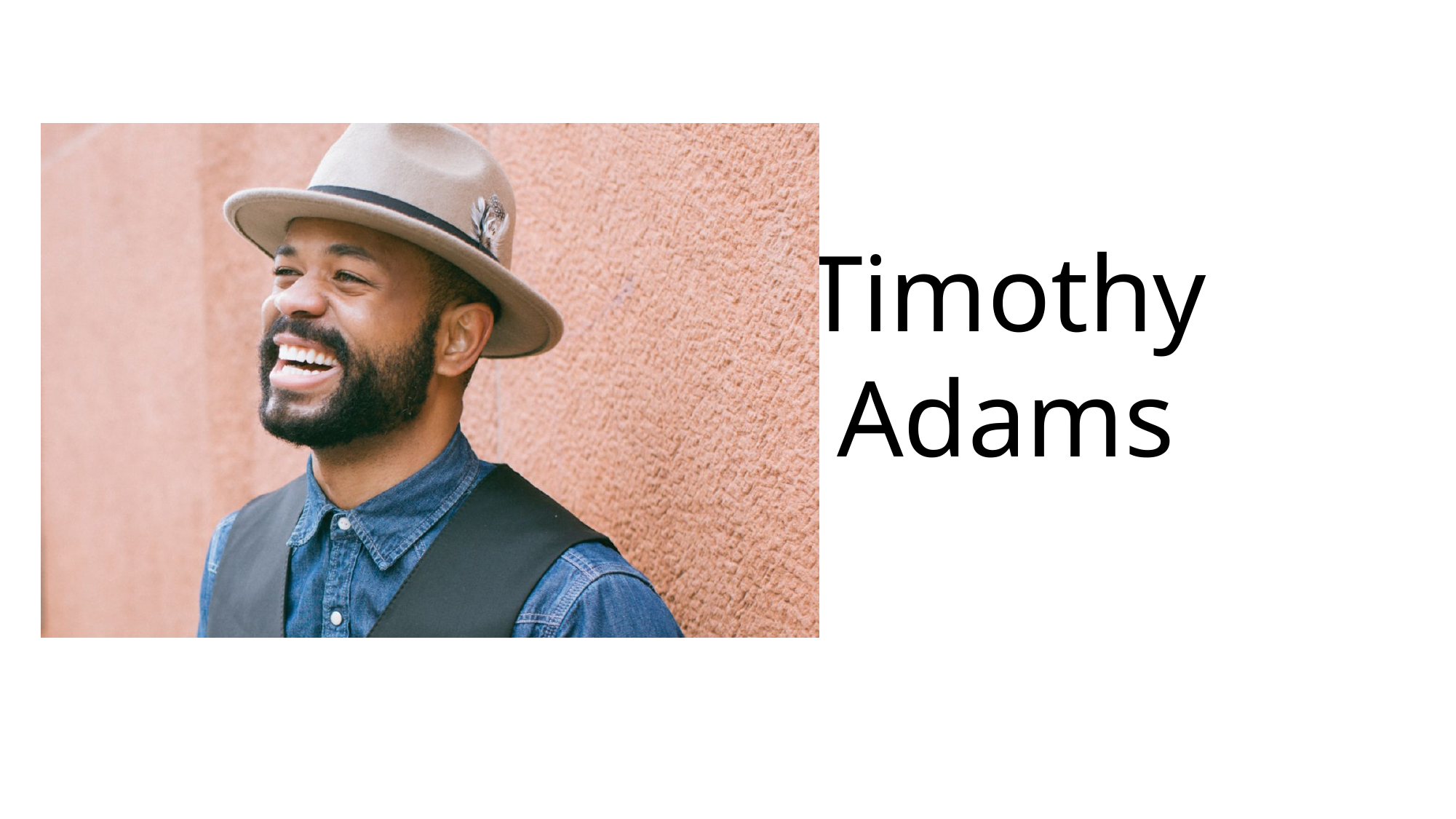

Timothy Adams

## Slide 35
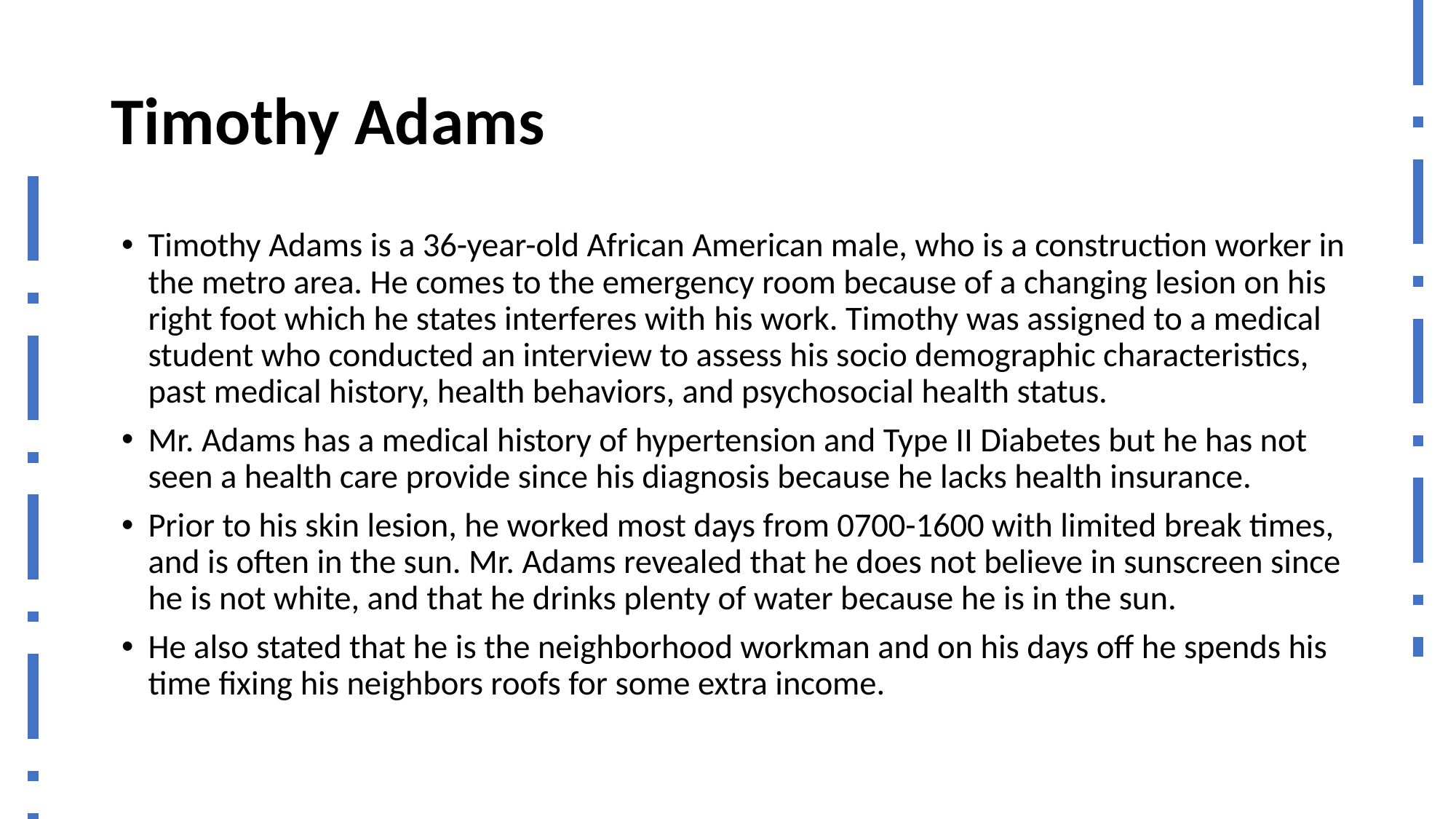

# Timothy Adams
Timothy Adams is a 36-year-old African American male, who is a construction worker in the metro area. He comes to the emergency room because of a changing lesion on his right foot which he states interferes with his work. Timothy was assigned to a medical student who conducted an interview to assess his socio demographic characteristics, past medical history, health behaviors, and psychosocial health status.
Mr. Adams has a medical history of hypertension and Type II Diabetes but he has not seen a health care provide since his diagnosis because he lacks health insurance.
Prior to his skin lesion, he worked most days from 0700-1600 with limited break times, and is often in the sun. Mr. Adams revealed that he does not believe in sunscreen since he is not white, and that he drinks plenty of water because he is in the sun.
He also stated that he is the neighborhood workman and on his days off he spends his time fixing his neighbors roofs for some extra income.

## Slide 36
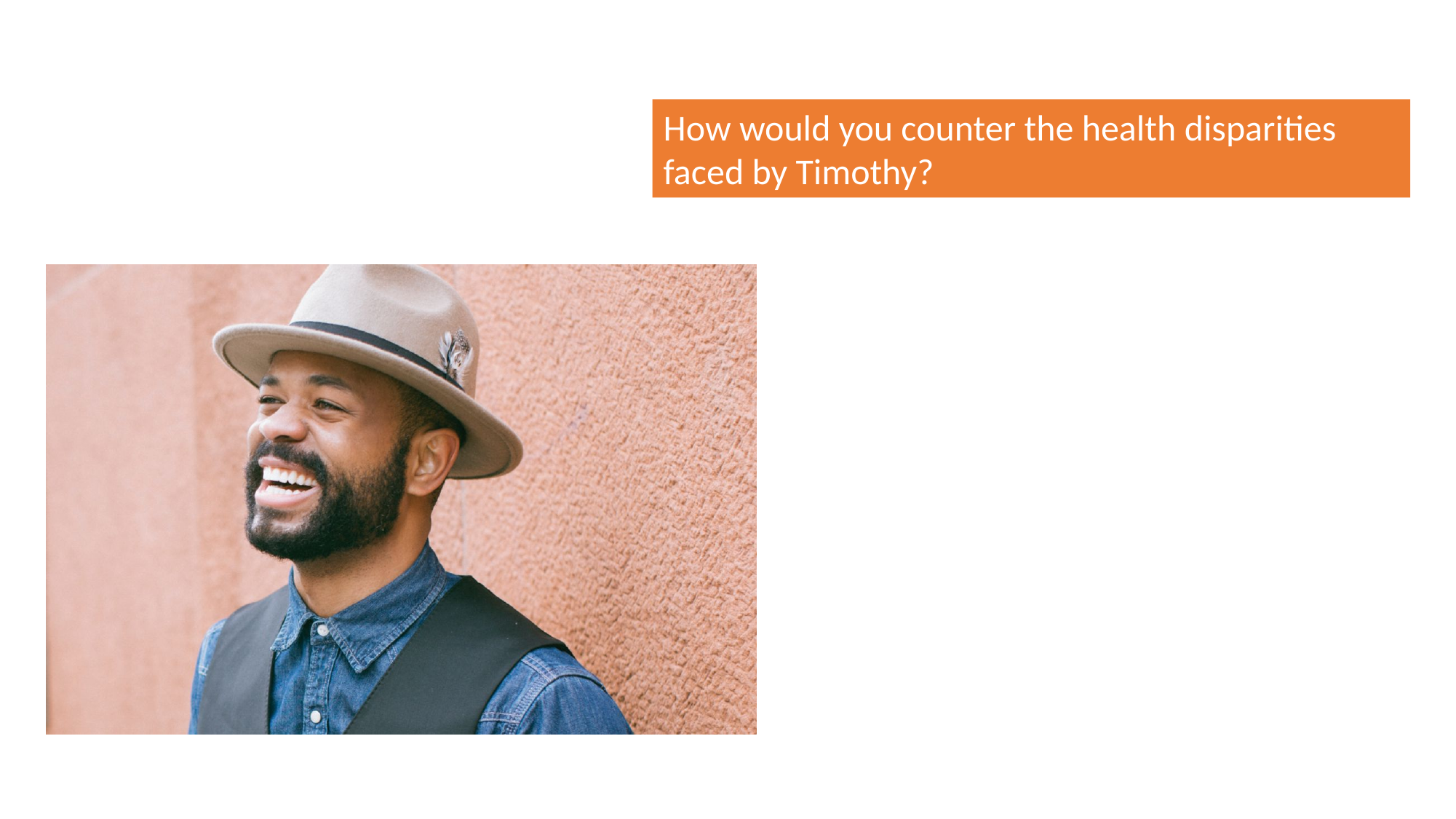

How would you counter the health disparities faced by Timothy?

## Slide 37
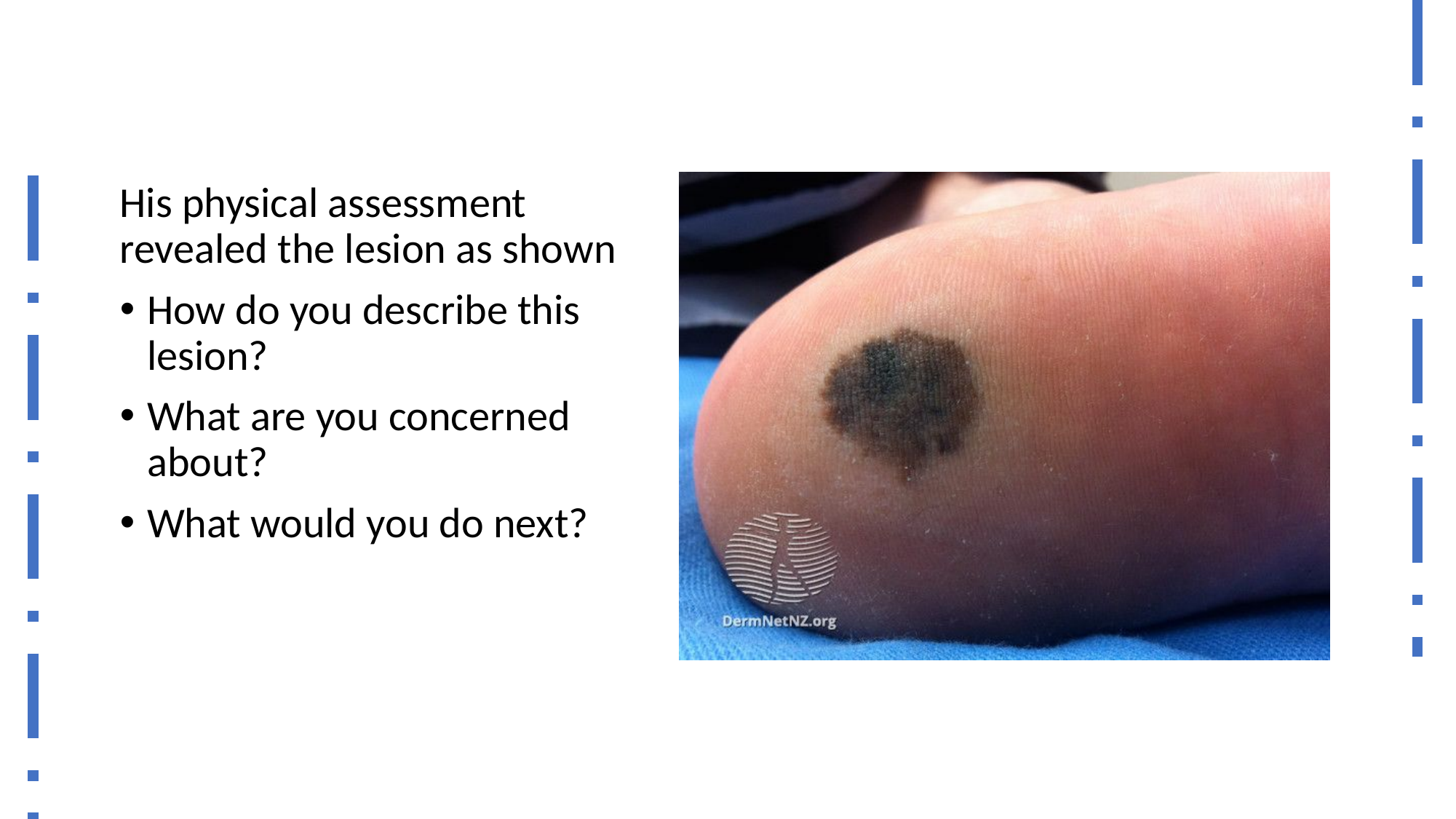

His physical assessment revealed the lesion as shown
How do you describe this lesion?
What are you concerned about?
What would you do next?

## Slide 38
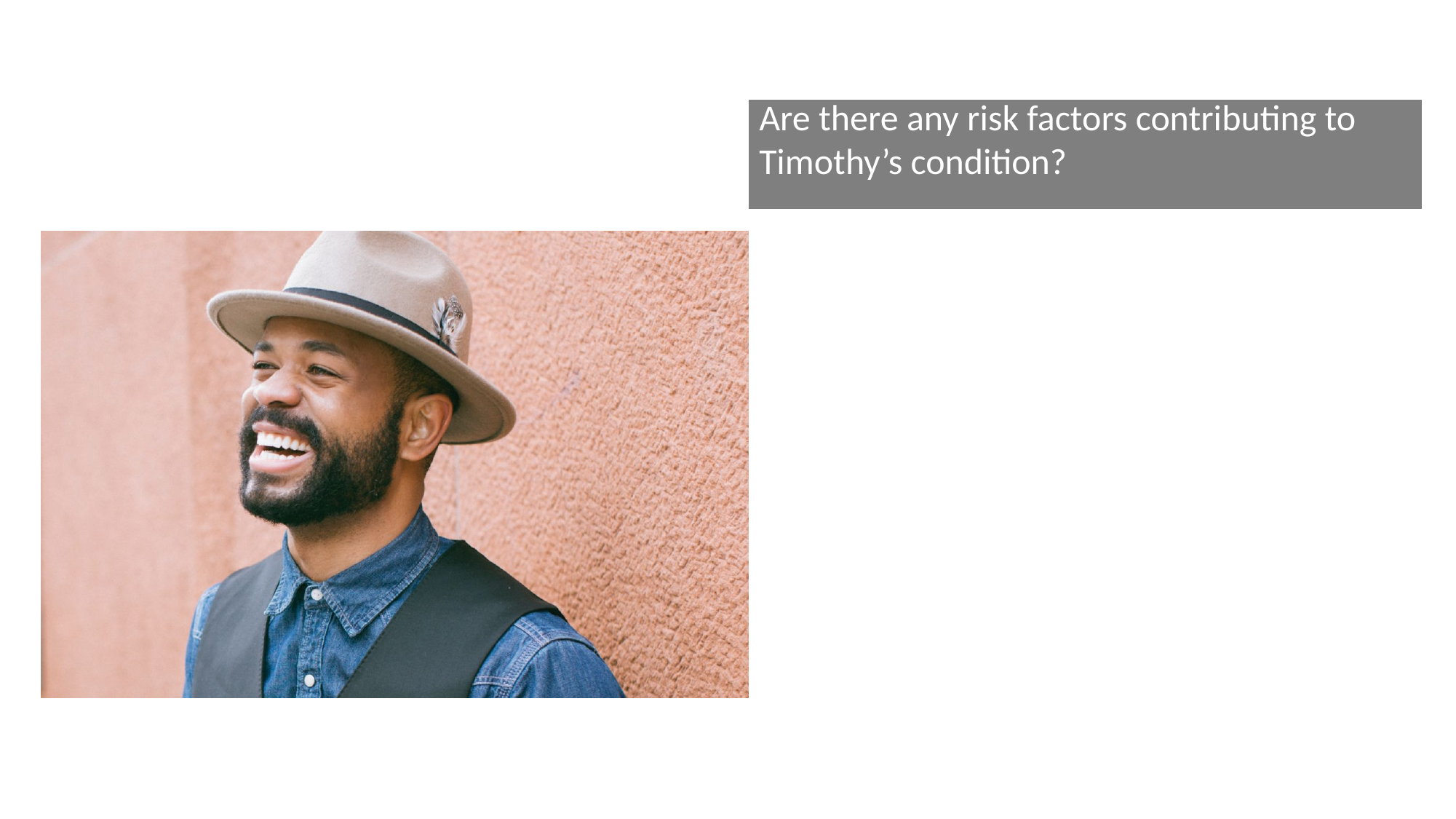

Are there any risk factors contributing to Timothy’s condition?

## Slide 39
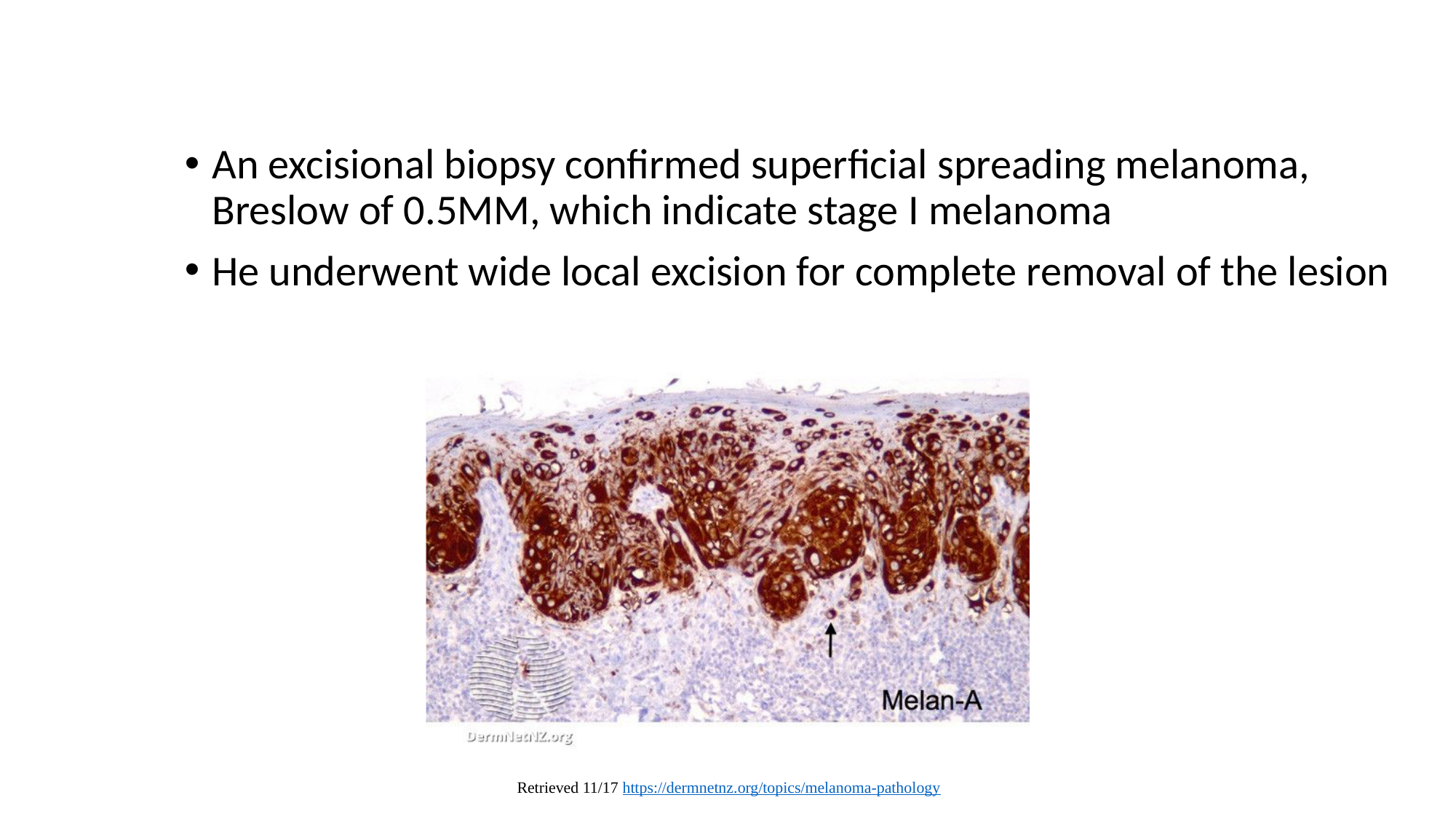

An excisional biopsy confirmed superficial spreading melanoma, Breslow of 0.5MM, which indicate stage I melanoma
He underwent wide local excision for complete removal of the lesion
Retrieved 11/17 https://dermnetnz.org/topics/melanoma-pathology

## Slide 40
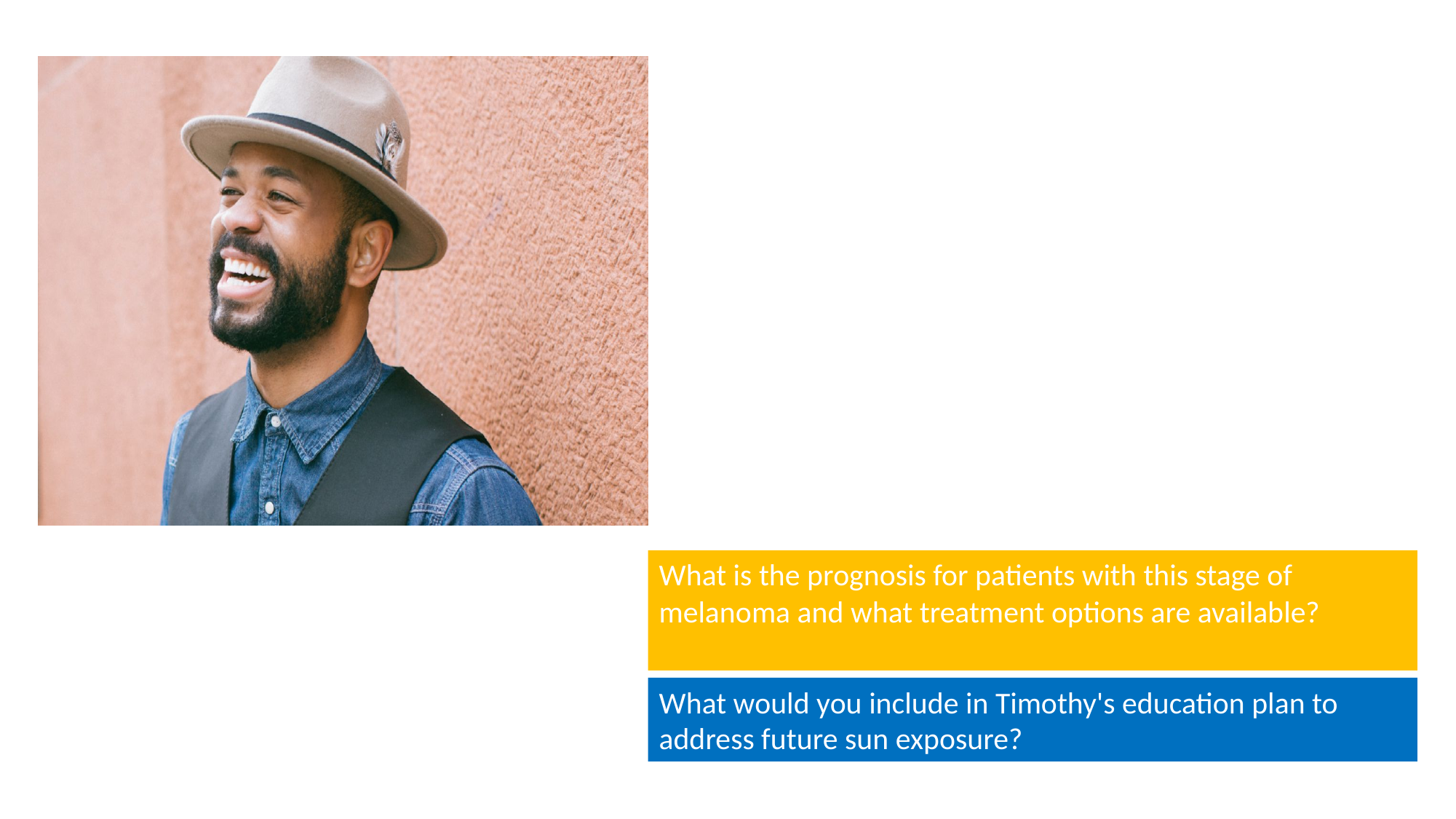

What is the prognosis for patients with this stage of melanoma and what treatment options are available?
What would you include in Timothy's education plan to address future sun exposure?

## Slide 41
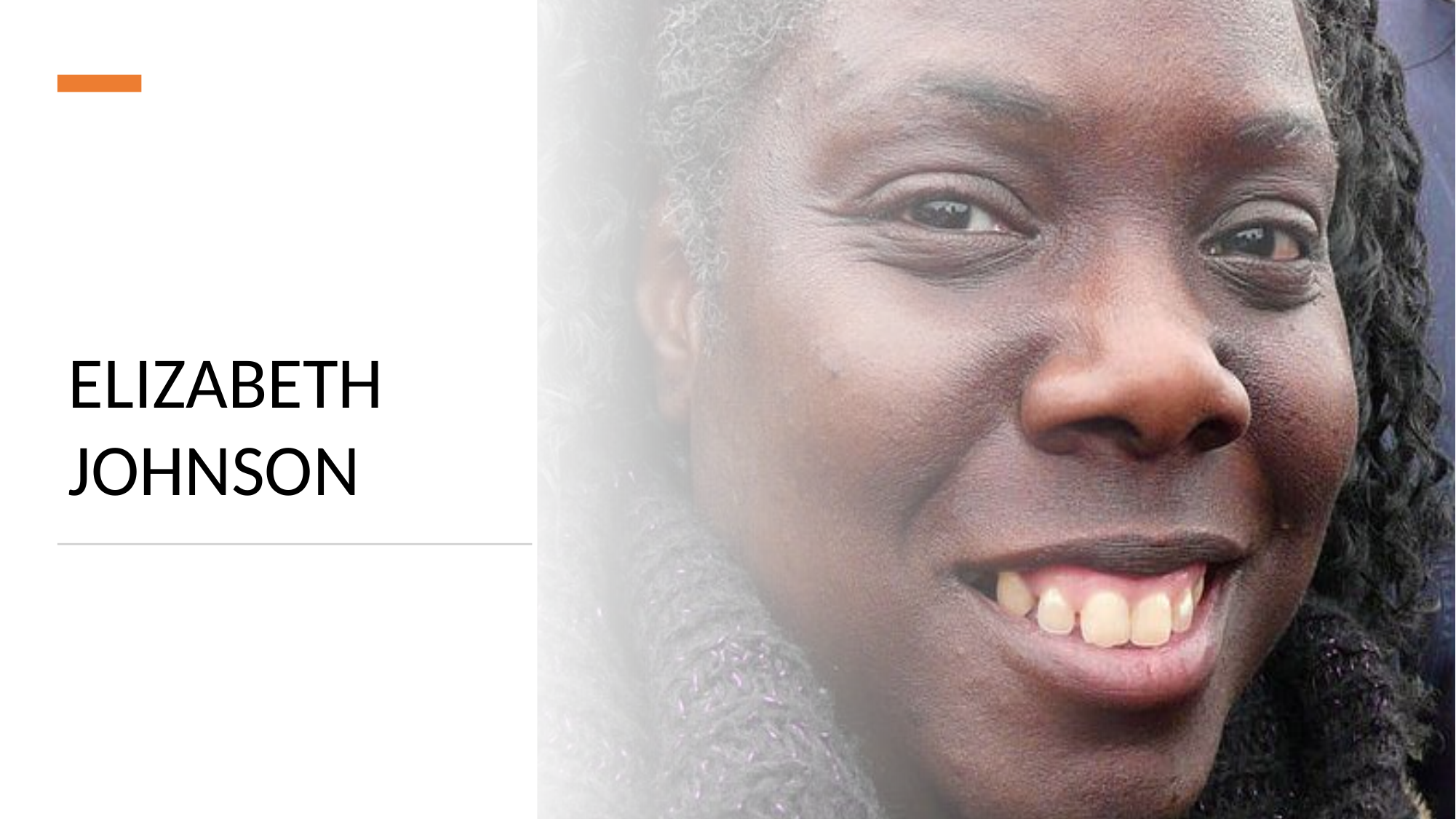

ELIZABETH
JOHNSON

## Slide 42
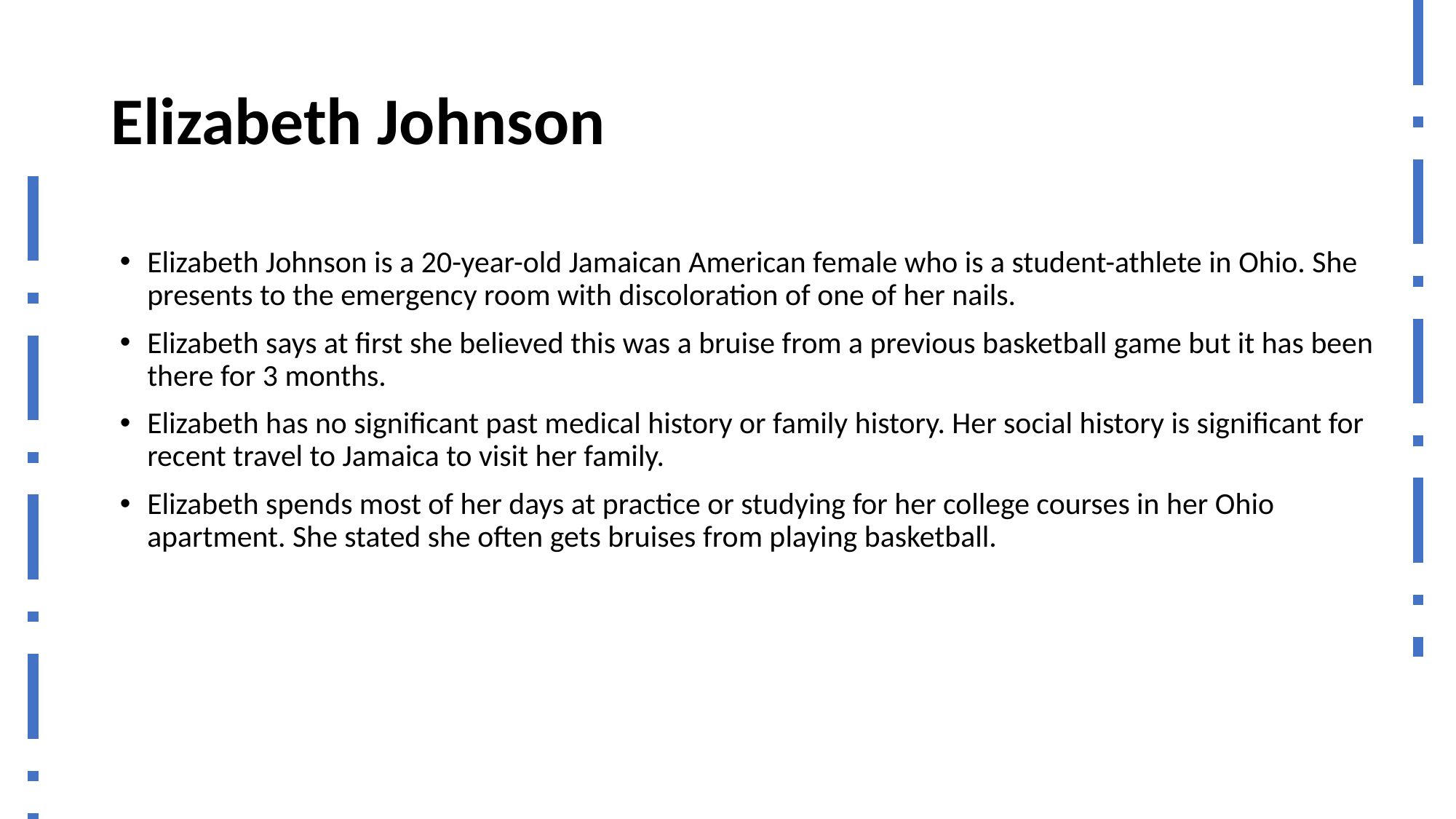

# Elizabeth Johnson
Elizabeth Johnson is a 20-year-old Jamaican American female who is a student-athlete in Ohio. She presents to the emergency room with discoloration of one of her nails.
Elizabeth says at first she believed this was a bruise from a previous basketball game but it has been there for 3 months.
Elizabeth has no significant past medical history or family history. Her social history is significant for recent travel to Jamaica to visit her family.
Elizabeth spends most of her days at practice or studying for her college courses in her Ohio apartment. She stated she often gets bruises from playing basketball.

## Slide 43
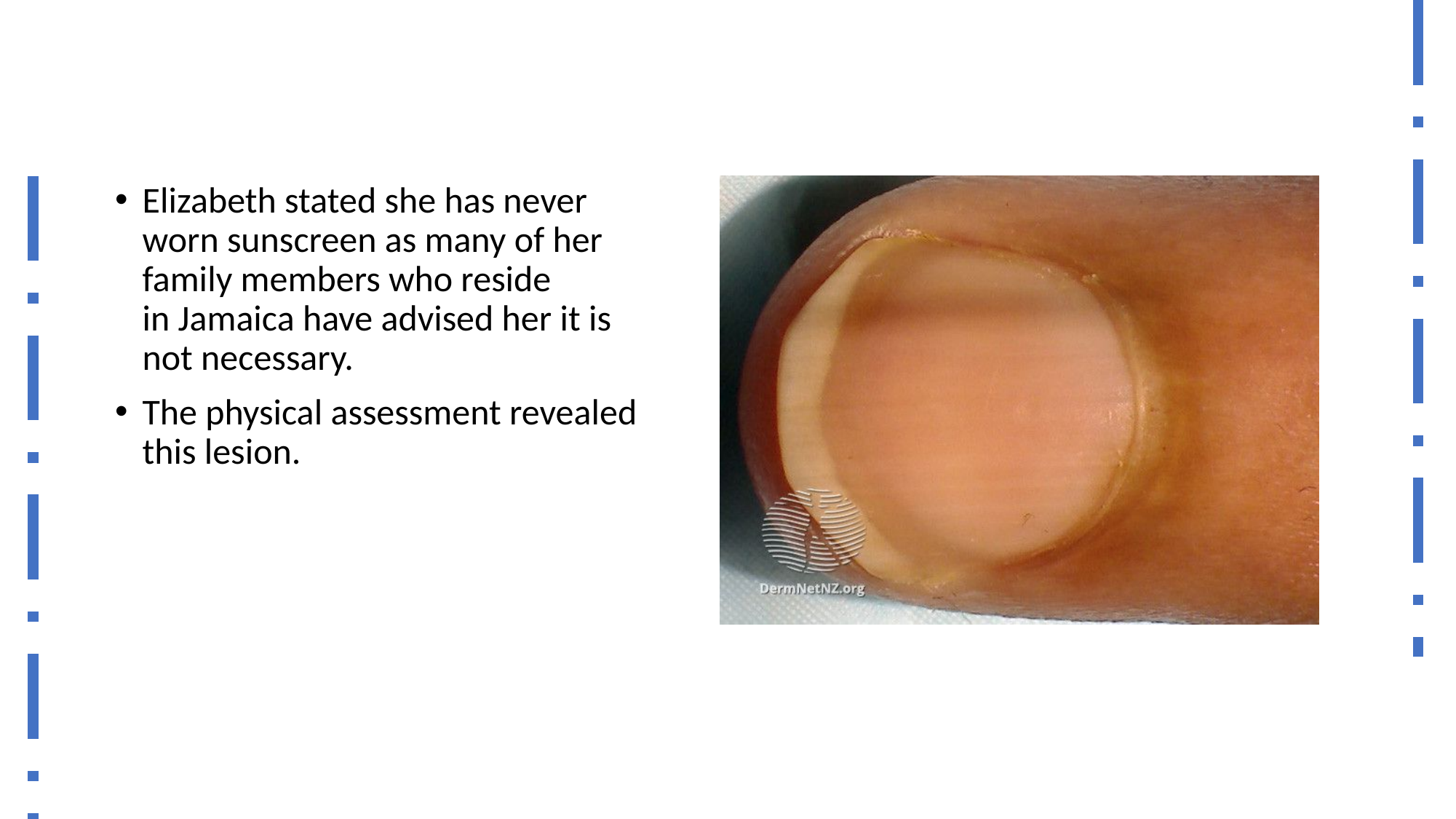

Elizabeth stated she has never worn sunscreen as many of her family members who reside in Jamaica have advised her it is not necessary.
The physical assessment revealed this lesion.

## Slide 44
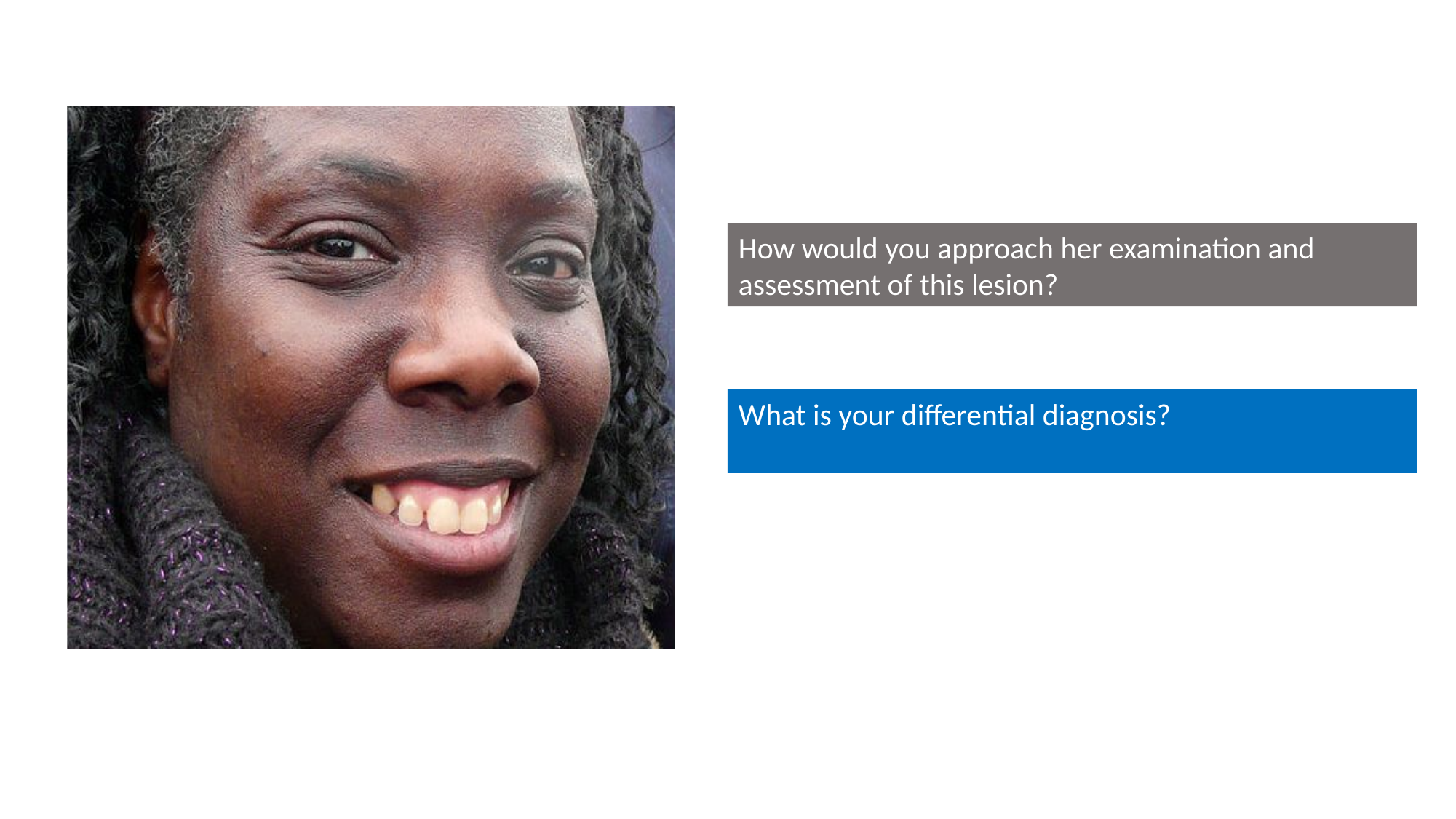

How would you approach her examination and assessment of this lesion?
What is your differential diagnosis?

## Slide 45
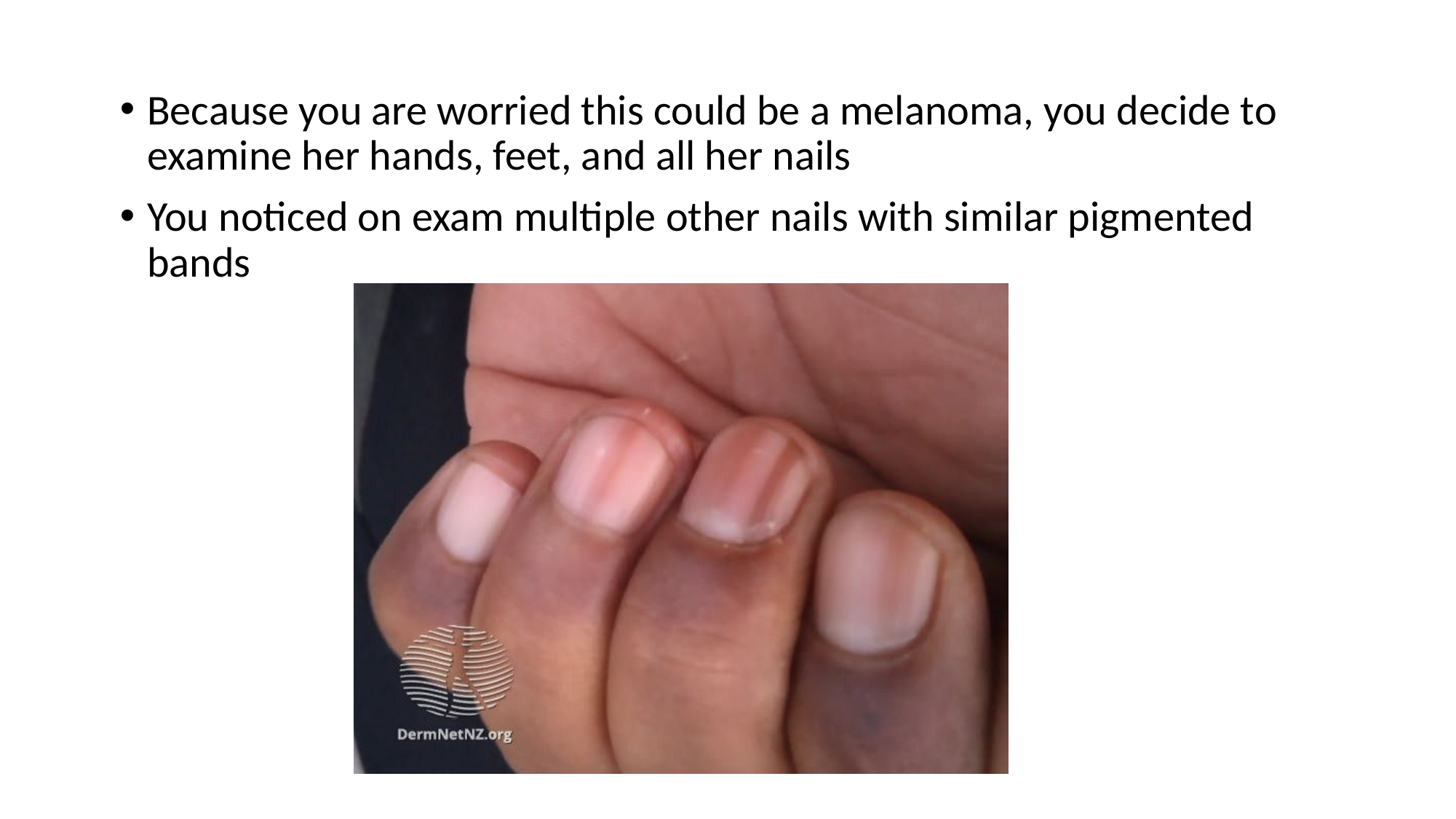

Because you are worried this could be a melanoma, you decide to examine her hands, feet, and all her nails
You noticed on exam multiple other nails with similar pigmented bands

## Slide 46
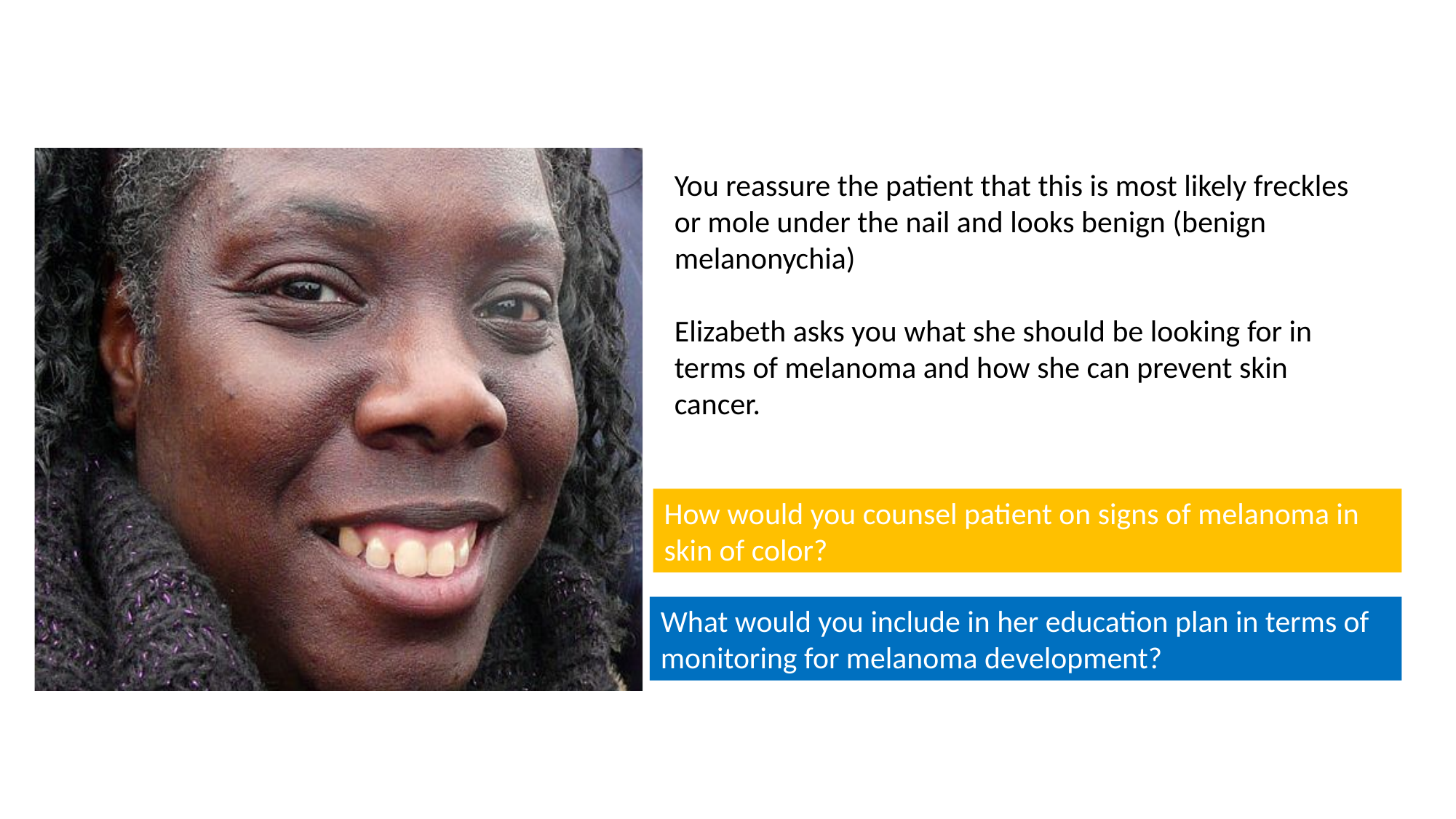

You reassure the patient that this is most likely freckles or mole under the nail and looks benign (benign melanonychia)
Elizabeth asks you what she should be looking for in terms of melanoma and how she can prevent skin cancer.
How would you counsel patient on signs of melanoma in skin of color?
What would you include in her education plan in terms of monitoring for melanoma development?

## Slide 47
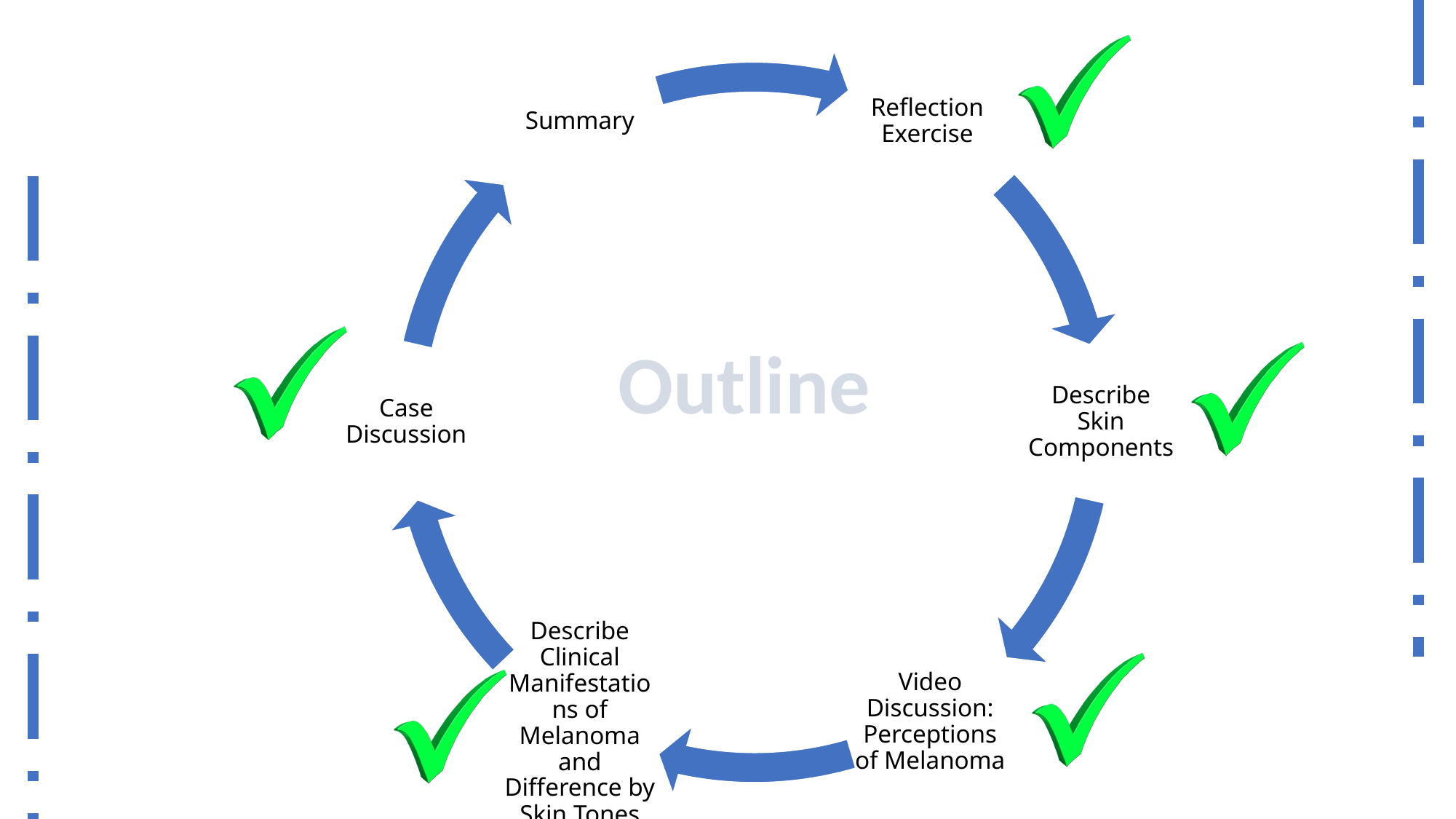

Summary
Reflection Exercise
Case Discussion
Describe Skin Components
Video Discussion: Perceptions of Melanoma
Describe Clinical Manifestations of Melanoma and Difference by Skin Tones
Outline

## Slide 48
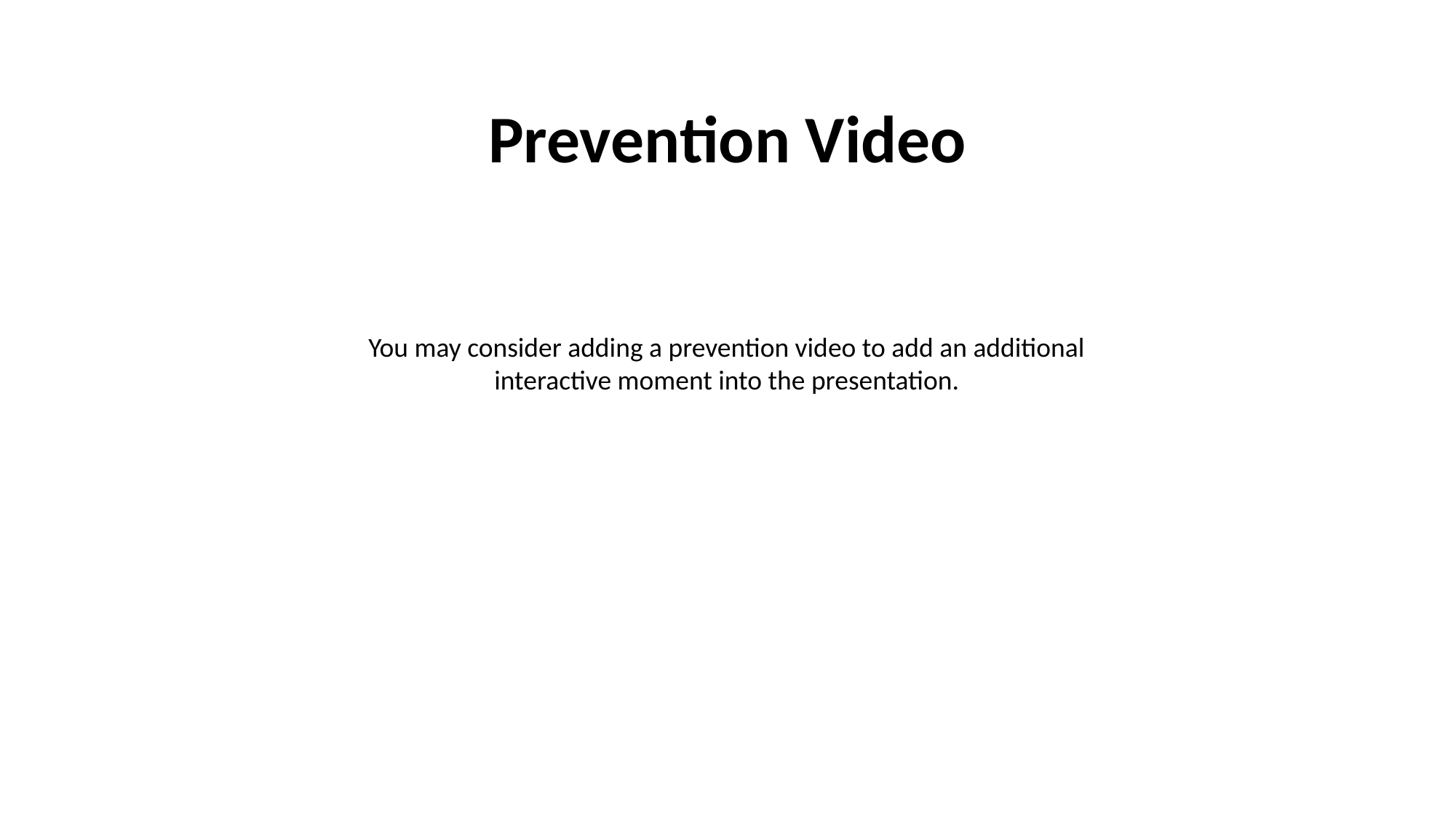

# Prevention Video
You may consider adding a prevention video to add an additional interactive moment into the presentation.

## Slide 49
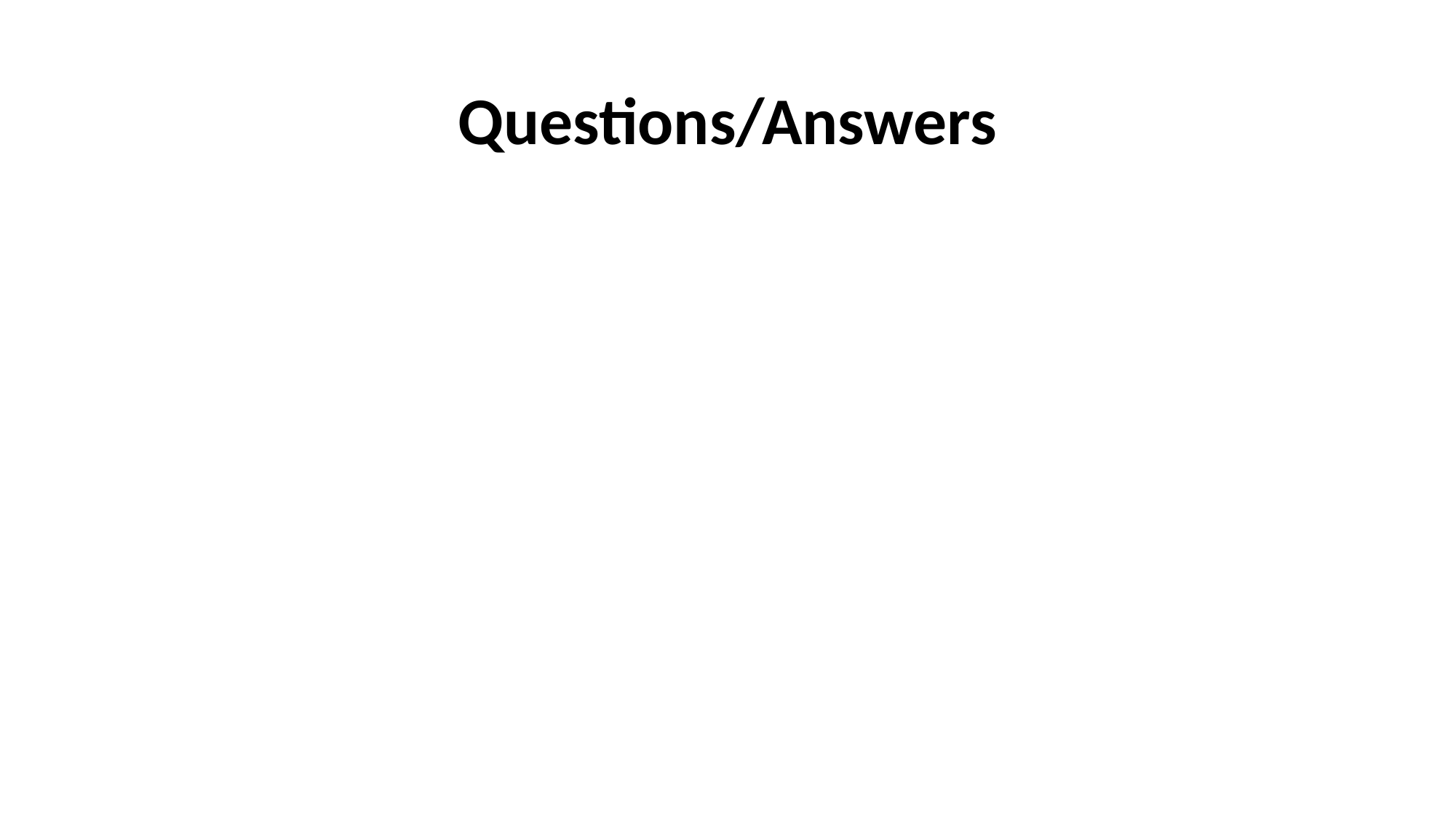

# Questions/Answers
