# Supplementary material for: Melanoma: Does It Present Differently in Darker Skin Tones?
Source: MedEdPORTAL. 2023 May 9;19:11311. doi: 10.15766/mep_2374-8265.11311 (PMC10166772; doi:10.15766/mep_2374-8265.11311)
Supplement: Supplementary file 1 — Melanoma Presentation.pptxMelanoma Myth.mp4Facilitator Guide.docxEvaluation Form.docx [file mep_2374-8265.11311-s001.zip › C. Facilitator Guide.docx]

**MELANOMA: DOES IT PRESENT DIFFERENTLY IN DARKER SKIN TONES?**

**Overall Goals**

The goals of this module are to help health professional trainees gain a greater awareness of melanoma presentation in different skin tones in order to reduce advanced stage melanoma especially among individuals of darker skin tones.

**Workshop Objectives**

- Describe the structure and components of the skin
- Describe the etiology and clinical manifestation of melanoma and its subtypes
- Recognize various melanoma presentations, especially in darker skin tones
- List melanoma prevention and treatment options

**Workshop Materials**

Evaluation forms and pens

Computer setup and connection to projector (if in person)

Video communication software (Zoom, WebEx, etc.)

**Suggested Agenda and Timeline (~75 minutes)**

Pre-Workshop Evaluation- 2 minutes

Slides 1-3: Introduction, Objectives, Presentation Road Map - 5 minutes

Slides 4-5: Reflection, Fitzpatrick Scale - 3 minutes

Slide 6: Transition Slide (Workshop Roadmap) - 1 minute

Slides 7-12: Describe Skin Component - 10 minutes

Slide 13: Transition Slide (Workshop Roadmap) - 1 minute

Slide 14: Video Discussion - Perceptions of Melanoma - 8 minutes

Slide 15: Transition Slide - 1 minute

Slides 16-31: Clinical Manifestation of Melanoma - 15 minutes

Slide 32: Transition Slide (Workshop Roadmap) - 1 minute

Slides 33-46: Case Discussion - 15 minutes

Slide 47: Transition Slide - 1 minute

Slide 48: Summary Video - 4 minutes

Slide 49: Questions - 5 minutes

Post-Workshop Evaluation- 2 minutes

**Suggested Reading Material and Resources for Facilitators in Preparation for Workshop**

- Fitzpatrick's Color Atlas & Synopsis of Clinical Dermatology, 5th Edition
- Fitzpatrick's Dermatology in General Medicine, 7th Edition
- National Cancer Institute, "Melanoma," www.Cancer.Gov/Cancertopics/Types/Melanoma
- Ragel El, Et Al. Cutaneous Melanoma: Update On Prevention, Screening, Diagnosis, and Treatment. Am Fam Physician 2005; 15:269-276.
- Harvey VM, Patel H, Sandhu S, Wallington SF, Hinds G. Social Determinants Of Racial And Ethnic Disparities In Cutaneous Melanoma Outcomes. Cancer Control. 2014 Oct;21(4):343-9.

**Slide Instructions**

This module should be modified by updating statistics and information from a literature review. Facilitators may also choose to divide the large group into small groups for the case discussions.

The number of facilitators will depend on the option for case discussions chosen. Two facilitators are sufficient to implement the interactive workshop. We suggest two facilitators, one for each case, if the chosen option is to discuss cases in small groups prior to the large group debriefing. At least one facilitator should have a strong knowledge base in dermatology.

Slide 1: Title Slide

Add facilitator(s) name and affiliation. The facilitator(s) should introduce themselves to the audience and discuss their roles in their respective institution(s). The facilitator(s) may choose to mention they are following the content and guidelines of this peer- reviewed module.

Slide 2:

Please read the learning objectives

Slide 3:

Please review the presentation outline for participants.

Reflection Exercise

Describe Skin Components

Video Discussion: Perceptions of Melanoma

Describe Clinical Manifestations of Melanoma and Difference by Skin Tones

Case Discussion

Summary

Slide 4:

Reflection Activity:

Provide the following instructions to participants. Take one minute and place in the chat box the following two questions.

In terms of race/ethnicity, how do you describe yourself on the Census? How do you describe your skin tone?

As you know there is much variation in racial and ethnic identity as well as skin tone. Race and skin tone are two separate constructs and are not interchangeable or synonymous. It is important that we distinguish this because we should not assume that one's racial or ethnic identification predicts a person's skin tone. Race is defined as the identification of someone amongst which social group they belong. Skin tone is the color of someone’s skin that can be the result of genetic variation or changes due to pathologic processes. Racial inequities in melanoma are multifactorial and are the product of individual behavior and structural racism. Through this session we hope to address the information gap about melanoma in darker skin individuals as a result of structural racism in clinician training.

Slide 5:

The facilitator should review the Fitzpatrick Scale and discuss its potential clinical application. The scale is one classification system for skin tone and their reactions to sun exposure. Type 1 is the fairest complexion and Type VI is the darkest complexion.

Slide 6:

Transition Slide.

The facilitator should provide a brief explanation of what's expected to be covered under each heading. For example, “We are now moving on to Describing the Skin Components." Under this section, we will discuss the skin structure, and provide an overview of what melanoma is, staging of melanoma and some comparative statistics.

Slide 7:

Title/Transition slide.

Slide 8:

The human skin is made up of three layers; the epidermis, the dermis, and the subcutaneous tissue. The most superficial layer, the epidermis, consists of basal cells, squamous cells, and melanocytes. The middle layer is the dermis which contains hair follicles, glands, and collagen. The deepest layer is the subcutaneous fat is where you will find adipose tissue.

Slide 9:

Melanocytes - these cells are found in the outermost layer of the skin, the epidermis. These cells produce melanin, which are stored in melanosomes, and give the skin its pigmented appearance. Darker skin individuals have higher production and distribution of melanosomes and melanin in their skin.

Slide 10:

Before we dive right into melanoma, first we will paint a picture of what this disease is, and how it affects our population. According to the National Cancer Institute, melanoma is defined as a form of cancer that begins in melanocytes. In the skin, it may develop as a new lesion or in a pre-existing pigmented lesion (such as a benign mole that suffers mutation and becomes malignant). Melanomas can also develop in other organs that contain melanocytes, such as in the eye or in the intestines. Sun exposure, UV radiation, and genetics are known risk factors in lighter complexion but risk factors in darker complexed individuals are not as clear.

Slide 11:

After someone is diagnosed with melanoma, the next step often taken is to determine if the disease has spread, and if so, how far. This process is known as staging which according to the American Joint Committee on Cancer 8th Edition includes the classic TNM system known for other cancers and stands for tumor (T) which in melanoma represent the thickness of the tumor and further subdivide in “a” without ulceration and “b” with ulceration, node (N), and metastasis (M). Stage 0 represents melanoma in situ which has the highest survival rate (Tis N0 M0) to stage IV (any T, any N, and M1 meaning positive metastasis) which has the lowest one. Management will vary with staging.

Reference: Melanoma Skin Cancer Stages. <https://www.cancer.org/cancer/melanoma-skin-cancer/detection-diagnosis-staging/melanoma-skin-cancer-stages.html> Accessed on May 15, 2022

Slide 12:

African Americans and Asians present most commonly with acral lentiginous melanoma. Data shows that non-Hispanic Black Americans are diagnosed at a later stage meaning generally poorer prognosis. Superficial spreading melanoma is the most common in Hispanic groups, but still the acral variant is very common. Asians/Pacific Islanders have the highest proportion of mucosal melanomas across races.

Culp M, Lunsford N. Melanoma Among Non-Hispanic Black Americans. Centers for Disease Control and Prevention. Preventing Chronic Disease.

https://www.cdc.gov/pcd/issues/2019/18_0640.htm. Published June 20, 2019. Accessed January 4, 2021

Higgins S, Nazemi A, Feinstein S, Chow M, Wysong A. Clinical Presentations of Melanoma in African Americans, Hispanics, and Asians. Dermatol Surg. 2019 Jun;45(6):791-801. doi: 10.1097/DSS.0000000000001759. PMID: 30614836.

Slide 13:

Transition Slide. The facilitator should provide a brief explanation of what's expected to be covered under each heading.

In this section of the workshop, my colleague will be presenting a video illustrating some common misperceptions about melanoma.

Slide 14:

Video Discussion – This video highlights some common misperceptions of melanoma. The facilitator should engage participants and inquire what they have heard about melanoma from friends and family. The facilitator should address each question and provide the correct True or False response.

Question One: Individuals with darker skin can develop melanoma. The answer is TRUE

Question Two: At the time of diagnosis, melanomas in darker skin individuals are usually low risk and less aggressive compared to lighter skin individuals. The answer is FALSE

Question Three: Sun exposure is a major factor causing melanoma in darker skin individuals. The answer is FALSE

Alternative Video to One in Module: <https://www.youtube.com/watch?v=yIu6WC5wxpU>

Alternative Discussion - This is one of many examples of patients being misinformed concerning melanoma in darker skin. There are many misconceptions of melanoma in darker skin individuals. I am going to ask you a series of questions and I want you all to raise your hand for true or false.

Question One: Individuals with darker skin can develop melanoma. *Raise hands for true and raise hands for false* The answer is TRUE

Question Two: At the time of diagnosis, melanomas in darker skin individuals are usually low risk and less aggressive compared to lighter skin individuals. *Raise hands for true and raise hands for false* The answer is FALSE

Question Three: Sun exposure is a major factor causing melanoma in darker skin individuals. *Raise hands for true and raise hands for false* The answer is FALSE

Slide 15:

Transition slide. The facilitator should provide a brief explanation of what's expected to be covered under each heading. In this section, we will discuss the clinical manifestation of melanoma in darker skin. We will also discuss methods used to diagnose melanoma and risk factors for melanoma. Additionally, we will review a medical illustration of melanoma, and discuss its prevention and treatment. Lastly we will cover some common dermatologic conditions that mimic melanoma.

Slide 16:

Here is a very common method used to diagnose melanoma, the ABCDE method.

Asymmetry - Melanoma is often asymmetrical, which means the shape isn't uniform. Non- cancerous moles are typically uniform and symmetrical in shape.

Border - Melanoma often has borders that aren't well defined or are irregular in shape, whereas

non-cancerous moles usually have smooth, well-defined borders.

Color - Melanoma lesions are often more than one color or shade. Moles that are benign are typically one color.

Diameter - Melanoma growths are normally larger than 6mm in diameter, which is about the diameter of a standard pencil eraser.

Evolution - Melanoma will often change characteristics, such as size, shape, or color. Unlike most benign moles, melanoma tends to change over time. If you have a mole or skin growth, watch it for signs of changes. If you notice any of the ABCDEs of melanoma, make an appointment right away to be evaluated by a dermatologist.

Since patients with darker skin have higher incidence of melanoma in non-sun exposed areas (such as acral surfaces, mucosa, lower extremity) when evaluating these patients a very thorough exam and consideration of atypical presentations of melanoma is critical.

Slide 17:

Having less pigment in the skin increases the risk of melanoma, **but anyone can develop melanoma, including darker skin individuals**.

Risk factors for melanoma in darker skin individuals:

Family History and mutations in certain genes is linked to melanoma;

Additional reported melanoma risk factors in people of color include albinism, radiation therapy, immunosuppression, burn scars or trauma (due to increased and persistent inflammation and chronic scarring), and preexisting pigmented lesions (especially on hands, feet, and mouth)

It is important to note that although UV radiation is a major risk factor for melanoma and other skin cancers in light skin individuals, it is unclear what role this plays in melanoma development in skin of color populations. Darker skin has more UV protection given higher levels of melanosomes. However, large validated studies looking at the role of UV in melanoma in skin of color populations are lacking.

Although this remains unclear, it is well-accepted that excessive UV exposure can lead to other skin cancers, including SCC and BCC in sun-exposed areas of skin of color individuals. Given the spectrum of skin tones found in any race, it is still recommended that all individuals be counseled on potential harm of excessive sun damage and ways to practice sun safety.

Slide 18:

The ABCDEF mnemonic is helpful in understanding characteristics associated with nail melanoma.

Slides 19-21:

Examples of different presentations of melanoma.

Slide 22:

Slide Transition – Melanoma prevention and treatment

Slide 23:

How can melanoma be prevented?

For example: patient education, self-exam, skin protection and sunscreen. Complete physical exam including thorough examination of the mouth, feet, hands, and nails is especially important. Be aware of variations of presentations and have a high index of suspicion for any atypical lesion on darker skin.

Slide 24:

Management of Melanoma.

Emphasize surveillance and seek a physician's evaluation for changes or new skin lesions.

Management of melanoma is dependent on the stage and the patient. Includes surveillance, surgery, radiation, chemotherapy, or immunotherapy.

Slide 25:

Management of Melanoma (cont.)

These are some more detailed ways in which you can treat melanoma depending on the staging. When caught early often melanoma can be managed with surgery.

Slide 26:

Prognosis for melanoma is poor, especially when diagnosed at a later stage. Darker skinned individuals tend to be diagnosed at late stages and tend to have poorer prognosis. If detected while still localized, the 5 year survival rate is 99%.

Slide 27:

Transition Slide. Common Lesions in Darker Skin Individuals Often Mistaken for Melanoma

Slides 28-31:

Benign pigmented lesions.
Slide 31: please note that melanoma could present as melanonychia. It is important to obtain a comprehensive history and examine all digits. Melanonychia involving a single digit, developing later in life, darker in color, thicker or irregular, and changing should be further examined to rule out malignancy.

Slide 32:

Transition slide.

The facilitator should provide a brief explanation of what's expected to be covered under each heading.

Now we will discuss our case studies. Allow a 1-minute break if applicable.

Slide 33:

Transition – Case Study Discussion

Slides 34-35:

Read the slides Timothy Adams Case.

Facilitator will lead the discussion and reflection session by directing questions to the attendants and engaging them to discuss and reflect on each question. We suggest each question be discussed for 1-2 minutes.

The questions with the recommended discussion points/answers are as follows:

Slide 36:

How would you counter the health disparities faced by Timothy?

Given his work outside still at heightened risk for melanoma due to sun exposure. Depending on his work, may or may have flexibility in his schedule to see a specialist or primary care provider. Also depending on his position may not have proper insurance.

Slide 37:

How do you describe this lesion?

Response – Use the mnemonic ABCDE

What are you concerned about?

Response - melanoma

What would you do next?

Refer to a dermatologist to be seen within 1-2 weeks for possible wide local excision.

Slide 38:

Are there any risk factors contributing to Timothy’s condition?

Acral melanomas are more common in darker skin individuals. Although it is unclear the extent of UV role in development of melanoma in skin of color individuals, potential risk factors include working outside with greater sun exposure. Potentially, chronic trauma to the foot could be a risk factor, given his professionl (but this was not mentioned in his history or seen on exam).

Slide 39:

Self-explanatory

Slide 40:

What is the prognosis for patients with this stage of melanoma and what treatment options are available?

Given localized nature – 99% 5-year survival.

What would you include in Timothy's education plan to address future sun exposure?

Encourage covering skin-exposed areas when working outdoors.

Do self-exams to assess for additional lesions.

Apply skin protection, such as sunscreen.

Follow-up with a health care provider.

Slides 41-43:

Elizabeth Johnson Case

Slide 44:

How would you approach her examination and assessment of this lesion?

A proper examination would involve examining her skin; both skin-exposed and non-exposed areas and examining all her fingernails and toenails. Pay attention to the color, size, and pattern of the pigmented band or her nail(s).

What is your differential diagnosis?

Longitudinal melanonychia which could represent a benign mole, freckle, but also a melanoma

Slide 45:

Self-explanatory

Slide 46:

How would you counsel patients on signs of melanoma in skin of color?

Given the pigmented band is well-defined, light in color, present in multiple nails, and started earlier in life this seems to be benign and there is no need for biopsy. I would advise the patient to return for evaluation in case the pigmentation in the nails becomes darker, thicker, symptomatic, or her nail significantly changes.

Share with the patient the ABCDEF of nail melanoma.

A- Age, most common 50-70

B- Brown or black band in nail

B- Breadth > 3 mm

B- Border irregular

C- Changing size, shape

D- Digit, most common in thumb

E- Extension of pigment

F- Family history of melanoma

What would you include in her education plan in terms of monitoring for melanoma development?

Continue to conduct skin and nail exams to assess for any changes as indicated in the A-F of nail melanoma and for skin assessment of the A-E classical method to diagnose melanoma.

Slide 47:

The facilitator should provide a brief explanation of what’s expected to be covered under each heading.

Slide 48:

Summary video.

Please take a few minutes to watch this YouTube video.

Slide 49:

Questions and Answers

**Additional frequently asked questions**

Which organizations provide resources for skin of color dermatology?

-Visual Dx, Skin of Color Society, and NMA Dermatology are organizations that provide resources, research, and awareness of dermatologic conditions affecting skin of color

Are there any recommended textbooks for skin of color dermatology?

-Taylor and Kelly’s Dermatology for Skin of Color, Skin of Color: A Practical Guide to Dermatologic Diagnosis and Treatment, and Dermatology, and Diagnosing Skin Disease in Skin of Color, an Issue of Dermatologic Clinics.
