# Supplementary material for: Melanoma: Does It Present Differently in Darker Skin Tones?
Source: MedEdPORTAL. 2023 May 9;19:11311. doi: 10.15766/mep_2374-8265.11311 (PMC10166772; doi:10.15766/mep_2374-8265.11311)
Supplement: Supplementary file 1 — Melanoma Presentation.pptxMelanoma Myth.mp4Facilitator Guide.docxEvaluation Form.docx [file mep_2374-8265.11311-s001.zip › D. Evaluation Form.docx]

**Pre-Test**

**Step I: Demographic**

**Select ONE that describes your current professional role:**

- Registered Nurse
- Nursing Student
- Medical Student
- Medical Resident or Fellow
- Faculty
- Other _________________

**Select all that apply:**

- Female
- Male
- Transgender or Gender Nonconforming
- Different Identity
- Other _________________

**Select all that apply:**

- Latina/o/x/e, Hispanic or of Spanish Origin+
- Black/African American
- White
- Asian
- American Indian/Alaska Native
- Other ________________

**Step II: Self-Assessment**

| **Please rate how much CONFIDENCE you have in your ability to…** | **No**  **Confidence**  **1** | **2** | **3** | **4** | **Complete**  **Confidence**  **5** |
| --- | --- | --- | --- | --- | --- |
| Obj 1: Describe the structure and components of the skin | 1 | 2 | 3 | 4 | 5 |
| Obj 2: Describe the etiology and clinical manifestation of melanoma and its subtypes​ | 1 | 2 | 3 | 4 | 5 |
| Obj 3: Recognize various melanoma presentations, especially in darker skin tones | 1 | 2 | 3 | 4 | 5 |
| Obj 4: List melanoma prevention and treatment options | 1 | 2 | 3 | 4 | 5 |

**Post-Test**

**Step I: Self-Assessment**

| **Please rate how much CONFIDENCE you have in your ability to…** | **No**  **Confidence**  **1** | **2** | **3** | **4** | **Complete**  **Confidence**  **5** |
| --- | --- | --- | --- | --- | --- |
| Obj 1: Describe the structure and components of the skin | 1 | 2 | 3 | 4 | 5 |
| Obj 2: Describe the etiology and clinical manifestation of melanoma and its subtypes | 1 | 2 | 3 | 4 | 5 |
| Obj 3: Recognize various melanoma presentations, especially in darker skin tones | 1 | 2 | 3 | 4 | 5 |
| Obj 4: List melanoma prevention and treatment options | 1 | 2 | 3 | 4 | 5 |

**Step II: Comments**

**Please answer the following questions:**

1. What did you like about this workshop?
2. What suggestions do you have to improve this workshop?
