# Supplementary material for: Melanoma: Does It Present Differently in Darker Skin Tones?
Source: MedEdPORTAL. 2023 May 9;19:11311. doi: 10.15766/mep_2374-8265.11311 (PMC10166772; doi:10.15766/mep_2374-8265.11311)
Supplement: Supplementary file 1 — Melanoma Presentation.pptxMelanoma Myth.mp4Facilitator Guide.docxEvaluation Form.docx [file mep_2374-8265.11311-s001.zip › Educational Summary Report_11311.pdf]

MedEdPORTAL® The AAMC Journal of Teaching and Learning Resources

Original Publication Open Access

Melanoma: Does It Present Differently in Darker Skin Tones?

Brandon Thompson, RN, BSN†, Toni Jenkins, MS†, John Paul Sánchez, MD, MPH*, Matthew Frederick, DO, Alba Posligua-Alban, MD, Naiara Sbroggio Barbosa, MD

*Corresponding author: jopsanchez@salud.unm.edu
†Co-primary authors

Abstract

Introduction: There are vast differences in clinical presentations of melanoma across skin tones. Individuals with darker skin tones tend to have a higher prevalence of advanced-stage melanoma, which correlates with increased mortality. We designed this interactive workshop to increase nursing and medical trainees' awareness of the epidemiology, prevention, and treatment of melanoma in individuals of darker skin tones. **Methods:** The Kern model was used in the design, implementation, and evaluation of the workshop. The 75-minute workshop consisted of a PowerPoint presentation, video-based reflection activities, and case studies. Evaluation consisted of pre- and postworkshop questionnaires. The workshop was implemented two times among 63 nursing students, 11 medical students/residents, and six medical faculty. **Results:** Seventy-one participants completed the pre- and postworkshop evaluations. A comparison of pre- and postworkshop responses utilizing the Wilcoxon matched-pair signed rank test showed a statistically significant increase in learners' confidence to address each learning objective. **Discussion:** Through this interactive educational presentation, medical and nursing trainees can gain heightened awareness of melanoma across various skin tones, especially unique presentations in darker skin tones.

Keywords
Melanoma, Skin Tone, Case-Based Learning, Cultural Competence, Dermatology, Anti-racism, Diversity, Equity, Inclusion

Educational Objectives

By the end of this activity, learners will be able to:

1. Describe the structure and components of the skin.
2. Describe the etiology and clinical manifestation of melanoma and its subtypes.
3. Recognize various melanoma presentations, especially in darker skin tones.
4. List melanoma prevention and treatment options.

Introduction

The incidence of melanoma continues to rise despite efforts at educating practitioners and the public about its causes and prevention strategies.[1] Most educational programs are focused on the subset of the population with the highest incidence: individuals of light skin tones. However, melanoma occurs as well in individuals of darker skin tones.[1]

Citation:
Thompson B, Jenkins T, Sánchez JP, Frederick M, Posligua-Alban A, Barbosa NS. Melanoma: does it present differently in darker skin tones? *MedEdPORTAL*. 2023;19:11311.
https://doi.org/10.15766/mep_2374-8265.11311

Historically, melanoma research has been framed by racial differences and by skin tone differences. Prior research focusing on melanoma by racial groups noted that approximately 1% of skin cancers occur in Black-identified patients and that mortality for Black-identified individuals is two- to threefold higher than for White-identified populations.[2] A significant factor in racial disparities is advanced stage at diagnosis.[3,4] When comparing by skin tone using the Fitzpatrick skin scale in a dichotomous fashion, Fitzpatrick type I-III skin is often labeled as Caucasian and Fitzpatrick type IV-VI as skin of color individuals (SCIs).[5] Research on melanoma reported by skin tone has shown that practitioners (a) tend to be unaware that 70% of melanomas in SCIs arise on the often-overlooked plantar surface of the foot and (b) lack proficiency in differentiating benign from malignant lesions in SCIs.[6,7] In addition, SCIs may not be appropriately counseled by practitioners to get routine skin exams. The knowledge gap in this area has been identified by some experts as "a prime example of structural inequalities in medicine."[8]

In response to the lack of dermatologic images of darker skin tones, a growing body of print literature and atlases has been developed, including Taylor and Elbuluk's *Color*

Copyright © 2023 Thompson et al. This is an open-access publication distributed under the terms of the Creative Commons Attribution license.

Atlas and Synopsis for Skin of Color,[9] Jackson-Richards and Pandya's *Dermatology Atlas for Skin of Color,*[10] and Eleryan and Friedman's *A Diverse and Inclusive Atlas,*[11] among others. Online resources that feature diverse skin tones include educational materials from the American Academy of Dermatology Skin of Color Curriculum[12] and the Skin of Color Society Dermatology E-Learning + Equity Platform,[13] as well as online atlases published by VisualDX.com[14] and the University of New Mexico.[15]

From 2016 to 2021, *MedEdPORTAL* published four educational resources referencing *melanoma* as a keyword. One of these four uses general dermatology and musculoskeletal medicine to advance medical Spanish communication skills of medical students.[16] The other three resources focus on addressing general dermatology knowledge among preclinical medical students and novice health care workers.[17-19] All four publications were implemented at single sites. There are no materials currently in *MedEdPORTAL* addressing the important differences in presentation of melanoma amongst SCIs.

Our 75-minute interactive workshop aims to help learners (i.e., nursing students, medical students, and residents) understand that patients with darker skin tones (a) can develop melanoma, (b) may have a presentation different from those with lighter skin tones, and (c) tend to have more advanced/aggressive melanoma at the time of diagnosis. We utilized the Kern model of curriculum development in the creation of our content.[20] A needs assessment revealed a paucity of educational material on this topic.[21] Our workshop can help fulfill medical school and residency requirements, such as Liaison Committee on Medical Education element 3.3 Diversity/Pipeline Programs and Partnership and element 7.6 Cultural Competence and Health Care Disparities[22]; ACGME Interpersonal and Communication Skills Competence[23]; and the AAMC's Cultural Competence Education Domains I and IV[24] and Diversity, Equity, and Inclusion Competencies Ia and Ib.[25]

Methods

A nursing student, a medical student, a dermatology resident, a dermatology attending, and two emergency medicine faculty developed, implemented, and/or evaluated this workshop. The students conceptualized the workshop, and the faculty provided guidance on clinical components and health disparities content. Authors Brandon Thompson, Toni Jenkins, and Naiara Sbroggio Barbosa copresented the workshop on two occasions: at the Building the Next Generation of Academic Physicians (BNGAP) Medical Education Conference and at the Nursing Program of Central New Mexico Community College.

The workshop employed three instructional strategies: an interactive Microsoft PowerPoint (PPT) presentation, reflection exercises, and case discussion. The PPT presentation (Appendix A) began with a brief overview of what melanoma is and how it affects individuals with darker skin tones. To highlight the spectrum of skin tone and distinguish skin tone from racial categories, we asked participants to self-identify utilizing a pigmentary phototype (the Fitzpatrick scale). An interview clip (Appendix B) addressing myths related to pigmented skin was incorporated into the presentation. The PPT proceeded with an outline of the epidemiology of skin cancer in SCIs as well as a basic overview of dermatopathology. Attention then turned to the clinical presentation of melanoma in SCIs, with multiple photographic references for both malignant and benign lesions. The workshop also included two case discussions for participants to apply new knowledge while discussing health disparities, risk factors, and prevention of melanoma. A facilitator guide (Appendix C) described how to implement each component of the workshop. Participants were asked to complete pre- and postworkshop self-assessments (Appendix D) based on the presentation's learning objectives and content.

The workshop took 75 minutes to implement. Approximately a third of the time was spent on interactive reflection and case discussions (25 minutes), with the rest dedicated to didactic material and administration of the pre- and postworkshop evaluations.

Materials included audiovisual equipment, virtual technology, and online (or, if in person, printed) evaluation forms. We implemented both sessions virtually due to COVID restrictions.

Both pre- and postworkshop questionnaires contained self-assessments of confidence and knowledge questions to coincide with Kirkpatrick levels 1 and 2 of evaluation. Participants' confidence in meeting the workshop's learning objectives was assessed on a 5-point scale (1 = *no confidence,* 5 = *complete confidence*). The preworkshop questionnaires contained demographic questions, whereas the postworkshop questionnaires contained qualitative feedback questions. The survey was created by the coauthors and did not undergo validity testing. Descriptive and nonparametric bivariate analyses were conducted using SPSS version 28.0.

Future facilitators should have a general knowledge of basic skin anatomy and dermatopathology. They should consider setting aside 2-3 hours to review the PPT presentation, video, cases, and evaluation forms.

Copyright © 2023 Thompson et al. This is an open-access publication distributed under the terms of the Creative Commons Attribution license.

The University of New Mexico School of Medicine Human Research Protections Office granted institutional review board exempt status.

Results

There were 80 participants total, 17 attending the BNGAP Medical Education Conference workshop and 63 attending the workshop at Central New Mexico Community College. Seventy-one participants completed the pre- and postworkshop questionnaire, making the response rate 89%.

Participants' roles were diverse, with six faculty, eight medical students, three residents, and 63 nursing students. Seventy percent identified as women. Among those who reported their race or ethnicity, 45% ($n = 36$) identified as Latina/o/x/e, Hispanic, or of Spanish origin+; 9% ($n = 7$) as African American/Black; 38% ($n = 30$) as White; 6% ($n = 5$) as Asian; and 1% ($n = 1$) as American Indian/Alaska Native.

The Wilcoxon matched-pair signed rank test showed a statistically significant increase when comparing learners' pre- and postworkshop confidence in addressing each learning objective. The results are depicted in the Table.

In terms of qualitative feedback on the postworkshop evaluation form, several respondents noted that they liked the interactive nature of the session and the different teaching modalities. Regarding improvements, participants desired additional skin of color images and a larger font on several slides.

Discussion

We successfully designed and implemented a workshop to help health professional trainees—in particular, nurses, medical students, and residents—understand that SCI patients can develop melanoma and that these melanomas are usually more advanced/aggressive at the time of diagnosis. The evaluations received were overall positive. Our data revealed that learners had an increase in confidence when identifying melanoma in darker skin tones. We hope that by employing the information gained in this workshop, practitioners can perform more careful dermatologic examinations in SCIs (thereby increasing the likelihood of diagnosing melanoma at an earlier stage) as well as educate their SCI patients on the need for continued skin examinations as part of routine health care.

Table. Participants' Confidence in Meeting Learning Objectives

Objective[a]	Preworkshop	Postworkshop
1. Describe the structure and components of the skin.	2.0	4.5
2. Describe the etiology and clinical manifestation of melanoma and its subtypes.	1.0	4.0
3. Recognize various melanoma presentations, especially in darker skin tones.	1.0	4.0
4. List melanoma prevention and treatment options.	2.0	4.0

[a]Rated on a 5-point scale (1 = *no confidence*, 5 = *complete confidence*).

This resource addresses two critical needs within nursing and medical education. First, there are currently no *MedEdPORTAL* publications created with the objective of increasing awareness of melanoma in SCIs. We find this lack of educational material to represent an important gap in dermatology curricula, especially in the context of a high-morbidity disease process that presents uniquely in SCIs. While the typical demographic affected by melanoma is lighter-pigmented individuals (Fitzpatrick types I-III), the morbidity in SCIs is significantly higher and is diagnosed at later stages.[2] This strongly suggests that further educational material needs to be developed for health professionals and the public.

A second important contribution our material makes is in addressing structural inequalities in health professions education and research language. In researching the topic of melanoma in SCIs, we found a trend towards generalizing racial groups without respect for the heterogeneity of pigmentation. Large swaths of people were referred to collectively as Black, Hispanic, or Asian despite the imprecision of such designations. In our material, we have attempted to define SCIs more clearly by use of the Fitzpatrick scale. Continued penetration of more precise descriptions of pigmented skin as well as recognition of self-identification can better align the literature with diversity, equity, and inclusion–focused health care. Notably, the original use of the Fitzpatrick scale was to describe the reaction of lighter-pigmented Caucasian skin to UV therapy, and so, further development and implementation of an SCI-centric scale are needed.[26]

One main challenge to the creation and implementation of this workshop is being aware of how race, ethnicity, skin tone, and skin pathology are often conflated in the research literature and potentially during discussions of the epidemiology of melanoma. We encourage facilitators to help workshop participants review and use terms such as racial categories and skin of color appropriately rather than considering them synonymous.

There are several limitations associated with this workshop. The results should not be overgeneralized to indicate that the workshop would yield the same increase in confidence among

Copyright © 2023 Thompson et al. This is an open-access publication distributed under the terms of the Creative Commons Attribution license.

all medical students, residents, faculty, or nursing students. Our sample was relatively small, with a large representation of nursing students. The workshop is a brief intervention and may not yield sustained confidence in meeting the learning objectives. The workshop has not been designed to assess for changes in knowledge or behavior, components critical to reducing clinical outcomes. Nor has the workshop been trialed using a variety of facilitator knowledge levels; therefore, we suggest that future facilitators have a foundation in basic skin anatomy and dermatopathology. In terms of our evaluation tool, we did not label each response on the 5-point scale, only the anchors. Artino and Gehlbach suggest that all response options, especially for a 5-point scale, should be labeled to accurately measure what is intended to be measured.[27]

Despite these limitations, participants' feedback demonstrated that the workshop structure and content were effective at raising awareness of melanoma and its various presentations. Learners commended the use of videos, reflection exercises, and active interaction through the case discussion. Modifications for improvement of the material in the future could include more images as well as continued efforts to deconstruct race-oriented language in favor of objective pigmentation scales.

Appendices

A. Melanoma Presentation.pptx

B. Melanoma Myth.mp4

C. Facilitator Guide.docx

D. Evaluation Form.docx

All appendices are peer reviewed as integral parts of the Original Publication.

Brandon Thompson, RN, BSN: Registered Nurse, Neuroscience Intensive Care Unit, University of New Mexico Hospital

Toni Jenkins, MS: Third-Year Medical Student, Howard University College of Medicine

John Paul Sánchez, MD, MPH: Vice Chair for DEI, Department of Emergency Medicine, University of New Mexico School of Medicine, and Executive Associate Vice Chancellor, Health Sciences Center Office for Diversity, Equity and Inclusion, University of New Mexico School of Medicine; ORCID: https://orcid.org/0000-0002-6488-4728

Matthew Frederick, DO: Assistant Professor, Department of Emergency Medicine, University of New Mexico School of Medicine

Alba Posligua-Alban, MD: Third-Year Resident, Department of Dermatology, University of New Mexico School of Medicine

Naiara Sbroggio Barbosa, MD: Assistant Professor of Dermatology, Mayo Clinic College of Medicine and Science

Acknowledgments
Thank you to the DEI-Related Academic Medicine Writing Fellowship of the Department of Emergency Medicine of the University of New Mexico School of Medicine for providing financial support and mentorship to complete this project. Thank you to Lisa Leiding, DNP, RN, and Edna Watts, MSN, RN, for offering their lecture time to present this workshop to level 3 and level 4 nursing students at Central New Mexico Community College.

Disclosures
None to report.

Funding/Support
The DEI-Related Academic Medicine Writing Fellowship of the Department of Emergency Medicine of the University of New Mexico School of Medicine provided financial support for this project.

Ethical Approval
The University of New Mexico School of Medicine Human Research Protections Office deemed further review of this project not necessary.

References

1. Aggarwal P, Knabel P, Fleischer AB Jr. United States burden of melanoma and non-melanoma skin cancer from 1990 to 2019. *J Am Acad Dermatol*. 2021;85(2):388-395. https://doi.org/10.1016/j.jaad.2021.03.109
2. Cormier JN, Xing Y, Ding M, et al. Ethnic differences among patients with cutaneous melanoma. *Arch Intern Med*. 2006;166(17):1907-1914. https://doi.org/10.1001/archinte.166.17.1907
3. Anaba EL. Cutaneous malignant melanoma in skin of color individuals. *Niger J Med*. 2021;30(1):1-7.
4. American Cancer Society. *Cancer Facts & Figures for African Americans 2019-2021*. American Cancer Society; 2019. https://www.cancer.org/content/dam/cancer-org/research/cancer-facts-and-statistics/cancer-facts-and-figures-for-african-americans/cancer-facts-and-figures-for-african-americans-2019-2021.pdf
5. Taylor SC. Skin of color: biology, structure, function, and implications for dermatologic disease. *J Am Acad Dermatol*. 2002;46(suppl 2):S41-S62. https://doi.org/10.1067/mjd.2002.120790
6. Gloster HM Jr, Neal K. Skin cancer in skin of color. *J Am Acad Dermatol*. 2006;55(5):741-760. https://doi.org/10.1016/j.jaad.2005.08.063
7. Rahman Z, Taylor SC. Malignant melanoma in African Americans. *Cutis*. 2001;67(5):403-406.

Copyright © 2023 Thompson et al. This is an open-access publication distributed under the terms of the Creative Commons Attribution license.

8. Nelson B. How dermatology is failing melanoma patients with skin of color. *Cancer Cytopathol*. 2020;128(1):7-8. https://doi.org/10.1002/cncy.22229
9. Taylor SC, Elbuluk NM. *Taylor and Elbuluk's Color Atlas and Synopsis for Skin of Color.* McGraw Hill; 2023.
10. Jackson-Richards D, Pandya AG, eds. *Dermatology Atlas for Skin of Color.* Springer; 2014.
11. Eleryan M, Friedman A. *The Full Spectrum of Dermatology: A Diverse and Inclusive Atlas.* SanovaWorks; 2021.
12. Skin of Color Curriculum. American Academy of Dermatology. March 8, 2022. Accessed February 23, 2023. https://learning.aad.org/Listing/Skin-of-Color-Curriculum-5719
13. Dermatology E-Learning + Equity Platform. Skin of Color Society. Accessed February 23, 2023. https://deep.skinofcolorsociety.org/learn
14. Vaseline launches See My Skin using VisualDx technology. VisualDx. April 19, 2022. Accessed February 23, 2023. https://www.visualdx.com/blog/vaseline-launches-see-my-skin-using-visualdx-technology/
15. Inclusive dermatology: creating a diverse visual atlas of skin conditions. University of New Mexico School of Medicine. Accessed February 23, 2023. https://hsc.unm.edu/medicine/departments/dermatology/inclusive-dermatology/
16. Ortega P, López-Hinojosa I, Park YS, Girotti JA. Medical Spanish musculoskeletal and dermatologic educational module. *MedEdPORTAL*. 2021;17:11071. https://doi.org/10.15766/mep_2374-8265.11071
17. Compres E, Holzem K, Ibler E, Bierman JA, Rademaker AW, Kundu RV. A brief dermatology curriculum in skin cancer detection and prevention to improve medical student knowledge and confidence. *MedEdPORTAL*. 2020;16:11049. https://doi.org/10.15766/mep_2374-8265.11049
18. Scaperotti M, Gil N, Downs I, et al. Development and evaluation of a web-based dermatology teaching tool for preclinical medical students. *MedEdPORTAL*. 2017;13:10619. https://doi.org/10.15766/mep_2374-8265.10619
19. Rana J, Mostaghimi A. Introduction to skin cancer: a video module. *MedEdPORTAL*. 2016;12:10431. https://doi.org/10.15766/mep_2374-8265.10431
20. Thomas PA, Kern DE, Hughes MT, Chen BY, eds. *Curriculum Development for Medical Education: A Six-Step Approach*. 3rd ed. Johns Hopkins University Press; 2016.
21. Hansra NK, O'Sullivan P, Chen CL, Berger TG. Medical school dermatology curriculum: are we adequately preparing primary care physicians? *J Am Acad Dermatol*. 2009;61(1):23-29.E1. https://doi.org/10.1016/j.jaad.2008.11.912
22. Liaison Committee on Medical Education. *Functions and Structure of a Medical School: Standards for Accreditation of Medical Education Programs Leading to the MD Degree*. Association of American Medical Colleges/American Medical Association; 2022.
23. Holmboe ES, Iobst WF. *ACGME Assessment Guidebook.* Accreditation Council for Graduate Medical Education; 2020. Accessed February 23, 2023. https://www.acgme.org/globalassets/PDFs/Milestones/Guidebooks/AssessmentGuidebook.pdf?ver=2020-11-18-155141-527&ver=2020-11-18-155141-527
24. Association of American Medical Colleges. *Cultural Competence Education.* Association of American Medical Colleges; 2005. Accessed February 23, 2023. https://www.aamc.org/media/20856/download
25. Association of American Medical Colleges. *Diversity, Equity, and Inclusion Competencies Across the Learning Continuum.* Association of American Medical Colleges; 2022.
26. Okoji UK, Taylor SC, Lipoff JB. Equity in skin typing: why it is time to replace the Fitzpatrick scale. *Br J Dermatol*. 2021;185(1):198-199. https://doi.org/10.1111/bjd.19932
27. Artino AR Jr, Gehlbach H. Avoiding four visual-design pitfalls in survey development. *Acad Med*. 2012;87(10):1452.

Received: March 27, 2022
Accepted: January 14, 2023
Published: May 9, 2023

Copyright © 2023 Thompson et al. This is an open-access publication distributed under the terms of the Creative Commons Attribution license.
